# Modular synthesis of the pyrimidine core of the manzacidins by divergent Tsuji–Trost coupling

**DOI:** 10.3762/bjoc.12.107

**Published:** 2016-06-02

**Authors:** Sebastian Bretzke, Stephan Scheeff, Felicitas Vollmeyer, Friederike Eberhagen, Frank Rominger, Dirk Menche

**Affiliations:** 1 Institut für Organische Chemie, Ruprecht-Karls Universität Heidelberg, Im Neuenheimer Feld 270, 69120 Heidelberg, Germany; 2 Kekulé-Institut für Organische Chemie und Biochemie, Universität Bonn, Gerhard-Domagk-Strasse 1, 53121 Bonn, Germany

**Keywords:** cross-metathesis, natural products, pyrimidines, Tsuji–Trost reaction, synthetic methods

## Abstract

The design, development and application of an efficient procedure for the concise synthesis of the 1,3-*syn-* and *anti*-tetrahydropyrimidine cores of manzacidins are reported. The intramolecular allylic substitution reaction of a readily available joint urea-type substrate enables the facile preparation of both diastereomers in high yields. The practical application of this approach is demonstrated in the efficient and modular preparation of the authentic heterocyclic cores of manzacidins, structurally unique bromopyrrole alkaloids of marine origin. Additional features of this route include the stereoselective generation of the central amine core with an appending quaternary center by an asymmetric addition of a Grignard reagent to a chiral *tert*-butanesulfinyl ketimine following an optimized Ellman protocol and a cross-metathesis of a challenging homoallylic urea substrate, which proceeds in good yields in the presence of an organic phosphoric acid.

## Introduction

**Figure 1:**
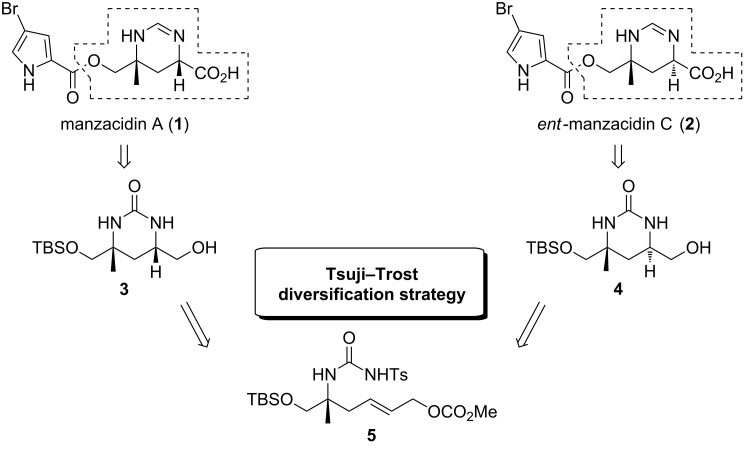
Modular concept for manzacidin synthesis based on a Tsuji–Trost coupling of joint intermediate **5**.

Chiral pyrimidine motifs constitute prevalent structural features in a variety of potent natural products and bioactive agents [1–5]. As exemplified by the marine natural products manzazidins A and C [2–5], they may be characterized by diverse configurations, including synthetically challenging quaternary centers. Owing to their pronounced biological activities, several synthetic routes have been reported to access these important substructures [6–22]. The manzacidins have first been isolated by the group of Kobayashi from the marine sponge *Hymeniacidon* sp. in the early nineties of the last century [2]. The compounds have demonstrated potent antifungal activity [3], and acted as α-adrenoceptor blockers, antagonists of the serotonergic receptor and/or actomyosin ATPase activators [23–25]. As shown in Figure 1 for the most prominent representatives, manzacidins A (**1**) and C (**2**), their unique architecture is characterized by an ester-linked bromopyrrole carboxylic acid and a tetrahydropyrimidine ring in which one of the amino groups is attached to a quaternary carbon center. Due to their intriguing structures in combination with the promising biological properties this class of bromopyrrole alkaloids has attracted great interest from synthetic chemists and a variety of elegant total syntheses has been reported [6–22]. Inspired by an innovative concept for heterocycles synthesis recently developed in our group [26–31], we became interested to devise a novel and a more versatile route to the central heterocyclic core of these marine metabolites. The method is based on a late-stage diversification strategy involving a Tsuji–Trost reaction of the urea-type joint precursor **5**. In contrast to existing routes, this approach enables a more versatile elaboration of different configurations as present in the manzacidins and/or originally postulated for this class of marine natural products. Notably, the absolute configuration of manzacidin C was initially proposed as shown in Figure 1 [2] and subsequently revised by a total synthesis [6] which adds to the importance of a flexible route to such substructures. Herein we report in full detail the design, development and application of an innovative strategy for the high-yielding synthesis of 1,3-*syn*- and *anti*-configured tetrahydropyrimidinones, based on an allylic substitution reaction of a joint precursor **5**. Subsequently this strategy is successfully applied to the synthesis of the authentic pyrimidine cores **3** and **4** of manzacidin A (**1**) and *ent*-manzacidin C (**2**).

## Results and Discussion

### General synthetic concept

**Scheme 1:**
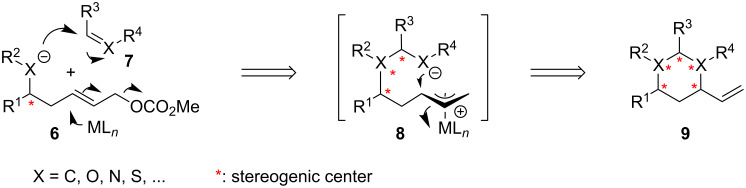
General concept for heterocycles synthesis based on a nucleophilic addition and Tsuji–Trost coupling.

As part of our ongoing efforts to the design of novel tandem reactions for the synthesis of complex natural products [29,32–37], we have developed an innovative concept for heterocycles synthesis [26–31]. As shown in Scheme 1, this approach that further advances and generalizes several individual reports by other groups [38–43], is based on a sequential nucleophilic addition and an intramolecular allylic substitution reaction. It relies on the coupling of different homoallylic nucleophiles of general type **6** to diverse electrophiles **7** such as Michael acceptors, or heteroolefins as for example imines, carbonyls or allene homologs. The resulting homologated nucleophile **8** may then be trapped in an intramolecular fashion by a π-allyl complex, which may concomitantly form from **6** through activation of the homoallylic functionality with a suitable transition metal catalyst. According to this concept, variously substituted 6-membered heterocycles of type **9** may be obtained in a general and concise fashion. Notably, this anionic relay process may directly generate up to four new stereogenic centers and thus demonstrates a high increase in structural complexity from readily available starting materials.

### Evaluation of the concept by a model study

**Scheme 2:**
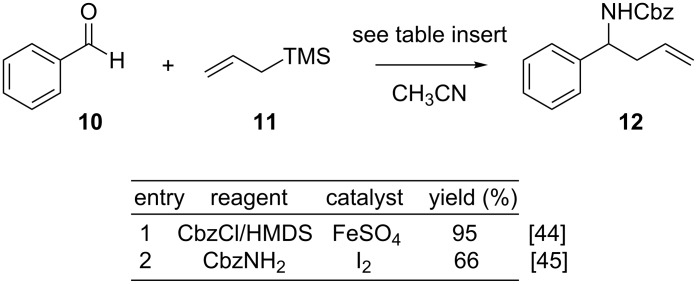
Synthesis of homoallylic alcohol **12** by multi-component reactions.

As a prelude to the targeted substitution pattern of the manzacidins, we first evaluated the applicability of this process for a modular synthesis of 1,3-*syn*- and *anti*-tetrahydropyrimidinones using the simplified amine substrate **12**. Parts of this model study have already been reported in preliminary form [31]. Homoallylic amines of type **12** may be efficiently obtained through multicomponent reactions. These involve the nucleophilic allylation of imines which may be generated in situ by the condensation of an amine and a carbonyl compound. As shown in Scheme 2, two such procedures were evaluated within the preliminary study. The first protocol that we analyzed was reported by the group of Tian. It involves a four-component coupling of aldehyde **10** with CbzCl for activation of the nitrogen source, HMDS and allyltrimethylsilane (**11**) in the presence of catalytic amounts of FeSO_4_ [44]. In our hands, this process enabled an efficient access to the desired homoallylic amine **12** in essentially quantitative yields. The other protocol was reported by Phukan and involves an iodine-catalyzed condensation of aldehyde **10** with benzylcarbamate and allyltrimethylsilane (**11**) [45]. Unfortunately, this route was found to be less effective in terms of isolated yields and scalability. Thus, the iron-catalyzed procedure was applied and multigram quantities of **12** were readily obtained.

**Scheme 3:**
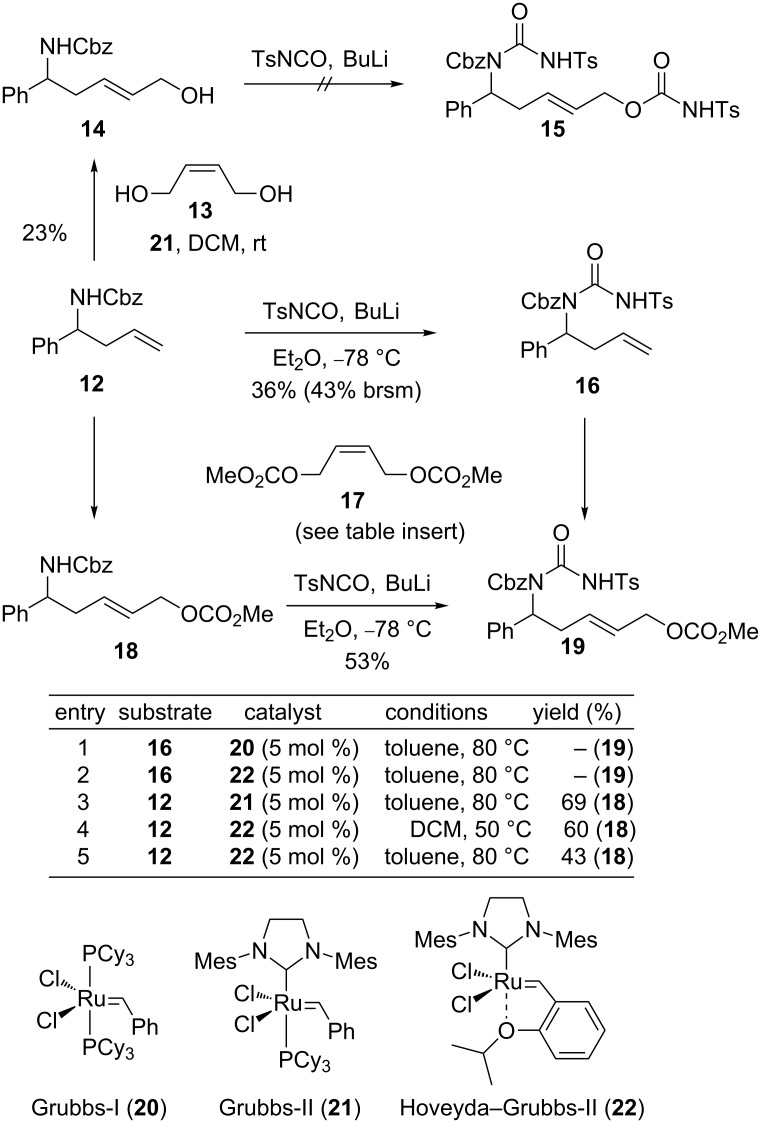
Preparation of urea-type cyclization precursor **19**.

As shown in Scheme 3, we next focused on the further derivatization of amine **12** towards suitably functionalized urea substrates **15** or **19**. Inspired by a work of Garcia [39], we initially intended to use isocyanate for both, the introduction of the urea motif and for the functionalization of the terminal homoallylic alcohol. Consequently, we evaluated the conversion of **14** to **15**. The required substrate **14** was prepared from amine **12** by cross-metathesis with 2-butene-1,4-diol (**13**) in the presence of Grubbs-II catalyst **21**. However, in the subsequent coupling reactions of **14** with TsNCO it became apparent that this homoallylic amine was too unreactive to enable a double addition to access **15** directly. Therefore, a stepwise approach towards **19** was pursued instead. This involved either a coupling of **12** first with isocyanate to give **16** followed by a cross-metathesis or starting with the cross-metathesis to **18** and subsequent installment of the urea motif. As shown in the table inserted in Scheme 3 for selected cross-metatheses of Cbz-protected amide **12** and its urea-derivative **16** with butene **17**, a different reactivity of **12** and **16** was observed. While **16** proved too unreactive for the coupling reaction under various conditions (e.g., entries 1 and 2), the homologation of the Cbz-protected amine **12** to **18** could be realized. Preparative useful yields (69%) were obtained with Grubbs-II catalyst (**21**) in toluene at elevated temperatures (entry 3), while lower conversions were observed with other catalysts (**20**, **22**) or in dichloromethane (entries 4 and 5). Finally, for the installment of the required urea motif into **18**, tosylisocyanate in combination with strong bases was required to achieve useful degrees of conversion towards the desired precursor **19**. The best results were obtained with BuLi, as previously communicated [31], while weaker bases (NEt_3_, LHMDS, DBU, proton sponge) and less electron-deficient isocyanates resulted in lower yields.

**Scheme 4:**
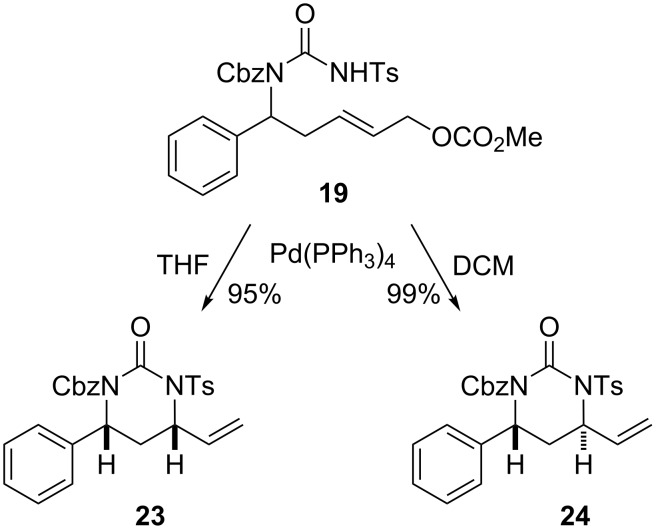
Stereodivergent synthesis of 1,3-*syn*- and *anti*-tetrahydropyrimidinones [31].

We then turned our attention to the pivotal intramolecular allylic substitution reaction of **19** to access *syn*- and *anti*-pyrimidinones **23** and **24**. As previously reported [31], this diastereodivergent coupling could indeed be realized as shown in Scheme 4. Based on a report of Garcia for a related system we first evaluated Pd_2_(dba)_3_ with different phosphite ligands [39]. However, the best results were obtained with the stable catalyst Pd(PPh_3_)_4_ and depending on the solvent used, either the *syn*-isomer **23** or the *anti*-isomer **24** could be selectively obtained.

### Application of the concept for manzacidin core synthesis

**Scheme 5:**
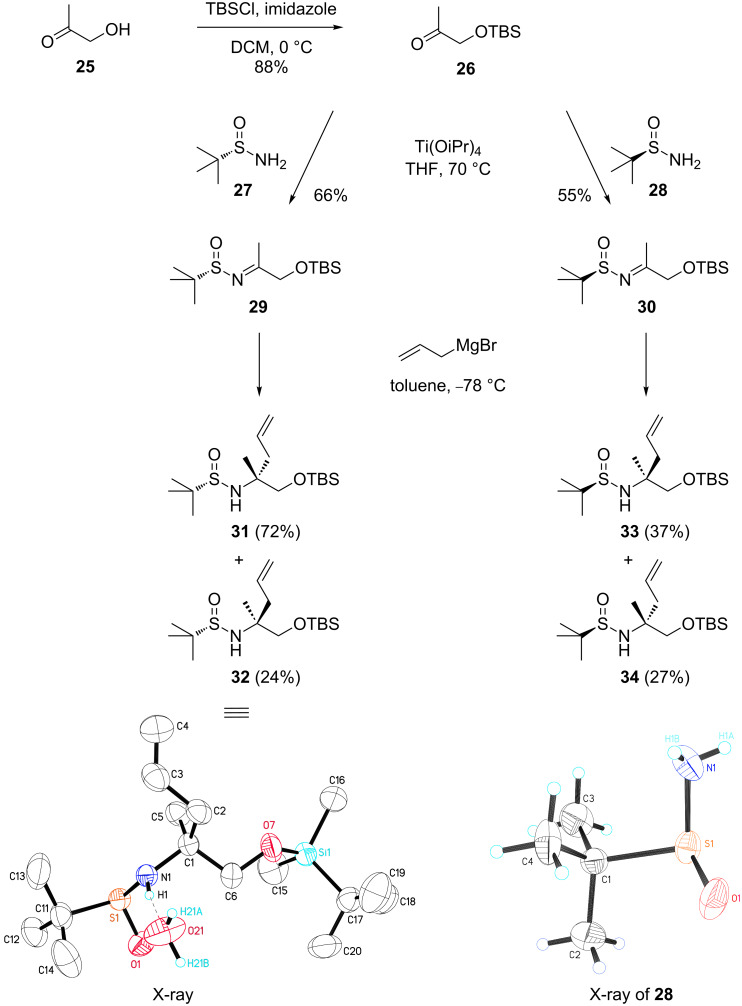
Stereoselective synthesis of all possible stereoisomers of the manzacidin core amine by asymmetric addition to chiral *tert*-butanesulfinyl ketimines.

**Scheme 6:**
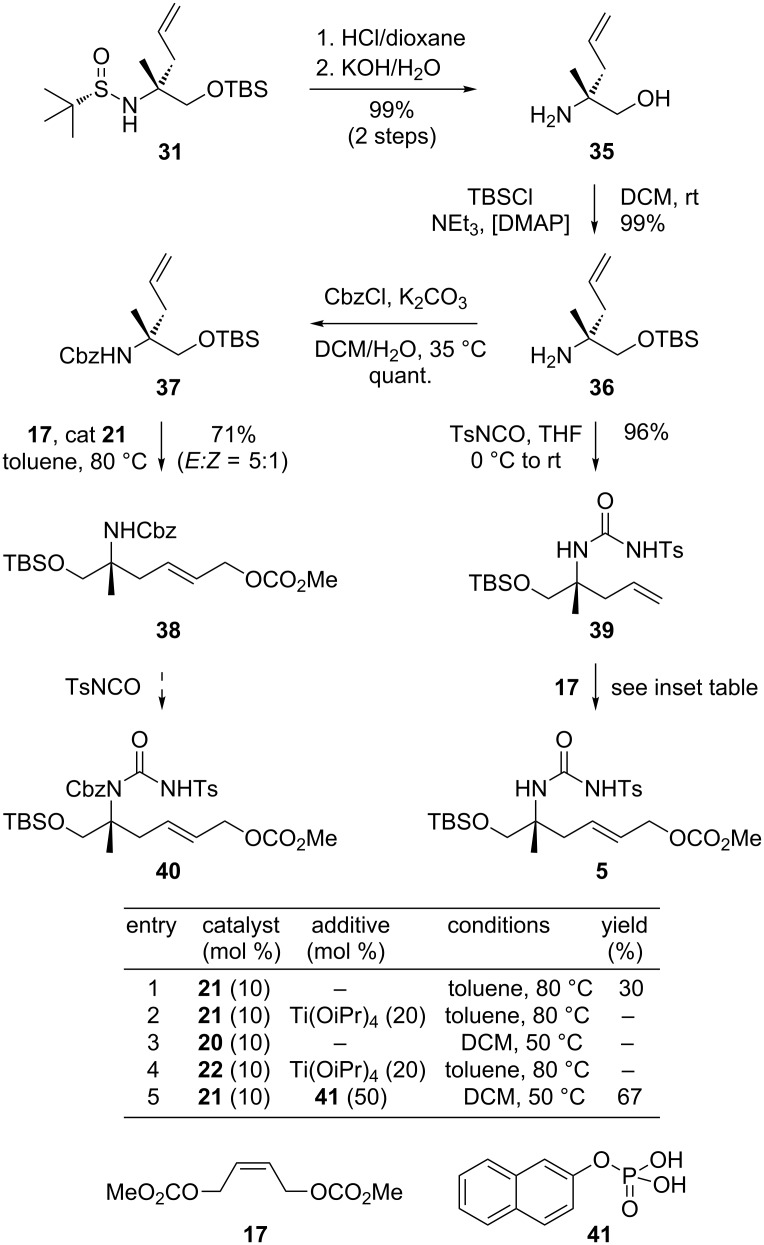
Synthesis of the authentic cyclization precursor **5**.

After proofing the general adaptability of our synthetic concept, we next evaluated the applicability of this procedure for the synthesis of the authentic manzacidin substrate. As shown in Scheme 5, we first focused on the stereoselective synthesis of the chiral amine core of these alkaloids. For the synthesis of the nitrogen appending the quaternary center we tested a method developed by the Ellman group [46–47], which relies on an asymmetric addition of organometallic reagents to enantiopure *tert*-butanesulfinyl ketimines of type **29** and **30**. Although the group of Lee had already communicated the synthesis of **33** using this approach [48], no full details have been given. In addition, the reported yields were only moderate and the stereochemistry appeared not to have been rigorously assigned. Therefore, we evaluated this type of asymmetric addition in more general terms and analyzed the addition reactions of allylmagnesium bromide both to **29** and **30**. Notably, this route would allow to access all possible stereoisomers of the manzacidins, in agreement with the stereochemical diversity of this class of natural products. In detail, the synthesis of **29** and **30** involved a condensation of hydroxyacetone (**25**)-derived ketone **26** [49] with *S*_S_- and *R*_S_-*tert*-butanesulfinamides **27** and **28**, respectively. As an improvement to the original procedure [46–48], we applied Ti(OiPr)_4_ as Lewis acid instead of the reported Ti(OEt)_4_, which resulted in higher yields and a more reliable process in our hands. In agreement with the results of Lee the addition of allylmagnesium bromide to **30** lead to **33** in only moderate yields and low selectivity towards **34**. We then studied the coupling of **29** in more detail to target amine **31** that bears the correct configuration required for manzacidin A. Possibly, the higher selectivity observed for the conversion of **29** as compared to **30** may be due to initial problems during the work-up. Finally, the addition could be effected giving the desired diastereomer **31** in high yields (72%) and the minor isomer **32** that was likewise obtained (24%) could be readily removed by column chromatography. The configuration of **31** was initially assigned by Mosher ester analysis of the free amine **36** (Scheme 6) and finally proven in an indirect manner by an X-ray crystallography of the minor diastereomer **32**. Within the course of this study also an X-ray structure of *tert*-butylsulfinylamine **28** was obtained. Remarkably, these types of substances have not been broadly evaluated by X-ray structural analysis which adds to the importance of this general evaluation.

**Figure 2:**
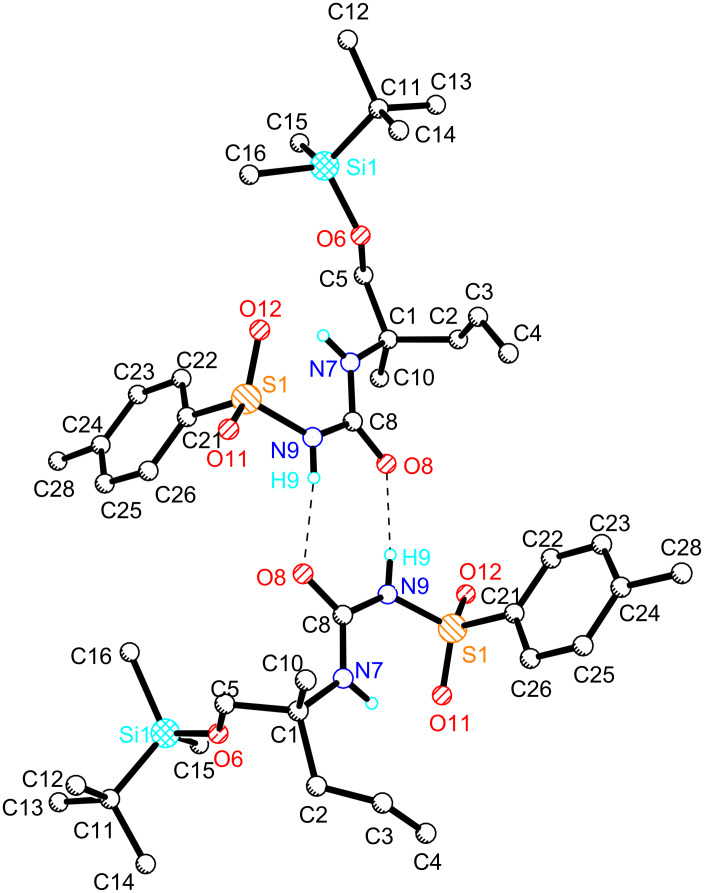
X-ray structure of **39**.

Next, we focused on further homologation towards a suitably functionalized urea precursor **5** for the envisioned Tsuji–Trost cyclization. As shown in Scheme 6, this involved an acidic cleavage of the sulfinamide followed by basic treatment to give free amine **35**. After protection of the primary hydroxy group as TBS ether, we first evaluated the synthesis of derivative **40**, in analogy to our model study. Accordingly, the free amine **36** was Cbz-protected following the Schotten–Baumann method [50]. The obtained amide **37** was then homologated by cross-metathesis with butenedicarboxylate **17** in the presence of Grubbs-II catalyst (**21**) applying our conditions developed above (Scheme 3). However, with the resulting homologated amide **38** in hand we were not able to install the required urea moiety with tosylisocyanate, despite considerable efforts with various bases, solvents or variation of temperature and equivalents. These results again demonstrated the difficulties to install the urea function in a sterically hindered and electronically unreactive Cbz-protected amine substrate, which is in agreement with our observations above. Therefore, we decided to continue our route with the free amine **36** instead, which was directly coupled with TSNCO to give **39** in high yield. The reaction took place even without an additional base, which shows the strong influence of the amine protective group on this type of condensation. Importantly, at this stage, the structure of **39** was fully confirmed by X-ray crystallography. As shown in Figure 2, this urea derivative is present as an unsymmetrical dimer in the crystal lattice, which is stabilized by two hydrogen bonds between the urea oxygen atoms and the tosyl-protected nitrogens. This also unambiguously confirms the absolute configuration of **39** and corroborates our prediction of the asymmetric adduct **31**.

We then attempted the installation of the required allylic carbonate on **39** by cross-metathesis with **17**. However, initial attempts following our protocol developed above with Grubbs-II catalyst (**21**) resulted in only moderate conversion (inserted table in Scheme 6, entry 1). Also, the application of other catalysts with or without additional additives to impede a possibly unfavorable amine coordination of the reactive ruthenium intermediates [51] did not improve the reaction outcome (entries 2–4). Following reports from Nolan and Prunet [52], as well as from Steinke and Vilar [53] we finally evaluated tricyclohexylphosphane oxides and organic phosphoric acid, which had been reported to have beneficial effects in the cross-metathesis of related substrates. In the presence of catalytic amounts of phosphoric acid **41** [53], the coupling of **39** with **17** could indeed be realized in useful yields in a reliable fashion. Optimal results included treatment of **39** with 2.5 equiv of dicarbonate **17**, 50 mol % naphthylphosphoric acid and 10 mol % Grubbs-II catalyst, giving the desired urea derivative **5** in good yield (67%), considering the general difficulties observed for such substrates in cross-metathesis reactions.

**Scheme 7:**
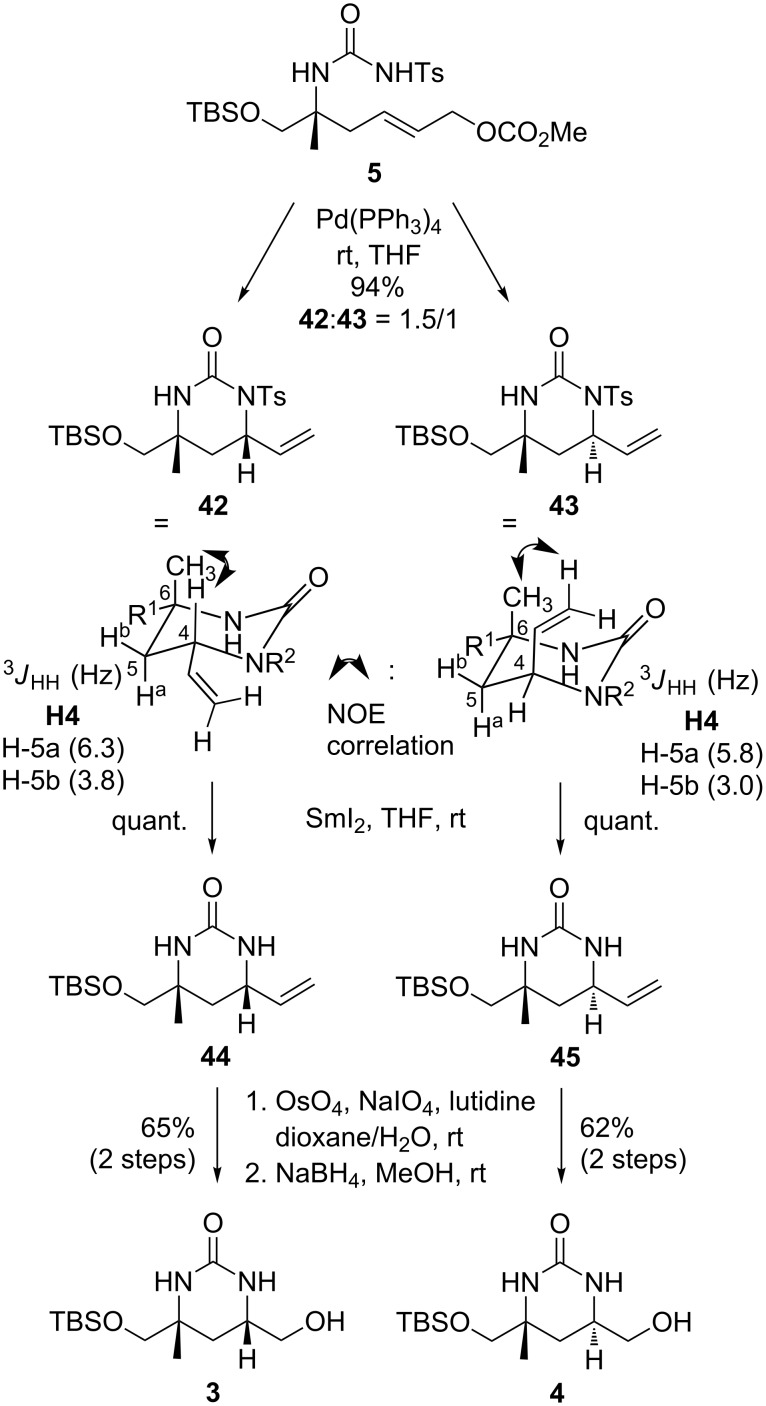
Divergent Tsuji–Trost coupling and completion of the synthesis of authentic pyrimidinones **3** and **4**.

With precursor **5** in hand the desired cyclization towards **42** and **43** could then be efficiently realized in a straightforward manner giving the desired *syn*- and *anti*- tetrahydropyrimidinones in a joint fashion with a ratio of 1.5:1. Following the protocol developed above, excellent yields (94%) were obtained in this coupling. As compared to the model substrate **19** (see Scheme 4) no selectivity was observed in this coupling, which could also not be modified by other solvents. Possibly this may be due to the missing Cbz group of **5** as compared to **19**. The configuration of both products was assigned by NMR methods based on characteristic NOE correlations and vicinal coupling constants as shown in Scheme 7. For further conversion to key intermediates **3** and **4**, the tosyl groups of **42** and **43** were removed with SmI_2_ [54–55] giving the free amides **44** and **45**. The terminal double bonds were then oxidized by dihydroxylation with OsO_4_ and periodate cleavage [56–57], and the resulting aldehydes (not shown) were reduced to the terminal alcohols with NaBH_4_, giving the desired pyrimidinones **3** and **4**. These compounds represent key intermediates which may be transformed into the targeted natural products **1** and **2** following previously established protocols [6,31].

## Conclusion

In summary, we have reported in full details the design, development and application of an efficient method for the synthesis of the tetrahydropyrimidinone core of the manzacidins by a divergent intramolecular allylic substitution reaction. The application of this approach enabled a highly concise access to the authentic heterocyclic cores of the manzacidins, structurally unique natural products of marine origin. Additional notable features of our modular route also include the generation of an amine appending quaternary center by an optimized Ellman protocol for the asymmetric allyl-Grignard addition to enantiopure *tert*-butanesulfinyl ketimines and an efficient cross-metathesis of an unreactive urea substrate in the presence of an organic phosphoric acid. It is expected that these strategies and tactics will find applications in functional target synthesis and stimulate further studies for modular heterocycle synthesis.

## Experimental

### Preparation of **31** by asymmetric addition of allylmagnesium bromide to **29**

In a flame-dried flask (*S*_S_)-*N*-(1-((*tert*-butyldimethylsilyl)oxy)propan-2-ylidene)-2-methylpropane-2-sulfinamide (**29**, 1.37 g, 4.70 mmol, 1.0 equiv) was dissolved in 12 mL toluene and the solution was cooled to −78 °C. To this mixture allylmagnesium bromide (1.0 M in Et_2_O, 7.05 mL, 7.05 mmol, 1.5 equiv) was slowly added and the reaction was stirred for 1 h at −78 °C. The reaction was quenched with a solution of saturated Na_2_SO_4_, warmed to rt, filtered, washed with ethyl acetate and finally purified by column chromatography on silica gel (120 g) with ethyl acetate/hexane 1:9 as eluent, which yielded the desired diasteromers **31** (major diastereomer) and **32** (minor diastereomer) as light yellow oils (96 %, dr = 1:3). Major diastereomer *S*_S_*R* (1.13 g, 3.39 mmol, 72%): *R*_f_ 0.1 (ethyl acetate/hexane 1:9); [α]^20^_D_ +58.2 (*c* 1.00, CHCl_3_); ^1^H NMR (300.13 MHz, CDCl_3_) δ 0.06 (s, 6H), 0.91 (s, 9H), 1.19 (s, 12H), 2.48 (dd, *J* = 4.1 Hz, 7.4 Hz, 2H), 3.32 (d, *J* = 9.3 Hz, 1H), 3.49 (d, *J* = 9.3 Hz, 1H), 3.72 (bs, 1H), 5.11 (d, *J* = 10.4 Hz, 1H), 5.12 (d, *J* = 16.7 Hz, 1H), 5.80 (ddt, *J* = 7.4 Hz, 10.4 Hz, 17.8 Hz, 1H); ^13^C NMR (75.47 MHz, CDCl_3_) δ −5.5, 18.2, 22.1, 22.6, 25.8, 43.0, 55.5, 58.1, 69.2, 118.7, 133.8; HRMS–FAB (*m*/*z*): [M + H]^+^ calcd for C_16_H_36_NO_2_SSi, 334.2231; found, 334.2227. Minor diastereomer *S*_S_*S* (371 mg, 1.11 mmol, 24%): *R*_f_ 0.13 (ethyl acetate/hexane 1:9); [α]^20^_D_ +39.2 (*c* 1.00, CHCl_3_); ^1^H NMR (300.13 MHz, CDCl_3_) δ 0.07 (s, 3H), 0.08 (s, 3H), 0.91 (s, 9H), 1.20 (s, 9H), 1.28 (s, 3H), 2.22 (dd, *J* = 8.0 Hz, 13.7 Hz, 1H), 2.37 (dd, *J* = 6.7 Hz, 14.0 Hz, 1H), 3.48 (d, *J* = 9.3 Hz, 1H), 3.53 (d, *J* = 9.6 Hz, 1H), 3.77 (bs, 1H), 5.09 (d, *J* = 17.8 Hz, 1H), 5.10 (d, *J* = 10.7 Hz, 1H), 5.78 (ddt, *J* = 8.0 Hz, 10.7 Hz, 17.3 Hz, 1H); ^13^C NMR (75.47 MHz, CDCl_3_) δ −5.6, 18.2, 22.3, 22.7, 25.8, 43.1, 55.6, 58.1, 69.9, 118.5, 133.6; HRMS–ESI (*m*/*z*): [M + H]^+^ calcd for C_16_H_36_NO_2_SSi, 334.2231; found, 334.2231.

### Preparation of **39** by addition of TsNCO to amine **36**

*p*-TsNCO (0.6 mL, 4.13 mmol, 1.1 equiv) was slowly added to a stirred solution of (*R*)-1-((*tert*-butyldimethylsilyl)oxy)-2-methylpent-4-en-2-amine (**36**, 902 mg, 3.93 mmol) in dry THF (3.9 mL) at 0 °C and stirring was continued at rt for 5 h. The solvent was removed under reduced pressure and purification of the residue by column chromatography on silica gel (cyclohexane/ethyl acetate 4:1) yielded the desired product (1.61 g, 3.77 mmol, 96%) as a colorless solid. *R*_f_ 0.29 (cyclohexane/ethyl acetate 4:1); mp 84 °C; [α]^20^_D_ −2.3 (*c* 0.5, CHCl_3_); ^1^H NMR (300.13 MHz, CDCl_3_) δ 0.09 (s, 6H), 0.93 (s, 9H), 1.24 (s, 3H), 2.43 (d, *J* = 8.0 Hz, 2H), 2.44 (s, 3H), 3.44 (d, *J* = 9.8 Hz, 1H), 3.58 (d, *J* = 9.8 Hz, 1H), 4.99 (dd, *J* = 10.1, 2.1 Hz, 1H), 5.04 (dd, *J* = 17.2, 2.1 Hz, 1H), 5.58 (ddt, *J* = 17.2, 10.1, 8.0 Hz, 1H), 6.84 (brs, 1H), 7.30 (d, *J* = 8.1 Hz, 2H), 7.77 (d, *J* = 8.1 Hz, 2H); ^13^C NMR (75.47 MHz, CDCl_3_) δ −5.4, 18.5, 21.4, 21.8, 26.0, 40.1, 57.3, 67.8, 118.8, 127.2, 129.9, 133.3, 137.0, 144.7, 150.3; HRMS–ESI (*m*/*z*): [M + Na]^+^ calcd for C_20_H_34_N_2_NaO_4_SSi, 449.1901; found, 449.1892. CCDC 1461909 (**39**) contains the supplementary crystallographic data for this paper. These data can be obtained free of charge from The Cambridge Crystallographic Data Centre via http://www.ccdc.cam.ac.uk/data_request/cif.

### Preparation of **5** by cross-metathesis of **39** with **17**

To a solution of (*R*)-*N*-((1-((*tert*-butyldimethylsilyl)oxy)-2-methylpent-4-en-2-yl)carbamoyl)-4-methylbenzenesulfonamide (**39**, 50.0 mg, 0.12 mmol, 1.0 equiv), (*Z*)-(but-2-ene-1,4-diyl)dimethyl dicarbonate (**17**, 60.0 mg, 0.29 mmol, 2.5 equiv) and naphthylphosphoric acid (**41**, 15.0 mg, 67.0 µmol, 0.5 equiv) in dry and degassed dichloromethane (1 mL) was added Grubbs-II catalyst (**21**, 10.0 mg, 11.8 µmol, 10 mol %) and the resulting mixture was stirred overnight at 50 °C under an argon atmosphere. Concentration in vacuo and purification by column chromatography on silica gel (10 g) with ethyl acetate/hexane 1:9 as eluent yielded the desired allylic carbonate as brown oil (41.3 mg, 80.4 µmol, 67%): *R*_f_ 0.30 (ethyl acetate/hexane 1:3); [α]^20^_D_ −2.9 (*c* 1.00, CHCl_3_); ^1^H NMR (300.13 MHz, CDCl_3_) δ 0.09 (s, 6H), 0.93 (s, 9H), 1.24 (s, 3H), 2.44 (m, 5H), 3.44 (d, *J* = 9.8 Hz, 1H), 3.56 (d, *J* = 9.8 Hz, 1H), 3.79 (s, 3H), 4.49 (d, *J* = 4.7 Hz, 2H), 5.60 (m, 2H), 6.87 (s, 1H), 7.31 (d, *J* = 8.1 Hz, 2H), 7.77 (d, *J* = 8.3 Hz, 2H), 8.42 (bs, 1H); ^13^C NMR (75.47 MHz, CDCl_3_) δ −5.6, 18.3, 21.3, 21.6, 25.8, 38.5, 54.8, 57.1, 67.7, 68.2, 127.0, 127.5, 129.9, 131.0, 136.7, 144.8, 149.8, 155.6; HRMS–ESI (*m*/*z*): [M + H]^+^ calcd for C_23_H_39_N_2_O_7_SSi, 515.2242; found, 515.2247; HRMS–ESI (*m*/*z*): [M + Na]^+^ calcd for C_23_H_38_N_2_O_7_SSiNa, 537.2061; found, 537.2065.

### Tsuji–Trost coupling of **5** to **42** and **43**

A solution of Pd(PPh_3_)_4_ (432 mg, 3.73 µmol, 20 mol %) in dry THF (300 mL) was added to a stirred solution of (*R*,*E*)-6-((*tert*-butyldimethylsilyl)oxy)-5-methyl-5-(3-tosylureido)hex-2-en-1-ylmethyl carbonate (**5**, 959 mg, 1.86 mmol) in dry THF (300 mL) at rt and stirring was continued for 18 h until the color of the solution changed from yellow to red. The solvent was removed under reduced pressure and purification of the residue by column chromatography on silica gel (cyclohexane/ethyl acetate 4:1) yielded the desired products **42** and **43** (766 mg, 1.75 mmol, 94%, dr 1:1.5 *anti*/*syn*) as off-white solids. **42**: mp 129 °C; [α]^20^_D_ −20.3 (*c* 0.5, CHCl_3_); ^1^H NMR (400.13 MHz, CDCl_3_) δ 0.00 (s, 6H), 0.86 (s, 9H), 1.18 (s, 3H), 1.92 (dd, *J* = 14.2, 6.3 Hz, 1H), 2.10 (dd, *J* = 14.2, 3.6 Hz, 1H), 2.39 (s, 3H), 3.35–3.43 (m, 2H), 5.12–5.16 (m, 1H), 5.18 (dd, *J* = 10.5, 1.5 Hz, 1H), 5.25 (dd, *J* = 17.2, 1.5 Hz, 1H), 5.45 (brs, 1H), 5.79 (ddd, *J* = 17.2, 10.5, 5.4 Hz, 1H), 7.25 (d, *J* = 8.3 Hz, 2H), 7.89 (d, *J* = 8.3 Hz, 2H); ^13^C NMR (100.62 MHz, CDCl_3_) δ −5.4, −5.4, 18.2, 21.7, 25.9, 27.1, 35.8, 55.1, 56.3, 69.1, 116.8, 129.0, 129.1, 137.1, 137.2, 144.2, 151.4; HRMS–EI (*m*/*z*): [M – C_4_H_9_]^+^ calcd for C_17_H_25_N_2_NaO_7_SSi, 381.1304; found, 381.1307. **43**: mp 127 °C; [α]^20^_D_ −12.8 (*c* 0.5, CHCl_3_); ^1^H NMR (400.13 MHz, CDCl_3_) δ 0.02 (s, 3H), 0.03 (s, 3H), 0.85 (s, 9H), 1.20 (s, 3H), 1.89 (dd, *J* = 13.9, 3.2 Hz, 1H), 2.05 (dd, *J* = 13.9, 5.8 Hz, 1H), 2.05 (dd, *J* = 13.9, 5.8 Hz, 1H), 2.39 (s, 3H), 3.25 (d, *J* = 9.4 Hz, 1H), 3.34 (d, *J* = 9.4 Hz, 1H), 5.30–5.17 (m, 4H), 5.88 (ddd, *J* = 16.9, 10.5, 6.0 Hz, 1H), 7.25 (d, *J* = 8.3 Hz, 2 H), 7.90 (d, *J* = 8.3 Hz, 2H); ^13^C NMR (100.62 MHz, CDCl_3_) δ −5.4, −5.5, 18.3, 21.7, 25.6, 25.9, 36.3, 55.0, 56.3, 71.6, 117.0, 129.0, 129.2, 137.1, 144.2, 151.3; HRMS–EI (*m*/*z*): [M – C_4_H_9_]^+^ calcd for C_17_H_25_N_2_NaO_7_SSi, 381.1307; found, 381.1309.

## Supporting Information

File 1 Full experimental details, characterization data of all products, copies of ^1^H and ^13^C NMR spectra and X-ray crystallographic data for **28**, **32** and **39**.
