# Supplementary material for: Modular synthesis of the pyrimidine core of the manzacidins by divergent Tsuji–Trost coupling
Source: Beilstein J Org Chem. 2016 Jun 2;12:1111–21. doi: 10.3762/bjoc.12.107 (PMC4902044; doi:10.3762/bjoc.12.107)
Supplement: File 1 — Full experimental details, characterization data of all products, copies of 1H and 13C NMR spectra and X-ray crystallographic data for 28, 32 and 39. [file Beilstein_J_Org_Chem-12-1111-s001.pdf]

Supporting Information

for

Modular synthesis of the pyrimidine core of the manzacidins by divergent Tsuji–Trost coupling

Sebastian Bretzke[1], Stephan Scheeff[2], Felicitas Vollmeyer[2], Friederike Eberhagen[2], Frank Rominger[1] and Dirk Menche*[2]

Address: [1]Institut für Organische Chemie, Ruprecht-Karls Universität Heidelberg, Im Neuenheimer Feld 270, 69120 Heidelberg, Germany and [2]Kekulé-Institut für Organische Chemie und Biochemie, Universität Bonn, Gerhard-Domagk-Strasse 1, 53121 Bonn, Germany

Email: Dirk Menche* - dirk.menche@uni-bonn.de
* Corresponding author

This article is dedicated to the memory of Peter Hofmann. With deep gratitude I remember the joint time at the University of Heidelberg. He has been a role model in many ways.

Full experimental details, characterization data of all products, copies of ^{1}H and ^{13}C NMR spectra and X-ray crystallographic data for 28, 32 and 39

1. General Information... S3
2. Experimental Section S5
3. NMR Spectra...S18
4. X-RAY Data Analysis S64

1. General Information

Reaction handling: All reactions were performed under argon atmosphere in flame-dried glassware which had been cooled under argon unless stated otherwise. All flasks were equipped with rubber septa and reactants were handled using standard Schlenk techniques. Temperatures above rt (23 °C) refer to oil bath temperatures which were controlled by a temperature modulator. For cooling, the following baths were used: ethanol/liquid nitrogen (−98 °C), acetone/dry ice (−78 °C), water/ice (0 °C). Reactions were magnetically stirred and monitored by (TLC) unless otherwise noted.

Solvents and reagents: Unless stated otherwise, solvents were purchased from the central chemical store of the Chemistry Department of the University of Heidelberg and were distilled. Dry solvents (dichloromethane, THF, toluene, acetonitrile and diethyl ether) were taken out of the solvent purification system MB SPS-800 with drying columns of the University of Heidelberg or purchased over molecular sieves from the following companies: Sigma-Aldrich, Acros-Organics, Fluka, Merck. Unless stated otherwise all of these chemicals were used without further purification.

TLC-analyses: Analytical thin layer chromatography (TLC) was carried out with "Polygram® Sil G/UV254" plastic sheets from Machery-Nagel GmbH & Co. KG. Detection was carried out using short wave UV light (254 nm and 366 nm), cerium (1% $Ce(SO_4)_2$, 2.5% $(MoO_3)_{12}(H_3PO_4)$, 8 mL conc. H_2SO_4 in 100 mL H_2O), permanganate (0.6% $KMnO_4$ in water, with 1% K_2CO_3), vanillin (1–2 g of vanillin in 100 mL of EtOH, containing 1 mL of conc. H_2SO_4) or phosphomolybdic acid (PMA, 200 mL water, 4.84 g $(NH_3)_3PMo_{12}O_{40}$, 3 mL H_3PO_4).

Column chromatography: Flash column chromatography was accomplished using silica gel S (pore size 60 Å, 40–63 µm) purchased from Sigma-Aldrich Chemie GmbH & Co. KG. The yields given refer to the purified products.

Optical Rotations were measured with a Perkin Elmer 241 polarimeter in a 1 dm cuvette, using a sodium lamp. The optical rotation at the sodium D-line ($[\alpha]_D$ value) was calculated according to the Drude equation:

$$[\alpha]_D^T = \frac{\alpha \cdot 100}{c \cdot d} \qquad A = \frac{\alpha_{578}}{\alpha_{546} - \alpha_{578}} \qquad [\alpha]_D^T = \frac{A \cdot \alpha_{546}}{A + 1.3727}$$

T = temperature [°C], *d* = path length [dm], α = measured rotation [°], *c* = concentration [g/100 mL], λ = wavelength [nm].

Melting points were measured on a Büchi B-540 melting point apparatus using open glass capillaries.

^{1}H NMR spectroscopy: ^{1}H NMR spectra were recorded at room temperature using the following spectrometers: 200 MHz: Bruker DRX-200; 250 MHz: Bruker ARX-250; 300 MHz: Bruker AC-300 or Bruker DRX-300; 500 MHz: Bruker DRX-500. Unless stated otherwise all spectra were recorded at room temperature in deuterochloroform which was purchased by Sigma Aldrich and all chemical shifts are given in δ units relative to $CHCl_3$ (singlet: δ^H = 7.27). Data for ^{1}H NMR spectra are reported as follows: chemical

shift (multiplicity, coupling constants in hertz, number of hydrogens), for ^{13}C NMR: chemical shift. Analyses followed first order and the following abbreviations were used throughout: s = singlet, bs = broad singlet, d = doublet, t = triplet, q = quartet, quin = quintet, sxt = sextet, sept = spt, dd = doublet of doublet, dt = doublet of triplet, m = multiplet, m_c= centered multiplet.

^{13}C NMR spectroscopy: ^{13}C NMR spectra were recorded at room temperature using the following spectrometers: 75 MHz: Bruker AC-300 or Bruker DRX-300; 125 MHz: Bruker DRX-500. Unless stated otherwise all spectra were recorded at room temperature in deuterochloroform which was purchased by Sigma Aldrich and all chemical shifts are given in δ units relative to $CDCl_3$ (central line of triplet: δ^C = 77.00).

Mass spectra: Mass spectra (MS) and high resolution mass spectra (HRMS) were recorded at the Department of Organic Chemistry of the University of Heidelberg by Dr. Gross and his members of staff using the following mass spectrometers: Bruker ICR APEX-QE, Vacuum Generators ZAB-2F, Finnigan MAT TSQ 700 and JEOL JMS-700. Ionization processes and calculated exact mass were given.

X-ray diffraction: X-ray diffraction experiments have been carried out by Dr. F. Rominger at the Department of Organic Chemistry at the University of Heidelberg.

Synthesis and characterization data for compounds 12, 17, 18, 19, 23 and 24: Synthesis and characterization data for compounds **12**, **17**, **18**, **19**, **23** and **24** have already been reported in a preliminary publication.[1] These data and copies of NMR spectra for these compounds can be found in the Supporting Information file of this publication.

----

[1] Morgen, M.; Bretzke, S.; Li, P.; Menche, D. *Org. Lett.* **2010,** *12,* 4494-4497.

2. Experimental Section

(*E*)-Benzyl 5-hydroxy-1-phenylpent-3-enylcarbamate (14)

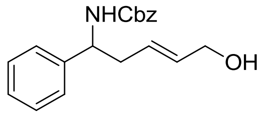

Chemical Formula: $C_{19}H_{21}NO_3$
Exact Mass: 311.15214
Molecular Weight: 311.37494

In a flame-dried and light-protected Schlenk flask, benzyl 1-phenylbut-3-enylcarbamate (**12**, 1.41 g, 5.00 mmol, 1.00 equiv) and Grubbs catalyst 2nd generation (**21**, 212 mg, 0.05 equiv) was diluted in 100 mL of dry dichloromethane at rt under an atmosphere of argon. To this solution was added *cis*-but-2-ene-1,4-diol (**13**, 2.20 g, 2.06 mL, 25.0 mmol, 5.00 equiv) dropwise. The reaction mixture was stirred at rt over night. The solvent was evaporated under vacuum and the residue was purified by column chromatography on silica gel using hexane/ethyl acetate 1:1 as eluent, to afford the desired product as a white solid in a yield of 23% (353 mg, 1.13 mmol). R_f: 0.37 (hexane/ethyl acetate 1:1) ; ^{1}H-NMR (300.13 MHz, $CDCl_3$): δ = 2.56 (t, *J* = 6.22 Hz, 2H), 4.05 (t, *J* = 4.80 Hz, 2H), 4.75 – 4.86 (m, 1H), 5.02 – 5.15 (m, 3H), 5.57 (dt, *J* = 15.26, 6.78 Hz, 1H), 5.72 (dt, *J* = 15.45, 5.46 Hz, 1H), 7.22 – 7.43 (m, 10H) ; ^{13}C-NMR (75.47 MHz, $CDCl_3$): δ = 39.45, 54.74, 63.25, 66.83, 126.23, 127.43, 128.17, 128.50, 128.63, 132.89, 136.35, 155.67 ; HR-MS (ESI): calculated for $C_{19}H_{21}NO_3Na^+$ $[M+Na]^+$: *m/z* = 334.14191, found: *m/z* = 334.14181.

Benzyl (1-phenylbut-3-en-1-yl)(tosylcarbamoyl)carbamate (16)

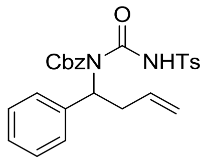

Chemical Formula: $C_{26}H_{26}N_2O_5S$
Exact Mass: 478.1562
Molecular Weight: 478.5600

To a cooled (− 78 °C) solution of benzyl (1-phenylbut-3-en-1-yl)carbamate (**12**, 380 mg, 1.35 mmol, 1.0 equiv) in dry diethyl ether (30 mL) was added a solution of *n*-BuLi (2.5 M in hexane, 600 µL, 1.49 mmol, 1.1 equiv) over 5 min using a syringe pump. After 10 min a solution of tosyl isocyanate (200 µL, 1.42 mmol, 1.1 equiv) in dry diethyl ether was added slowly over a period of 5 min. The reaction mixture was stirred at −78 °C for 1 h and quenched with aqueous saturated ammonium chloride (20 mL). The whole mixture was extracted with diethyl ether (3 x 20 mL). The combined organic phases were dried over $MgSO_4$ and concentrated under reduced pressure. The crude product was purified by column

chromatography on silica gel (100 mg) with ethyl acetate/hexane 1:9 as the eluent to afford the homoallylic amine (64.1 mg, 0.23 mmol, 17%) and the desired product as colorless solid (233 mg, 0.49 mmol, 36%, 43% brsm); R_f: 0.22 (ethyl acetate/hexane = 1:9); ^{1}H-NMR (300.13 MHz, $CDCl_3$): δ = 2.49 (s, 3H), 2.84 (t, *J* = 8.0 Hz, 2H), 4.86 (d, *J* = 17.0 Hz, 1H), 4.91 (d, *J* = 9.7 Hz, 1H), 5.00 (d, *J* = 11.9 Hz, 1H), 5.12 (d, *J* = 11.9 Hz, 1H), 5.60 (ddt, *J* = 7.3 Hz, 10.1 Hz, 17.4 Hz, 1H), 5.96 (t, *J* = 8.1 Hz, 1H), 7.08 (m, 4H), 7.24 (m, 3H), 7.36 (m, 5H), 8.01 (d, *J* = 8.2 Hz, 2H), 11.54 (bs, 1H); ^{13}C-NMR (75.47 MHz, $CDCl_3$): δ = 22.1, 35.5, 55.8, 70.0, 118.6, 127.4, 127.7, 128.6, 128.9, 129.1, 129.2, 129.4, 129.8, 134.1, 134.3, 136.2, 139.4, 145.2, 150.8, 156.2; HR-MS (ESI$^+$): calculated for $C_{26}H_{26}N_2O_5SNa^+$ $[M+Na]^+$: *m/z* = 501.2455, found: *m/z* = 501.2400.

1-((*tert*-Butyldimethylsilyl)oxy)propan-2-one (26)

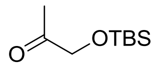

Chemical Formula: $C_9H_{20}O_2Si$
Exact Mass: 188.1233
Molecular Weight: 188.3394

tert-Butyldimethylsilyl chloride (2.48 g, 16.5 mmol, 1.1 equiv) was added to a stirred solution of 1-hydroxypropan-2-one (**25**, 1.11 g, 15.0 mmol, 1.0 equiv) and imidazole (1.43 g, 21.0 mmol, 1.4 equiv) in 20 mL dry dichloromethane at 0 °C. The mixture was stirred for 5 h at the same temperature, the solvent was evaporated under reduced pressure and the residue was extracted three times with diethyl ether and finally washed with water. The combined organic layer was dried over $MgSO_4$, filtered, concentrated and purified by column chromatography on silica gel (50 g) with ethyl acetate/hexane 1:40 as the eluent, which yielded the desired TBS-protected ketone as colorless liquid (2.48 g, 13.2 mmol, 88%). R_f: 0.18 (ethyl acetate/hexane = 1:40); ^{1}H-NMR (500.13 MHz, $CDCl_3$): δ = 0.09 (s, 6H), 0.93 (s, 9H), 2.17 (s, 3H), 4.15 (s, 2H); ^{13}C-NMR (125.77 MHz, $CDCl_3$): δ = -5.5, 18.3, 25.7, 69.6, 209.2; HR-MS (EI$^+$): calculated for $C_9H_{20}O_2Si^+$ $[M]^+$: *m/z* = 188.1227, found: *m/z* = 188.1242.

(S_S)-*N*-(1-((*tert*-Butyldimethylsilyl)oxy)propan-2-ylidene)-2-methylpropane-2-sulfinamide (29)

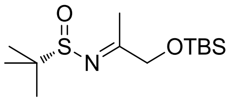

Chemical Formula: $C_{13}H_{29}NO_2SSi$
Exact Mass: 291.1688
Molecular Weight: 291.5254

1-((*tert*-Butyldimethylsilyl)oxy)propan-2-one (**26**, 1.88 g, 10.0 mmol, 1.0 equiv) was dissolved in 20 mL of dry THF. To this solution titanium(IV) isopropoxide (4.85 mL, 25.0 mmol, 2.6 equiv) and (*S*)-2-methyl-2-propanesulfinamide (**27**, 1.18 g, 9.70 mmol, 1.0 equiv) were added and the mixture was stirred at 70 °C for 19 h. After cooling to rt, the solution was poured into an equal volume of brine with vigorous stirring. The resulting suspension was filtered and washed with ethyl acetate. The aqueous layer was extracted three times with ethyl acetate, the combined organic layer was washed with brine, dried over $MgSO_4$, concentrated. Purification by column chromatography on silica gel (100 g) with ethyl acetate/hexane 1:9 as the eluent, yielded the desired *N*-sulfinyl ketimine as light yellow oil (1.87 g, 6.43 mmol, 66%); R_f: 0.25 (ethyl acetate/hexane = 1:9); $[\alpha]^{20}_D$ = +174.1 (c = 1.00, $CHCl_3$); ^{1}H-NMR (500.13 MHz, $CDCl_3$): δ = 0.09 (s, 6H), 0.91 (s, 9H), 1.24 (s, 9H), 2.33 (s, 3H), 4.23 (s, 2H); ^{13}C-NMR (125.77 MHz, $CDCl_3$): δ = -5.4, 18.2, 18.9, 22.2, 25.7, 56.6, 69.3, 184.2; HR-MS (FAB$^+$): calculated for $C_{13}H_{30}NO_2SSi^+$ $[M+H]^+$: *m/z* = 292.1716, found: *m/z* = 292.1783.

(R_S)-*N*-(1-((*tert*-butyldimethylsilyl)oxy)propan-2-ylidene)-2-methylpropane-2-sulfinamide (30)

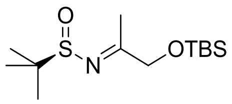

Chemical Formula: $C_{13}H_{29}NO_2SSi$
Exact Mass: 291.1688
Molecular Weight: 291.5254

1-((*tert*-Butyldimethylsilyl)oxy)propan-2-one (**26**, 1.51 g, 8.02 mmol, 1.0 equiv) was dissolved in 15 mL of dry THF. To this solution titanium(IV) isopropoxide (3.88 mL, 20.0 mmol, 2.5 equiv) and (*R*)-2-methyl-2-propanesulfinamide (**28**) (970 mg, 8.02 mmol, 1.0 equiv) were added and the mixture was stirred at 70 °C for 14 h. After cooling to rt, the solution was poured into an equal volume of brine with vigorous stirring. The resulting suspension was filtered and washed with ethyl acetate. The aqueous layer was extracted three times with ethyl acetate, the combined organic layer was washed with brine, dried over $MgSO_4$, concentrated. Purification by column chromatography on silica gel (100 g) with ethyl acetate/hexane 1:9 as eluent, yielded the desired imine as light yellow oil (1.28 g, 4.41 mmol, 55%); R_f: 0.1 (ethyl acetate/hexane = 1:9); $[\alpha]^{20}_D$ = -126.4 (c = 1.00, $CHCl_3$); ^{1}H NMR (300.13 MHz, $CDCl_3$): δ = 0.09 (s, 6H), 0.92 (s, 9H), 1.24 (s, 9H), 2.34 (s, 3H), 4.24 (s, 2H); ^{13}C NMR (75.47 MHz, $CDCl_3$): δ = -5.4, 18.2, 18.9, 22.2, 25.7, 56.6, 69.3, 184.2; HR-MS (FAB$^+$): calculated for $C_{13}H_{30}NO_2SSi^+$ $[M+H]^+$: *m/z* = 292.1716, found: *m/z* = 292.1772.

(R_S)-*N*-((*S*)-1-((*tert*-Butyldimethylsilyl)oxy)-2-methylpent-4-en-2-yl)-2-methylpropane-2-sulfinamide (33) and (R_S)-*N*-((*R*)-1-((*tert*-butyldimethylsilyl)oxy)-2-methylpent-4-en-2-yl)-2-methylpropane-2-sulfinamide (34)

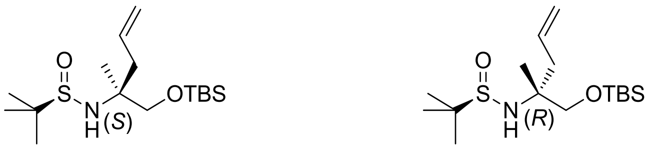

33 **34**

Chemical Formula: $C_{16}H_{35}NO_2SSi$
Exact Mass: 333.2158
Molecular Weight: 333.6051

In a flame-dried flask (R_S)-*N*-(1-((*tert*-butyldimethylsilyl)oxy)propan-2-ylidene)-2-methyl-propane-2-sulfinamide (**30**, 620 mg, 2.13 mmol, 1.0 equiv) was dissolved in 6.0 mL toluene and the solution was cooled to −78 °C. To this mixture allylmagnesium bromide (1.0 M in Et_2O, 3.2 mL, 3.20 mmol, 1.5 equiv) was slowly added and the reaction was stirred for 2 h at −78 °C. The reaction was quenched with a solution of saturated Na_2SO_4, warmed to rt, filtered, washed with ethyl acetate and finally purified by column chromatography on silica gel (50 g) with ethyl acetate/hexane 1:9 as eluent, which yielded the desired diasteromers (major diastereomer **33**, minor diastereomer **34**) as colorless oils (64%, dr = 1:1.4). Major diastereomer R_SS (264 mg, 0.79 mmol, 37%): R_f: 0.13 (ethyl acetate/ hexane = 1:9); $[\alpha]^{20}_D$ = -53.3 (c = 1.00, $CHCl_3$); ^{1}H-NMR (300.13 MHz, $CDCl_3$): δ = 0.06 (s, 6H), 0.91 (s, 9H), 1.19 (s, 12H), 2.48 (dd, *J* = 4.7 Hz, 7.4 Hz, 2H), 3.32 (d, *J* = 9.3 Hz, 1H), 3.49 (d, *J* = 9.3 Hz, 1H), 3.72 (bs, 1H), 5.11 (d, *J* = 10.4 Hz, 1H), 5.12 (d, *J* = 17.3 Hz, 1H), 5.80 (ddt, *J* = 7.7 Hz, 10.4 Hz, 17.8 Hz, 1H); ^{13}C-NMR (75.47 MHz, $CDCl_3$): δ = -5.5, 18.2, 22.1, 22.6, 25.8, 43.0, 55.5, 58.1, 69.2, 118.8, 133.8; HR-MS (FAB^+): calculated for $C_{16}H_{36}NO_2SSi^+$ $[M+H]^+$: *m/z* = 334.2231, found: *m/z* = 334.2226; minor diastereomer R_SR (192 mg, 0.58 mmol, 27%): R_f: 0.2 (ethyl acetate/hexane = 1:9); $[\alpha]^{20}_D$ = -40.7 (c = 1.00, $CHCl_3$); ^{1}H-NMR (300.13 MHz, $CDCl_3$): δ = 0.06 (s, 3H), 0.08 (s, 3H), 0.91 (s, 9H), 1.20 (s, 9H), 1.28 (s, 3H), 2.22 (dd, *J* = 8.0 Hz, 13.7 Hz, 1H), 2.37 (dd, *J* = 6.9 Hz, 13.7 Hz, 1H), 3.48 (d, *J* = 9.3 Hz, 1H), 3.52 (d, *J* = 9.6 Hz, 1H), 3.77 (bs, 1H), 5.09 (d, *J* = 17.8 Hz, 1H), 5.10 (d, *J* = 9.9 Hz, 1H), 5.78 (ddt, *J* = 7.7 Hz, 10.7 Hz, 17.3 Hz, 1H); ^{13}C-NMR (75.47 MHz, $CDCl_3$): δ = -5.5, 18.2, 22.3, 22.7, 25.8, 43.1, 55.6, 58.1, 69.9, 118.5, 133.6; HR-MS (FAB^+): calculated for $C_{16}H_{36}NO_2SSi^+$ $[M+H]^+$: *m/z* = 334.2231, found: *m/z* = 334.2223.

(*R*)-1-Hydroxy-2-methylpent-4-en-2-aminium chloride (35a)

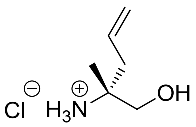

Chemical Formula: $C_6H_{14}ClNO$
Exact Mass: 151.0764
Molecular Weight: 151.6345

(S_S)-*N*-((*R*)-1-((*tert*-Butyldimethylsilyl)oxy)-2-methylpent-4-en-2-yl)-2-methylpropane-2-sulfinamide (**31**, 845 mg, 2.53 mmol, 1.0 equiv) was dissolved in 5.0 mL MeOH. Hydrogen chloride (4.0 M in dioxane, 6.5 mL, 6.50 mmol, 10 equiv) was added, the solution was stirred for 30 min at rt and concentrated in vacuo. To the residue was added diethylether (10 mL) and the mixture was stirred for 15 min at rt untill complete precipitation of the hydrochloride. The organic layer was removed and the hydrochloride was washed twice with diethylether. Finally, the solid was dried under high vacuum to afford the desired hydrochloride as colorless powder in quantitative yield (378 mg, 2.50 mmol, quant.). $[\alpha]^{20}_D$ = +2.1 (c = 1.00, MeOH); ^{1}H-NMR (300.13 MHz, MeOH-d^4): δ = 1.26 (s, 3H), 2.37 (dd, *J* = 7.5 Hz, 13.5 Hz, 1H), 2.44 (d, *J* = 11.5 Hz, 1H), 3.49 (d, *J* = 11.5 Hz, 1H), 3.56 (d, *J* = 11.5 Hz, 1H), 5.26 (dd, *J* = 1.9 Hz, 11.8 Hz, 1H), 5.26 (dd, *J* = 1.9 Hz, 9.7 Hz, 1H), 5.84 (ddt, *J* = 7.5 Hz, 9.7 Hz, 17.4 Hz, 1H); ^{13}C-NMR (75.47 MHz, MeOH-d^4): δ = 20.2, 40.8, 58.3, 66.2, 121.4, 131.9; HR-MS (EI^+): calculated for $C_6H_{14}NO^+$ [M-Cl]$^+$: *m/z* = 116.1069, found: *m/z* = 116.1063.

(*R*)-2-Amino-2-methylpent-4-en-1-ol (35)

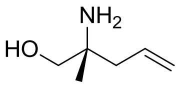

Chemical Formula: $C_6H_{13}NO$
Exact Mass: 115.0997
Molecular Weight: 115.1735

(*R*)-1-Hydroxy-2-methylpent-4-en-2-aminium chloride (**35a**, 360 mg, 2.38 mmol, 1.0 equiv) was dissolved in water (8.0 mL). To this solution, potassium hydroxide (2.85 g, 50.3 mmol, 20.0 equiv) was added and the mixture was stirred at rt for 1 h. The solution was extracted three times with dichloromethane, the combined organic layers were dried over $MgSO_4$ and the solvent was removed in vacuo to afford the desired amino alcohol as yellow oil (273 mg, 2.37 mmol, 99%). $[\alpha]^{20}_D$ = +0.20 (c = 1.00, $CHCl_3$); ^{1}H-NMR (300.13 MHz, $CDCl_3$): δ = 1.05 (s, 3H), 1.94 (bs, 3H), 2.14 (d, *J* = 7.4 Hz, 2H), 3.29 (d, *J* = 10.4 Hz, 1H), 3.34 (d, *J* = 10.7 Hz, 1H), 5.11 (d, *J* = 16.7 Hz, 1H), 5.12 (d, *J* = 10.2 Hz, 1H), 5.83 (ddt, *J* = 7.4 Hz, 10.4 Hz, 17.8 Hz, 1H); ^{13}C-NMR (75.47 MHz, $CDCl_3$): δ = 24.6, 44.4, 52.7, 70.1, 118.6, 133.8; HR-MS (EI^+): calculated for $C_6H_{14}NO^+$ [M+H]$^+$: *m/z* = 116.1069, found: *m/z* = 116.1048.

(*R*)-1-((*tert*-Butyldimethylsilyl)oxy)-2-methylpent-4-en-2-amine (36)

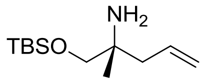

Chemical Formula: $C_{12}H_{27}NOSi$
Exact Mass: 229.1862
Molecular Weight: 229.4344

To an ice-cooled (0 °C) solution of (*R*)-2-amino-2-methylpent-4-en-1-ol (**35**, 225 mg, 2.22 mmol, 1.0 equiv), triethylamine (0.4 mL, 2.66 mmol, 1.2 equiv) and 4-(dimethylamino)pyridine (13.4 mg, 0.11 mmol, 5 mol %) in dichloromethane (2.5 mL) was added *tert*-butyldimethylsilyl chloride (368 mg, 2.44 mmol, 1.1 equiv). The mixture was allowed to warm to rt and was stirred overnight. The solution was washed with water and saturated NH_4Cl solution. The organic layer was dried over $MgSO_4$ and concentrated. The residue was purified by flash column chromatography on silica gel (30 g) using 5% MeOH in dichloromethane as eluent to afford pure (*R*)-1-((*tert*-butyldimethylsilyl)oxy)-2-methylpent-4-en-2-amine as pale yellow oil (503 mg, 2.20 mmol, 99%). R_f: 0.63 (MeOH/dichloromethane = 1:9); $[\alpha]^{20}_{D}$ = +2.90 (c = 1.00, $CHCl_3$); ^{1}H-NMR (300.13 MHz, $CDCl_3$): δ = 0.05 (s, 3H), 0.91 (s, 9H), 1.02 (s, 3H), 1.17 (bs, 2H), 2.15 (d, *J* = 7.4 Hz, 2H), 3.31 (d, *J* = 9.6 Hz, 1H), 3.36 (d, *J* = 9.3 Hz, 1H), 5.08 (d, *J* = 17.6 Hz, 1H), 5.09 (d, *J* = 17.6 Hz, 1H), 5.83 (ddt, *J* = 7.7 Hz, 11.3 Hz, 15.4 Hz, 1H); ^{13}C-NMR (75.47 MHz, $CDCl_3$): δ = -5.5, 18.2, 24.3, 25.9, 43.9, 52.9, 71.1, 118.1, 134.3; HR-MS (ESI$^+$): calculated for $C_{12}H_{28}NOSi^+$ $[M+H]^+$: *m/z* = 230.1935, found: *m/z* = 230.1935; $[M+Na]^+$ calculated: *m/z* = 252.1754, found: *m/z* = 252.1754.

Mosher amide analysis of (R)-1-((tert-butyldimethylsilyl)oxy)-2-methyl-pent-4-en-2-amine (36)

(*R*)-*N*-((*R*)-1-((*tert*-Butyldimethylsilyl)oxy)-2-methylpent-4-en-2-yl)-3,3,3-trifluoro-2-methoxy-2-phenylpropanamide (36a)

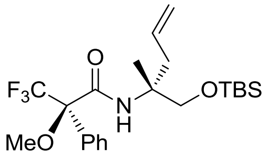

Chemical Formula: $C_{22}H_{34}F_3NO_3Si$
Exact Mass: 445.2260
Molecular Weight: 445.5910

(*R*)-1-((*tert*-Butyldimethylsilyl)oxy)-2-methylpent-4-en-2-amine (**36**, 22.1 mg, 96.4 µmol, 1.2 equiv) was dissolved in 0.5 mL dichloromethane in a micro-reaction vial. (*S*)-α-Methoxy-α-(trifluoro-methyl) phenylacetyl chloride (15.0 µL, 80.4 µmol, 1.0 equiv) and *N*,*N*-diisopropylethylamine (16.8 µL, 96.4 µmol, 1.2 equiv) were added. The mixture was stirred at rt for 15 min and diluted in 10 mL

dichloromethane. The solution was extracted with 3 x 15 mL of 5% HCl, washed with deionized water and dried over $MgSO_4$. A micro-flash column was prepared by packing a Pasteur pipette with glass wool and SiO_2. The residue was eluted using a mixture of hexane/ethyl acetate 3:1 to afford the desired Mosher amide (42.5 mg, 95.5 µmol, 99%) in quantitative yield for ^{1}H NMR analysis. R_f: 0.85 (ethyl acetate/ hexane = 1:3); $[\alpha]^{20}_D$ = -5.30 (c = 1.00, $CHCl_3$); ^{1}H-NMR (300.13 MHz, $CDCl_3$): δ = 0.06 (s, 6H), 0.90 (s, 9H), 1.36 (s, 3H), 2.53 (ddt, *J* = 7.6 Hz, 13.8 Hz, 21.6 Hz, 2H), 3.41 (s, 3H), 3.48 (d, *J* = 9.8 Hz, 1H), 3.64 (d, *J* = 9.8 Hz. 1H), 5.07 (d, *J* = 13.2 Hz, 2H), 5.72 (ddt, *J* = 7.6 Hz, 8.8 Hz, 16.4 Hz, 1H), 7.01 (bs, 1H), 7.39 (m, 3H), 7.56 (m, 2H); HR-MS (ESI$^+$): calculated for $C_{22}H_{35}F_3NO_3Si^+$ $[M+H]^+$: *m/z* = 446.2334, found: *m/z* = 446.2332; $C_{22}H_{34}F_3NO_3SiNa^+$ $[M+Na]^+$: *m/z* = 468.2153, found: *m/z* = 468.2152.

(*S*)-*N*-((*R*)-1-((*tert*-Butyldimethylsilyl)oxy)-2-methylpent-4-en-2-yl)-3,3,3-trifluoro-2-methoxy-2-phenylpropanamide (36b)

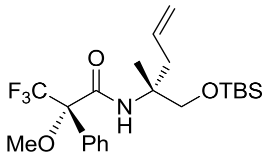

Chemical Formula: $C_{22}H_{34}F_3NO_3Si$
Exact Mass: 445.2260
Molecular Weight: 445.5910

(*R*)-1-((*tert*-Butyldimethylsilyl)oxy)-2-methylpent-4-en-2-amine (**36**, 22.1 mg, 96.4 µmol, 1.2 equiv) was dissolved in 0.5 mL dichloromethane in a micro-reaction vial. (*R*)-α-Methoxy-α-(trifluoromethyl) phenylacetyl chloride (15.0 µL, 80.4 µmol, 1.0 equiv) and *N*,*N*-diisopropylethylamine (16.8 µL, 96.4 µmol, 1.2 equiv) were added. The mixture was stirred at rt for 15 min and diluted in 10 mL dichloromethane. The solution was extracted with 3 x 15 mL of 5% HCl, washed with deionized water and dried over $MgSO_4$. A micro-flash column was prepared by packing a Pasteur pipette with glass wool and SiO_2. The residue was eluted using a mixture of hexane/ethyl acetate 3:1 to afford the desired Mosher amide (42.5 mg, 95.5 µmol, 99%) for ^{1}H NMR analysis. R_f: 0.81 (ethyl acetate/ hexane = 1:3); $[\alpha]^{20}_D$ = +1.00 (c = 1.00, $CHCl_3$); ^{1}H-NMR (300.13 MHz, $CDCl_3$): δ = 0.06 (s, 6H), 0.89 (s, 9H), 1.33 (s, 3H), 2.57 (d, *J* = 7.3 Hz, 2H), 3.42 (s, 3H), 3.43 (d, *J* = 9.6 Hz, 1H), 3.66 (d, *J* = 9.6 Hz, 1H), 5.09 (d, *J* = 11.3 Hz, 1H), 5.10 (d, *J* = 15.7 Hz, 1H), 5.76 (ddt, *J* = 7.6 Hz, 8.8 Hz, 16.4 Hz, 1H), 7.01 (bs, 1H), 7.39 (m, 3H), 7.55 (m, 2H); HR-MS (ESI$^+$): calculated for $C_{22}H_{35}F_3NO_3Si^+$ $[M+H]^+$: *m/z* = 446.2332, found: *m/z* = 446.2334; $C_{22}H_{34}F_3NO_3SiNa^+$ $[M+Na]^+$: *m/z* = 468.2152, found: *m/z* = 468.2154.

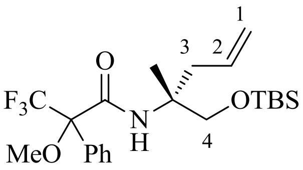

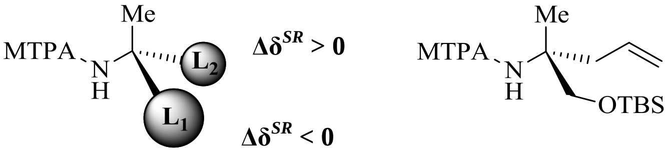

proton	δ (*S*)-Mosher amide (36b) [ppm]	δ (*R*)-Mosher amide (36a) [ppm]	$\Delta\delta^{SR}$
1	5.10	5.07	+0.03
2	5.76	5.72	+0.04
3	2.57	2.53	+0.04
4	3.55	3.56	-0.01

(*R*)-Benzyl (1-((*tert*-butyldimethylsilyl)oxy)-2-methylpent-4-en-2-yl)carbamate (37)

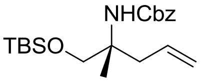

Chemical Formula: $C_{20}H_{33}NO_3Si$
Exact Mass: 363.2230
Molecular Weight: 363.5664

To a stirred solution of (*R*)-1-((*tert*-butyldimethylsilyl)oxy)-2-methylpent-4-en-2-amine (**36**, 217 mg, 0.95 mmol, 1.0 equiv) in dichloromethane was added potassium carbonate (98.1 mg, 0.71 mmol, 75 mol %) and 0.5 mL of water. The mixture was warmed to 32 °C, and benzyl chloroformate (0.18 mL, 1.24 mmol, 1.5 equiv) was added dropwise while maintaining the temperature at 30–35 °C. The reaction mixture was stirred at 33 °C for 4 h, then aqueous NH_4Cl (0.5 mL) was added and stirring continued for further 15 min. After cooling to rt the phases were separated and the aqueous layer was washed with dichloromethane. The combined organic layers were washed with water, and the water was back-extracted with dichloromethane. Finally the combined organic phases were dried over $MgSO_4$, concentrated in vacuo and purified by flash column chromatography on silica gel (30 g) using ethyl acetate/hexane 1:40 as the eluent to afford pure carbamate in quantitative yield (344 mg, 0.95 mmol, quant.). $[\alpha]^{20}_D$ = +3.50 (c = 1.00, $CHCl_3$); R_f: 0.23 (ethyl acetate/hexane = 1:40); ^{1}H-NMR (300.13 MHz,

$CDCl_3$): δ = 0.05 (s, 6H), 0.09 (s, 9H), 1.29 (s, 3H), 2.39 (dd, *J* = 7.4 Hz, 13.7 Hz, 1H), 2.52 (dd, *J* = 7.1 Hz, 13.5 Hz, 1H), 3.53 (d, *J* = 9.9 Hz, 1H), 3.61 (d, *J* = 9.6 Hz, 1H), 4.92 (bs, 1H), 5.09 (m, 4H), 5.79 (ddt, *J* = 7.4 Hz, 12.6 Hz, 14.8 Hz, 1H), 7.36 (m, 5H); ^{13}C-NMR (75.47 MHz, $CDCl_3$): δ = -5.5, 18.2, 21.3, 25.8, 40.5, 55.9, 66.1, 67.3, 118.5, 127.9, 128.0, 128.5, 133.6, 136.8, 155.0 ; HR-MS (ESI$^+$): calculated for $C_{20}H_{34}NO_3Si^+$ [M+H]$^+$: *m/z* = 364.2302, found: *m/z* = 364.2303; $C_{20}H_{33}NO_3SiNa^+$ [M+Na]$^+$: *m/z* = 386.2121, found: *m/z* = 386.2123.

(*R*,*E*)-Benzyl (9,12,12,13,13-pentamethyl-3-oxo-2,4,11-trioxa-12-silatetradec-6-en-9-yl)-carbamate (38)

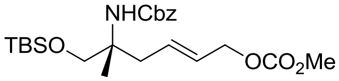

Chemical Formula: $C_{23}H_{37}NO_6Si$
Exact Mass: 451.2390
Molecular Weight: 451.6285

To a stirred solution of (*R*)-benzyl (1-((*tert*-butyldimethylsilyl)oxy)-2-methylpent-4-en-2-yl)carbamate (**37**, 665 mg, 1.83 mmol, 1.0 equiv) and (*Z*)-(but-2-ene-1,4-diyl)dimethyl dicarbonate (**17**, 934 mg, 4.58 mmol, 2.5 equiv) in dry toluene (5 mL) was added Grubbs catalyst 2nd generation (**21**, 155 mg, 0.18 mmol, 10 mol %). The reaction mixture was stirred for 15 h at 80 °C under an argon atmosphere and concentrated in vacuo. Purification by column chromatography on silica gel (100 g) with ethyl acetate/hexane 1:20 as the eluent yielded the desired isomers (major isomer *trans*-**38**, minor isomer *cis*-**38b**) as brown oils (71%, *cis*:*trans* = 1:5.3). Major isomer *trans*-**38** (489 mg, 1.08 mmol, 59%): R_f: 0.11 (ethyl acetate/ hexane = 1:20); ^{1}H-NMR (300.13 MHz, $CDCl_3$): δ = 0.04 (s, 6H), 0.89 (s, 9H), 1.27 (s, 3H), 2.42 (dd, *J* = 7.1 Hz, 13.7 Hz, 1H), 2.54 (dd, *J* = 7.1 Hz, 13.7 Hz, 1H), 3.50 (d, *J* = 9.6 Hz, 1H), 3.58 (d, *J* = 9.9 Hz, 1H), 3.77 (s, 3H), 4.56 (d, *J* = 6.0 Hz, 2H), 4.89 (s, 1H), 5.05 (s, 2H), 5.64 (dt, *J* = 6.3 Hz, 15.4 Hz, 1H), 5.78 (dt, *J* = 7.7 Hz, 15.4 Hz, 1H), 7.36 (m, 5H); ^{13}C-NMR (75.47 MHz, $CDCl_3$): δ = -5.6, 18.2, 21.5, 25.8, 38.6, 54.7, 56.0, 66.1, 67.4, 68.3, 127.0, 128.0, 128.1, 128.5, 131.6, 136.7, 155.6; HR-MS (ESI$^+$): calculated for $C_{23}H_{38}NO_6Si^+$ [M+H]$^+$: *m/z* = 452.2462, found: *m/z* = 452.2466; $C_{23}H_{37}NO_6SiNa^+$ [M+Na]$^+$: *m/z* = 474.2282, found: *m/z* = 474.2285; $C_{23}H_{37}NO_6SiK^+$ [M+K]$^+$: *m/z* = 490.2021, found: *m/z* = 490.2024; minor isomer *cis*-**360** (93.0 mg, 0.21 mmol, 12%): R_f: 0.12 (ethyl acetate/ hexane = 1:20); ^{1}H-NMR (300.13 MHz, $CDCl_3$): δ = 0.05 (s, 6H), 0.09 (s, 9H), 1.30 (s, 3H), 2.47 (dd, *J* = 5.8 Hz, 14.6 Hz, 1H), 2.62 (dd, *J* = 6.0 Hz, 14.6 Hz, 1H), 3.53 (d, *J* = 9.6 Hz, 1H), 3.63 (d, *J* = 9.6 Hz, 1H), 3.74 (s, 3H), 4.68 (m, 2H), 5.01 (s, 1H), 5.05 (s, 2H), 5.70 (dt, *J* = 5.5 Hz, 11.8 Hz, 2H), 7.35 (m, 5H); ^{13}C-NMR (75.47 MHz, $CDCl_3$): δ = -5.5, 18.2, 21.5, 25.8, 33.7, 54.7, 56.2, 63.5, 66.1, 67.4, 125.9, 128.0, 128.1, 128.5, 130.3, 136.7, 155.0, 155.7; HR-MS (ESI$^+$): calculated for $C_{23}H_{38}NO_6Si^+$ [M+H]$^+$: *m/z* = 452.2462, found: *m/z* = 452.2465.

(4*R*,6*S*)-4-((*tert*- Butyldimethylsilyloxy)methyl)-4-methyl-6-vinyltetrahydropyrimidin-2(1*H*)-one (44)

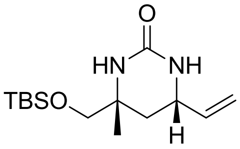

Chemical Formula: $C_{14}H_{28}N_2O_2Si$
Exact Mass: 284.1920
Molecular Weight: 284.4698

SmI_2 (0.1 M in THF, 7.5 mL, 754 µmol, 6 equiv) was added to a solution of (4*R*,6*S*)-4-((*tert*-butyldimethylsilyloxy)methyl)-4-methyl-1-tosyl-6-vinyltetrahydropyrimidin-2(1*H*)-one (**42**, 55.1 mg, 126 µmol) in dry THF (3.4 mL) at 0 °C and the reaction mixture was stirred at rt for 5 h. The reaction was quenched by the addition of a saturated $NaHCO_3$ solution (15 mL) and the mixture extracted with ethyl acetate until the color of the aqueous layer turned white. Afterwards, the combined organic extracts were washed with brine and dried over $MgSO_4$. The solvent was removed under reduced pressure and the residue purified by column chromatography on silica gel (5% MeOH in CH_2Cl_2) to yield the pyrimidine (36.1 mg, 127 µmol, quant.) as a red solid. R_f: 0.32 (5% MeOH in dichloromethane); $[\alpha]_D^{20}$ = +10.9 (c = 0.5, $CHCl_3$); ^{1}H-NMR (400.13 MHz, $CDCl_3$): δ [ppm] = 0.06 (s, 6H), 0.90 (s, 9H), 1.26 (s, 3H), 1.44 (dd, *J* = 12.8, 11.6 Hz, 1H), 1.65 (dd, *J* = 12.8, 3.9 Hz, 1H), 3.40 (d, *J* = 9.4 Hz, 1H), 3.45 (d, *J* = 9.4 Hz, 1 H), 4.05 (ddd, *J* = 11.6, 7.1, 3.9 Hz, 1H), 4.94 (br. s, 1H), 5.11 (br. s, 1H), 5.17 (d, *J* = 10.2 Hz, 1 H), 5.30 (d, *J* = 17.1 Hz, 1H), 5.77 (ddd, *J* = 17.1, 10.2, 7.1 Hz, 1 H); ^{13}C-NMR (100.62 MHz, $CDCl_3$): δ [ppm] = -5.4, -5.4, 18.4, 24.5, 26.0, 36.7, 50.8, 53.9, 71.3, 116.9, 138.2, 156.1; HR-MS (ESI^+): calculated for $C_{14}H_{29}N_2O_2Si^+$ $[M+H]^+$: *m/z* = 285.1993, found: *m/z* = 285.1989; Mp: 105 °C.

(4*R*,6*S*)-4-((*tert*-Butyldimethylsilyloxy)methyl)-6-(hydroxymethyl)-4-methyltetrahydropyrimidin-2(1*H*)-one (3)

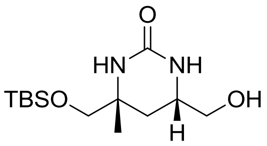

Chemical Formula: $C_{13}H_{28}N_2O_3Si$
Exact Mass: 288.1869
Molecular Weight: 288.4585

A solution of (4*R*,6S)-4-((*tert*-butyldimethylsilyloxy)methyl)-4-methyl-6-vinyltetrahydropyrimidin-2(1*H*)-one (**44**, 49.3 mg, 173 µmol), 2,6-lutidine (40 µl, 347 µmol, 2 equiv) and OsO_4 (2.5 wt % in *tert*-butanol, 43 µL, 3.47 µmol, 2 mol %) in dioxane/water (1.7 mL, 3:1) was stirred at rt for 15 min. $NaIO_4$ (148 mg, 693 µmol, 4 equiv) was added and stirring was continued for 5 h. Afterwards, the organic layer was separated and the aqueous layer was extracted three times with dichloromethane. The combined organic extracts were washed with brine (1 x), dried over $MgSO_4$ and concentrated. The residue was dissolved in methanol (2 mL) and $NaBH_4$ (50.0 mg, 1.32 mmol, 7.6 equiv) was added in portions to the stirred solution at 0 °C. The reaction mixture was stirred at 0 °C for additional 2 h. Then the solution was allowed to warm to rt and stirring was continued for 18 h. The reaction was quenched by addition of a saturated $NaHCO_3$ solution (10 mL). The aqueous layer was extracted four times with dichloromethane and the combined organic extracts were dried over $MgSO_4$. The solvent was removed under reduced pressure and purification by column chromatography on silica gel (ethyl acetate/cyclohexane, 95:5 + 3% $iPrNH_2$) yielded the alcohol (32.6 mg, 113 µmol, 65%) as a colorless solid. R_f: 0.20 (5% MeOH in dichloromethane) $[\alpha]_D^{20}$ = +21.2 (c = 0.5, $CHCl_3$); Mp: 134 C. ^{1}H-NMR (400.13 MHz, $CDCl_3$): δ [ppm] = 0.06 (s, 6H), 0.90 (s, 9H), 1.25 (s, 3H), 1.34 (dd, *J* = 12.6, 12.2 Hz, 1H), 1.50 (dd, *J* = 12.6, 3.2 Hz, 1H), 3.14 (br. s, 1H), 3.49-3.39 (m, 3H), 3.73-3.64 (m, 2H), 5.09 (br. s, 1H), 6.49 (br. s, 1H); ^{13}C-NMR (75.47 MHz, $CDCl_3$): δ [ppm] = -5.4, -5.4, 18.4, 24.3, 26.0, 32.1, 49.7, 53.6, 66.0, 71.4, 157.1; HR-MS (ESI$^+$): calculated for $C_{13}H_{28}N_2NaO_3Si^+$ $[M+Na]^+$: *m/z* = 311.1761, found: *m/z* = 311.1753.

(4*R*,6*R*)-4-((*tert*-Butyldimethylsilyloxy)methyl)-4-methyl-6-vinyltetrahydropyrimidin-2(1*H*)-one (45)

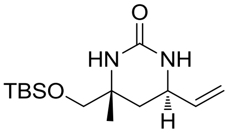

Chemical Formula: $C_{14}H_{28}N_2O_2Si$
Exact Mass: 284.1920
Molecular Weight: 284.4698

A stirred solution of (4*R*,6*R*)-4-((*tert*-butyldimethylsilyloxy)methyl)-4-methyl-1-tosyl-6-vinyl-tetrahydropyrimidin-2(1*H*)-one (**43**, 615 mg, 1.40 mmol) in dry THF (18 mL) was cooled to 0 °C and SmI_2 (0.1 M solution in THF, 84 mL, 8.41 mmol, 6.0 equiv) was added. The blue reaction mixture was stirred for 10 min at 0 °C and for another 15 h at rt. After quenching the reaction with saturated sodium bicarbonate solution the aqueous phase was extracted four times with ethyl acetate. The combined organic layers were washed with brine, dried over $MgSO_4$, filtered and the volatiles were removed under reduced pressure. The crude product was purified by column chromatography (silica gel, dichloromethane/methanol 95:5) to yield the desired compound as a red oil (398 mg, 1.40 mmol, quant.). R_f: 0.34 (dichloromethane/methanol, 95:5); Mp: 104 °C; $[\alpha]_D^{20}$ = –49.6 (c = 0.5, $CHCl_3$); ^{1}H NMR (300.13 MHz, $CDCl_3$): δ [ppm] = 0.05 (s, 6H), 0.89 (s, 9H), 1.21 (s, 3H), 1.38 (dd, *J* = 13.4, 11.5 Hz, 1H), 2.03 (dd, *J* = 13.4, 4.1 Hz, 1H), 3.36 (d, *J* = 9.7 Hz, 1H), 3.51 (d, *J* = 9.7 Hz, 1H), 3.95 (ddd, *J* = 11.5, 7.0, 4.1 Hz, 1H), 5.03 (br. s., 1H), 5.29-5.07 (m, 3H), 5.74 (ddd, *J* = 17.1, 10.1, 7.0 Hz, 1H); ^{13}C NMR (75.47 MHz, $CDCl_3$): δ [ppm] = -5.4, -5.3, 18.3, 25.9, 25.9, 36.1, 51.2, 53.8, 69.1, 116.6, 138.4, 156.5; HR-MS (ESI+): calculated for $C_{14}H_{28}N_2O_2SiNa^+$ $[M+Na]^+$: *m/z* = 307.1812, found: *m/z* = 307.1800.

(4*R*,6*R*)-4-((*tert*-Butyldimethylsilyloxy)methyl)-6-(hydroxymethyl)-4-methyltetrahydropyrimidin-2(1*H*)-one (4)

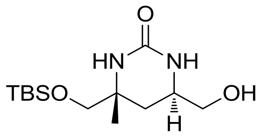

Chemical Formula: C13H28N2O3Si$C_{13}H_{28}N_2O_3Si$
Exact Mass: 288.1869288,18692
Molecular Weight: 288.4585288,45852

To a solution of (4*R*,6R)-4-((*tert*-butyldimethylsilyloxy)methyl)-4-methyl-6-vinyltetrahydropyrimidin-2(1*H*)-one (**45**, 372 mg, 1.31 mmol) in dioxane/water (13 mL, 3:1), 2,6-lutidine (0.3 mL, 2.61 mmol, 2.0 equiv) and OsO_4 (2.5 wt % in *tert*-butanol, 0.3 mL, 26.1 µmol, 2 mol %) were added. The reaction mixture was stirred for 10 min at rt before $NaIO_4$ (1.12 g, 5.26 mmol, 4.0 equiv) was added. After 15 h of stirring the aqueous layer was extracted four times with dichloromethane. The combined organic layers were washed with brine, dried over $MgSO_4$, filtered and concentrated in vacuo. The residue was dissolved in methanol (17 mL), cooled to 0 °C and $NaBH_4$ (595 mg, 15.7 mmol, 12 equiv) was added. The resulting mixture was slowly warmed to rt, stirred at this temperature for 16 h and quenched by addition of saturated $NaHCO_3$ solution. The aqueous layer was extracted four times with dichloromethane and the combined organic layers were dried over $MgSO_4$, filtered and the solvent was removed under reduced pressure. Purification by column chromatography (silica gel, cyclohexane/ethyl acetate 1:9 and 10% isopropylamine) yielded the alcohol as a white solid (243 mg, 812 µmol, 62%). R_f: 0.23 (dichlormethane/methanol, 95:5). Mp: 137 °C; $[\alpha]_D^{20}$ = –47.1 (c = 0.5, $CHCl_3$); ^{1}H NMR (400.13 MHz, $CDCl_3$):δ [ppm] = 0.04 (s, 3H), 0.04 (s,

3H), 0.88 (s, 9H), 1.20 (s, 3H), 1.27 (dd, *J* = 13.1, 12.5 Hz, 1H), 1.89 (dd, *J* = 13.1, 3.6 Hz, 1H), 3.33 (d, *J* = 9.8 Hz, 1H), 3.43 (dd, *J* = 11.2, 7.8 Hz, 1H), 3.50 (d, *J* = 9.8 Hz, 1H), 3.59-3.52 (m, 1H), 3.66 (dd, *J* = 11.2, 2.8 Hz, 1H), 4.97 (brs, 1H), 6.54 (brs, 1H); ^{13}C NMR (100.62 MHz, $CDCl_3$): δ [ppm] = -5.4, -5.4, 18.3, 25.9, 26.0, 31.4, 50.0, 53.6, 66.1, 68.8, 157.2; HR-MS (ESI+): calculated for $C_{13}H_{28}N_2O_3SiNa^+$ $[M+Na]^+$: *m/z* = 311.1761, found: *m/z* = 311.1755.

3. NMR Spectra

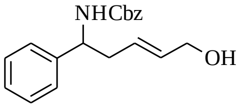

14

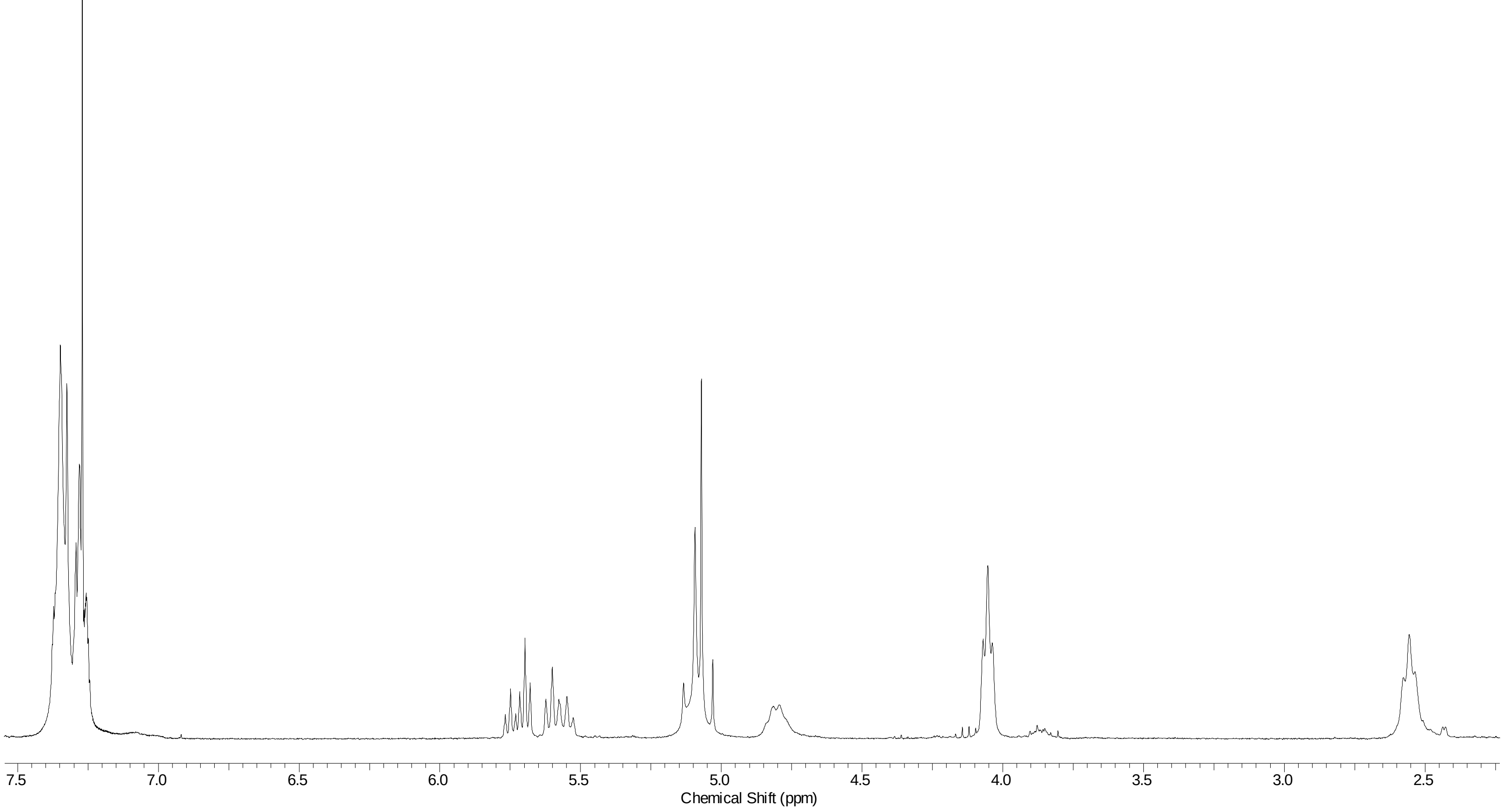

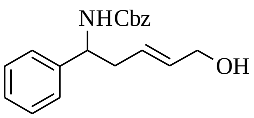
NHCbz
OH

14

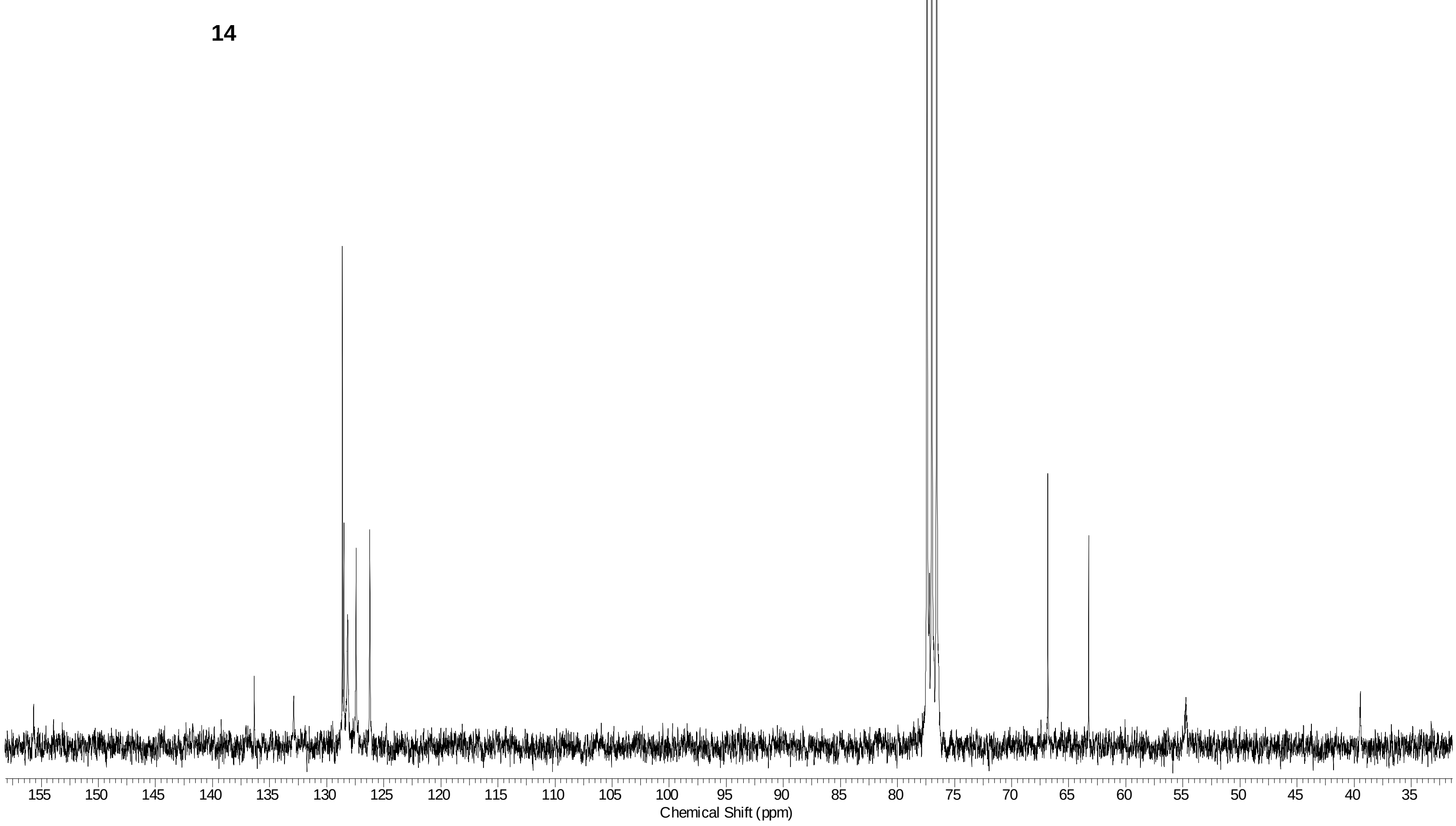
155 150 145 140 135 130 125 120 115 110 105 100 95 90 85 80 75 70 65 60 55 50 45 40 35
Chemical Shift (ppm)

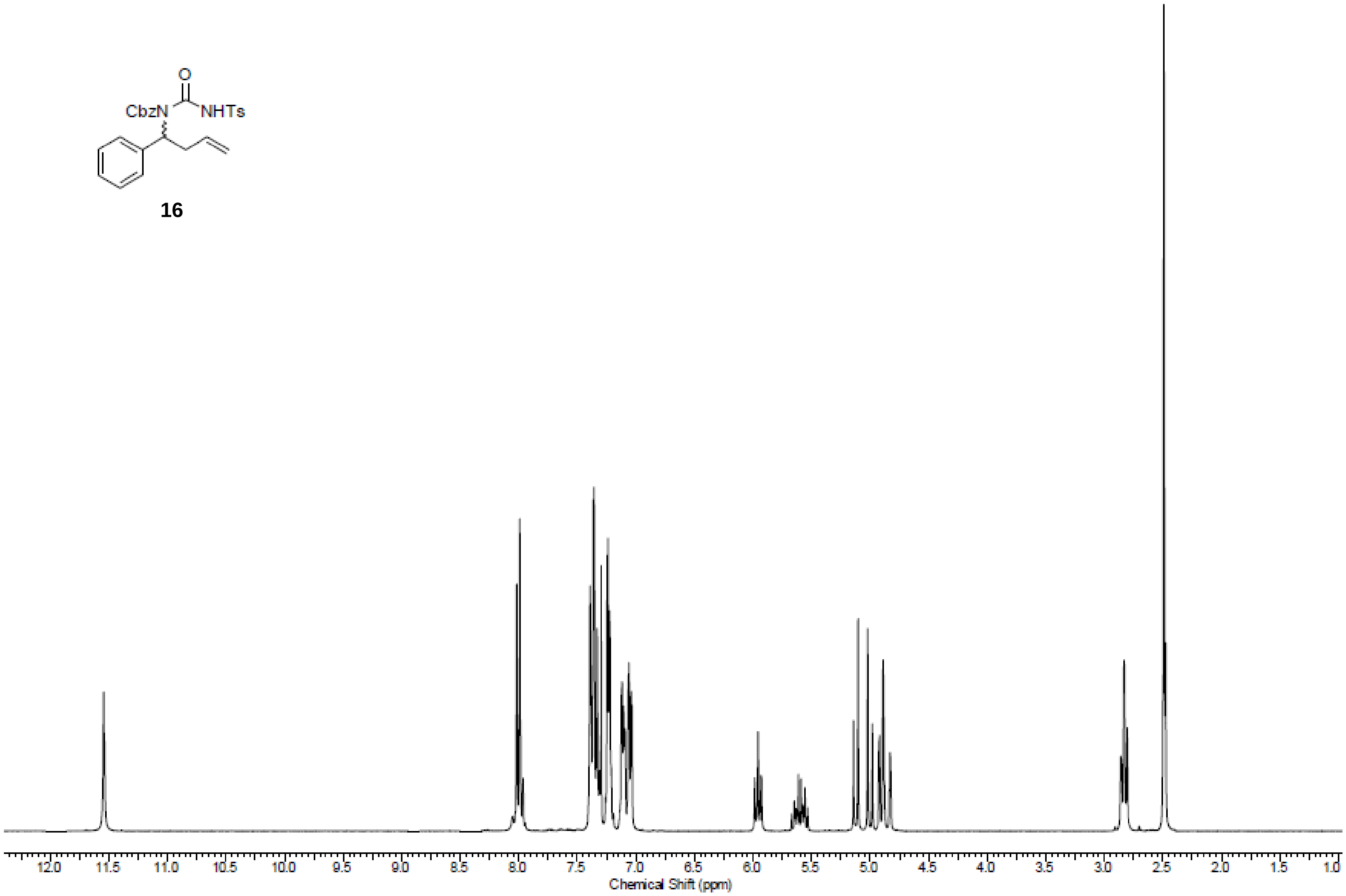
O
CbzN
NHTs
16
12.0
11.5
11.0
10.5
10.0
9.5
9.0
8.5
8.0
7.5
7.0
6.5
6.0
5.5
5.0
4.5
4.0
3.5
3.0
2.5
2.0
1.5
1.0
Chemical Shift (ppm)

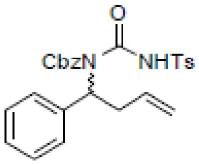
O
CbzN
NHTs

16

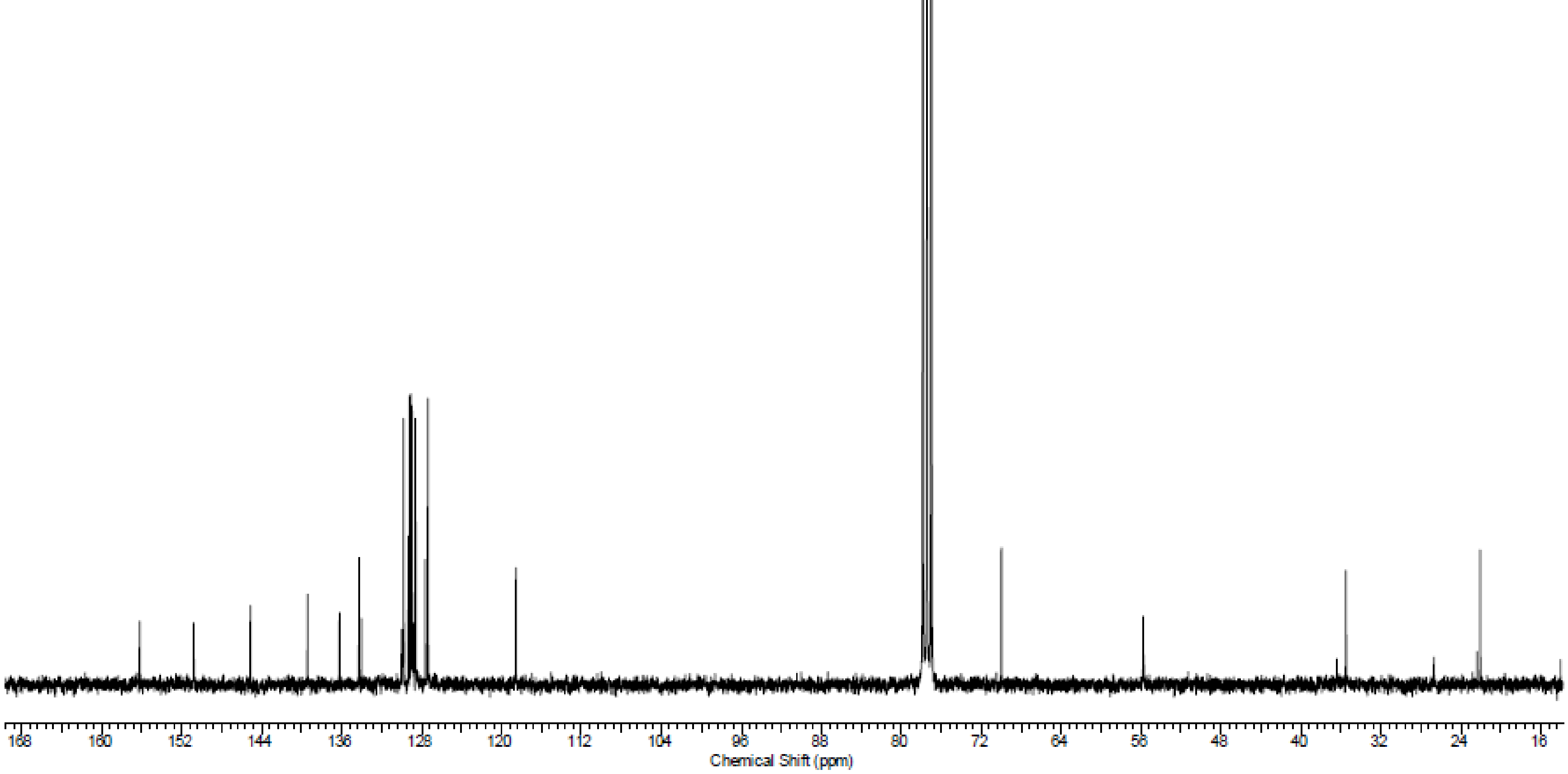
168
160
152
144
136
128
120
112
104
96
88
80
72
64
56
48
40
32
24
16
Chemical Shift (ppm)

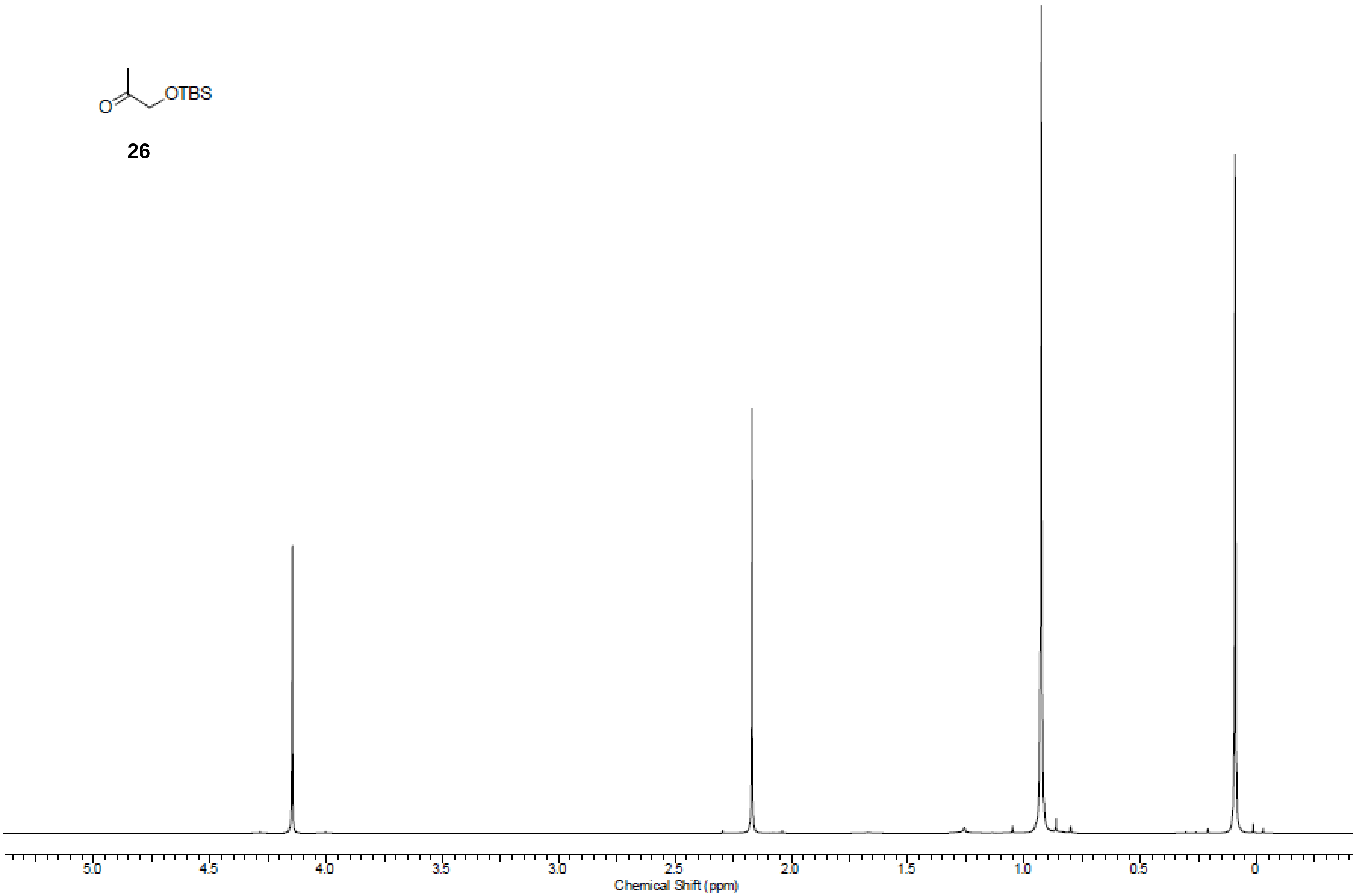
O
OTBS
26
5.0
4.5
4.0
3.5
3.0
2.5
2.0
1.5
1.0
0.5
0
Chemical Shift (ppm)

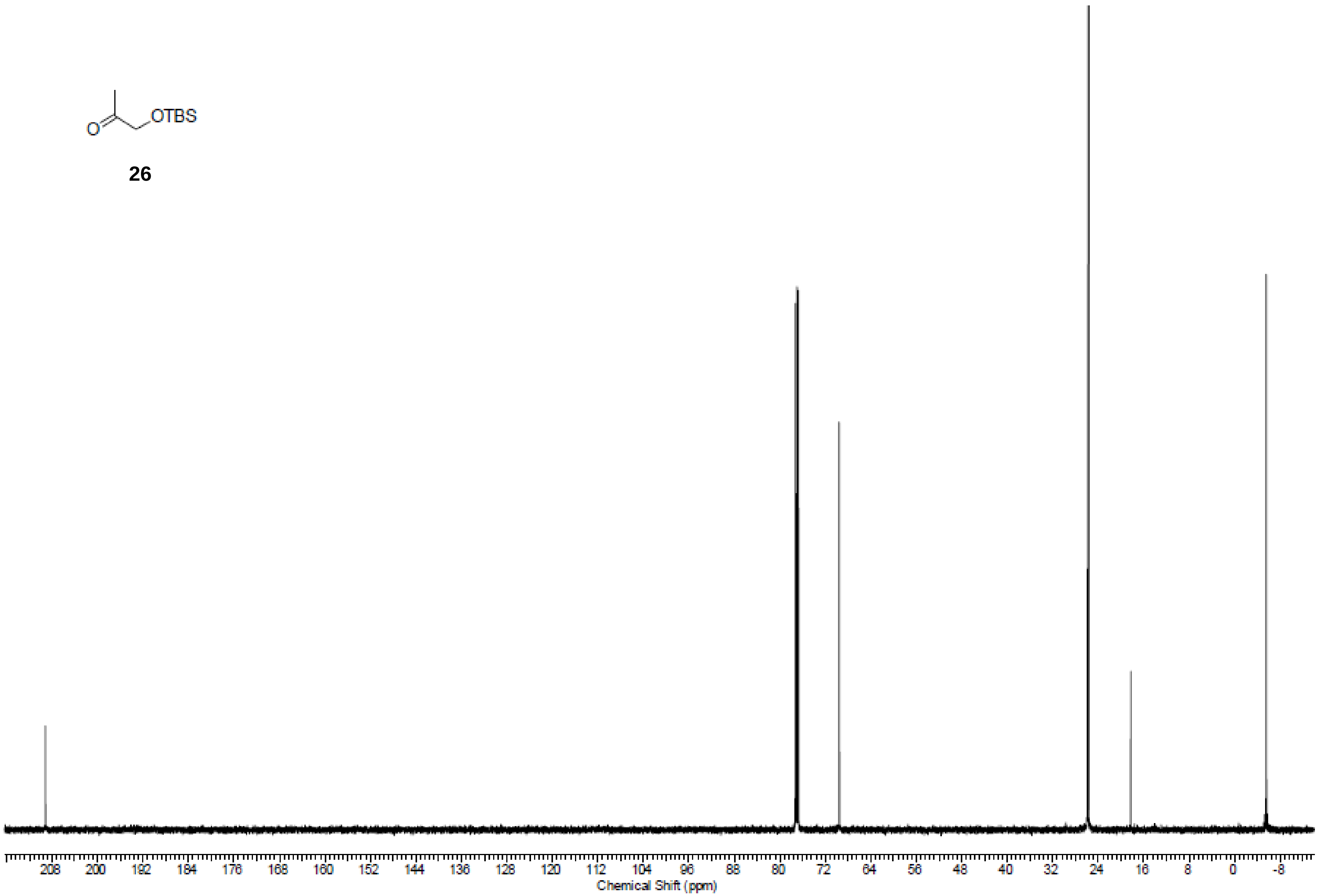
OTBS
O
26
208 200 192 184 176 168 160 152 144 136 128 120 112 104 96 88 80 72 64 56 48 40 32 24 16 8 0 -8
Chemical Shift (ppm)

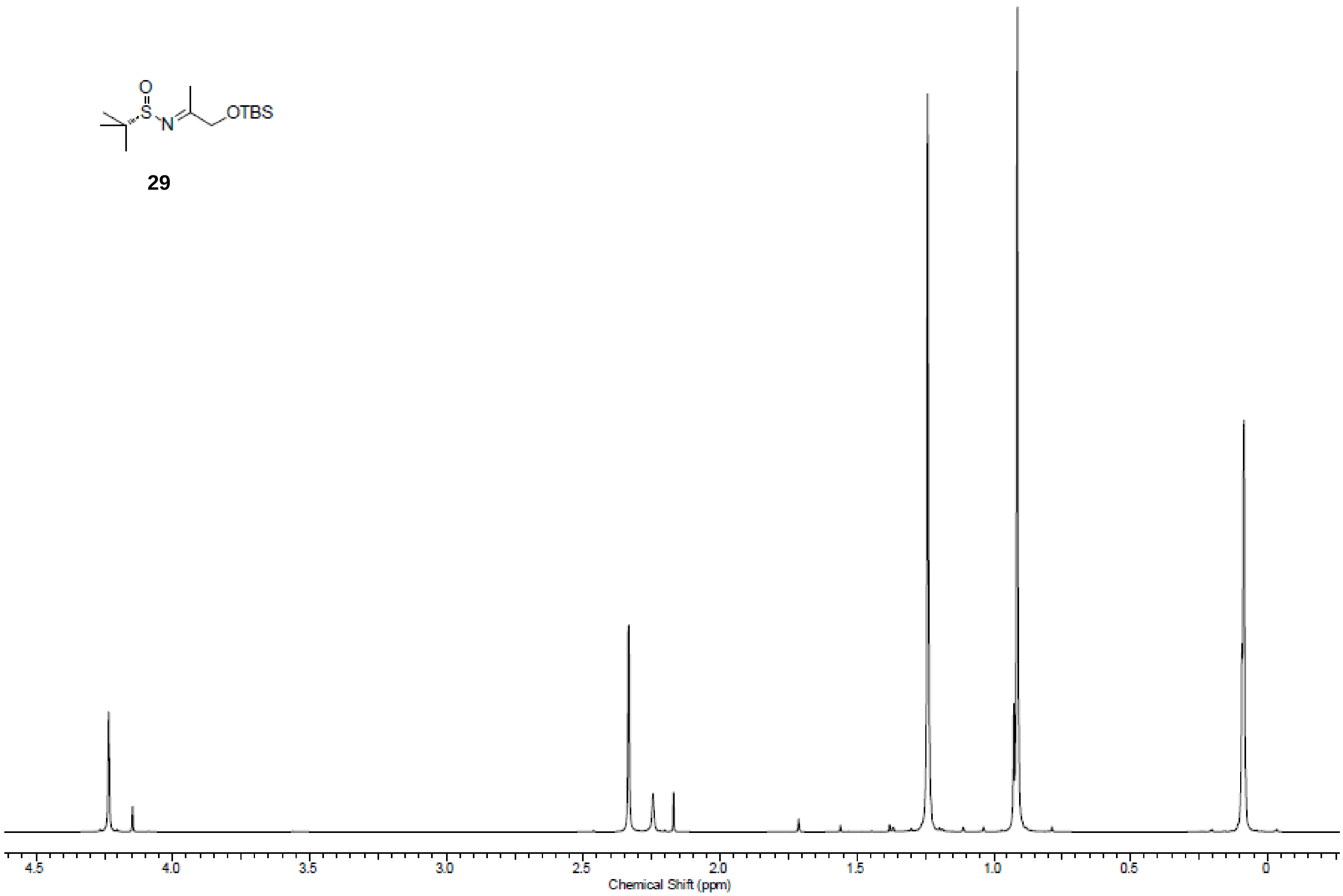
O
S
N
OTBS
29
4.5
4.0
3.5
3.0
2.5
2.0
1.5
1.0
0.5
0
Chemical Shift (ppm)

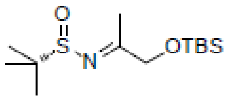
O
S
N
OTBS

29

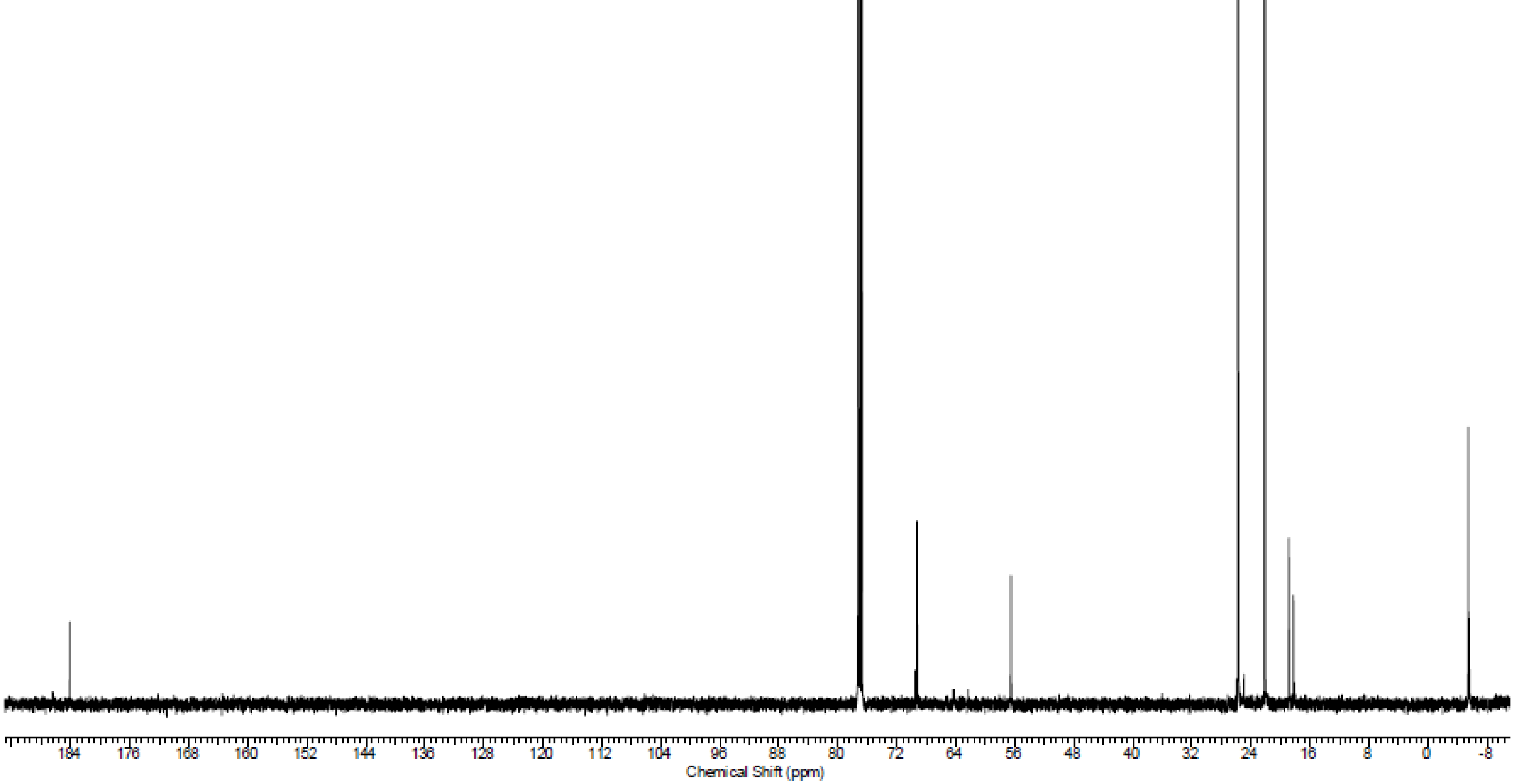
184
176
168
160
152
144
136
128
120
112
104
96
88
80
72
64
56
48
40
32
24
16
8
0
-8
Chemical Shift (ppm)

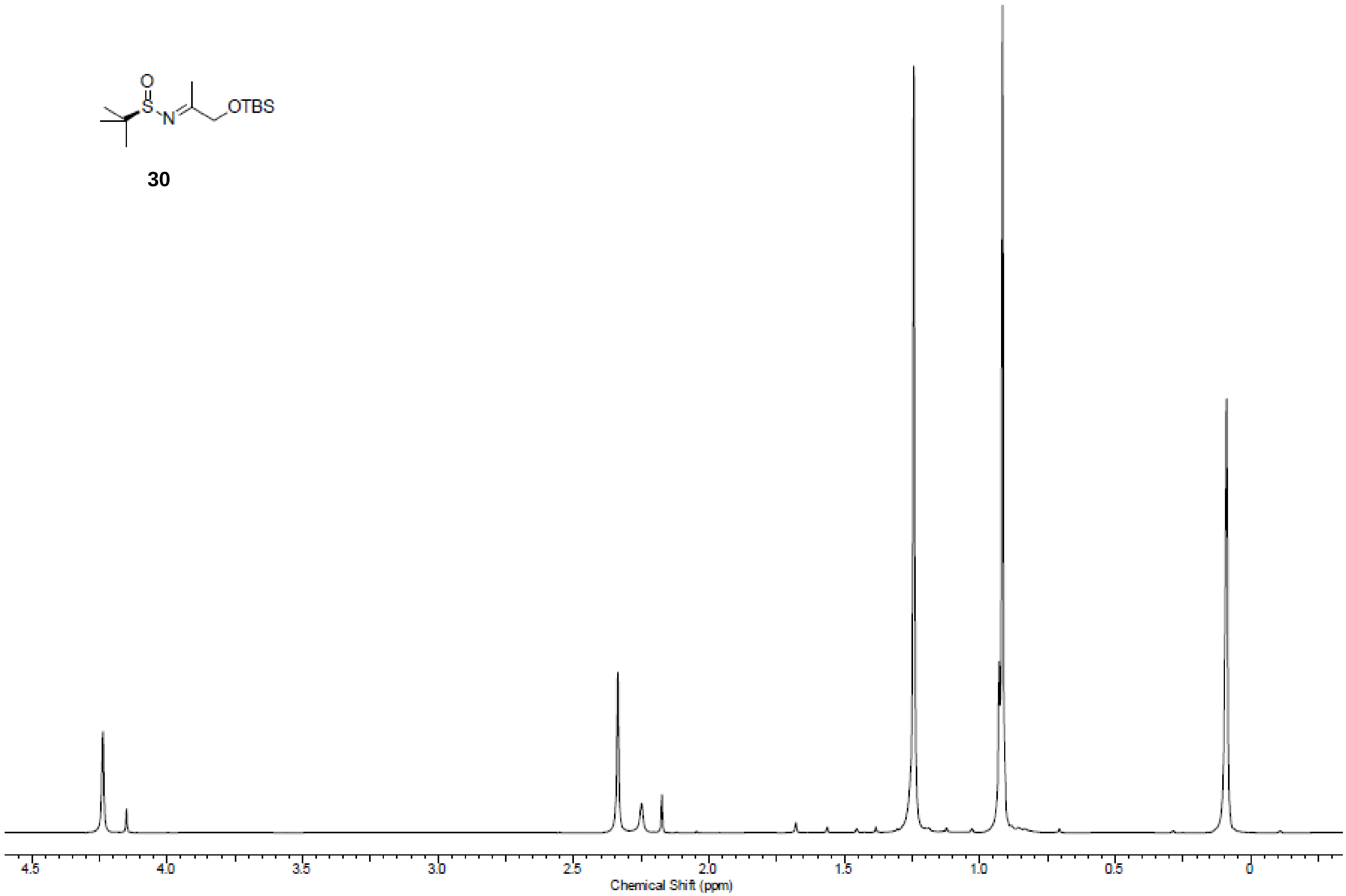

30

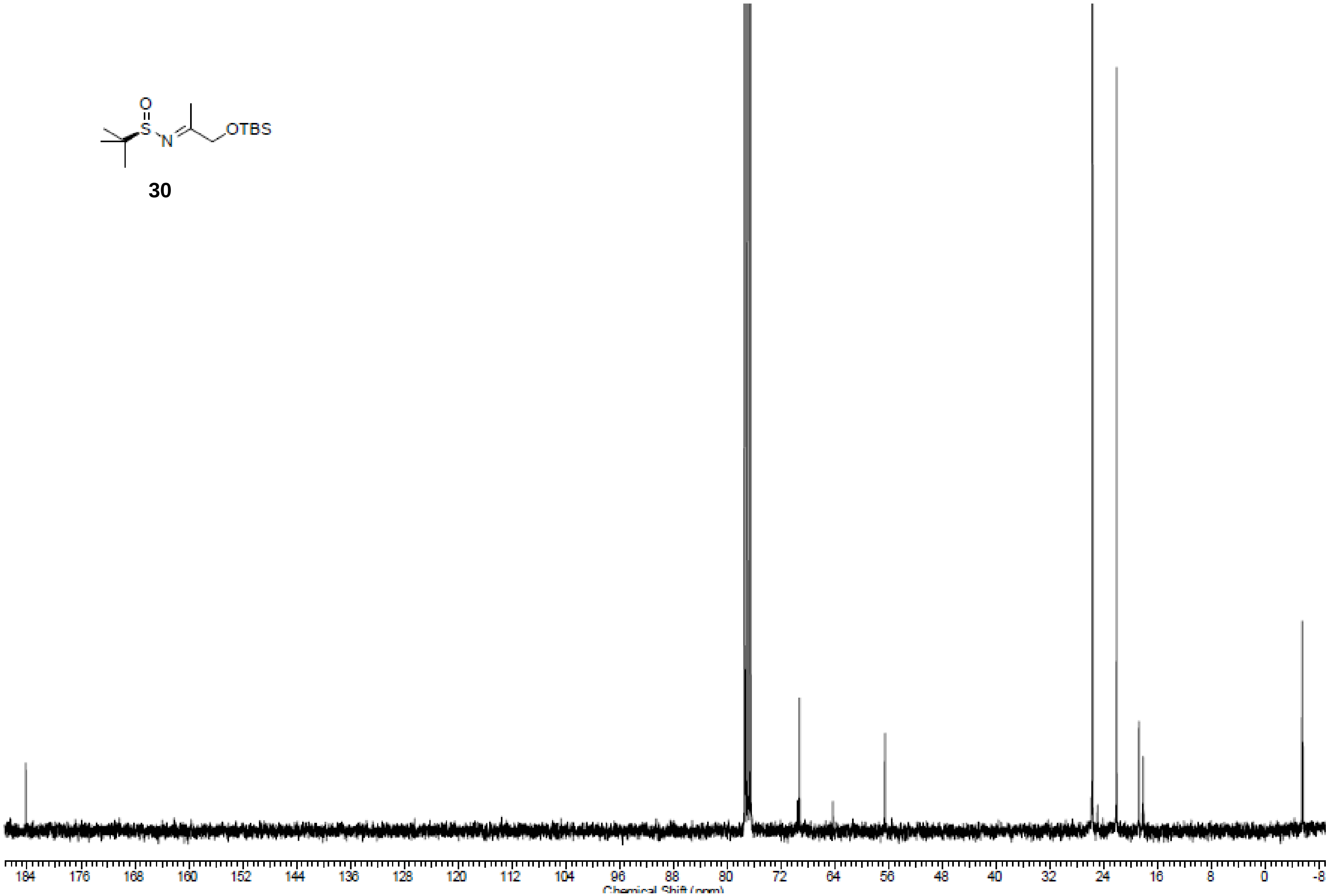
O
S
N
OTBS
184 176 168 160 152 144 136 128 120 112 104 96 88 80 72 64 56 48 40 32 24 16 8 0 -8

30

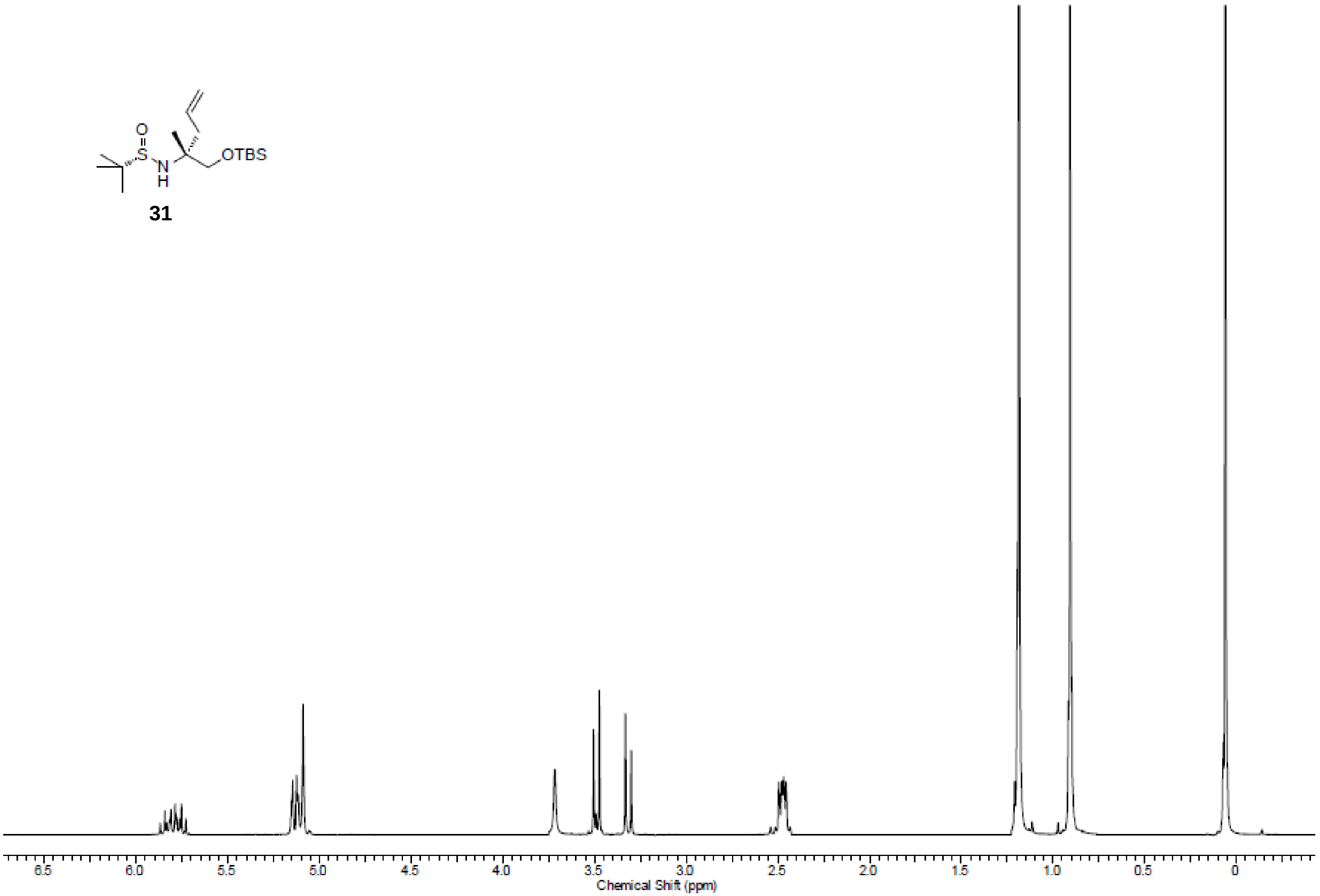

O
S
N
H
OTBS
31
6.5
6.0
5.5
5.0
4.5
4.0
3.5
3.0
2.5
2.0
1.5
1.0
0.5
0
Chemical Shift (ppm)

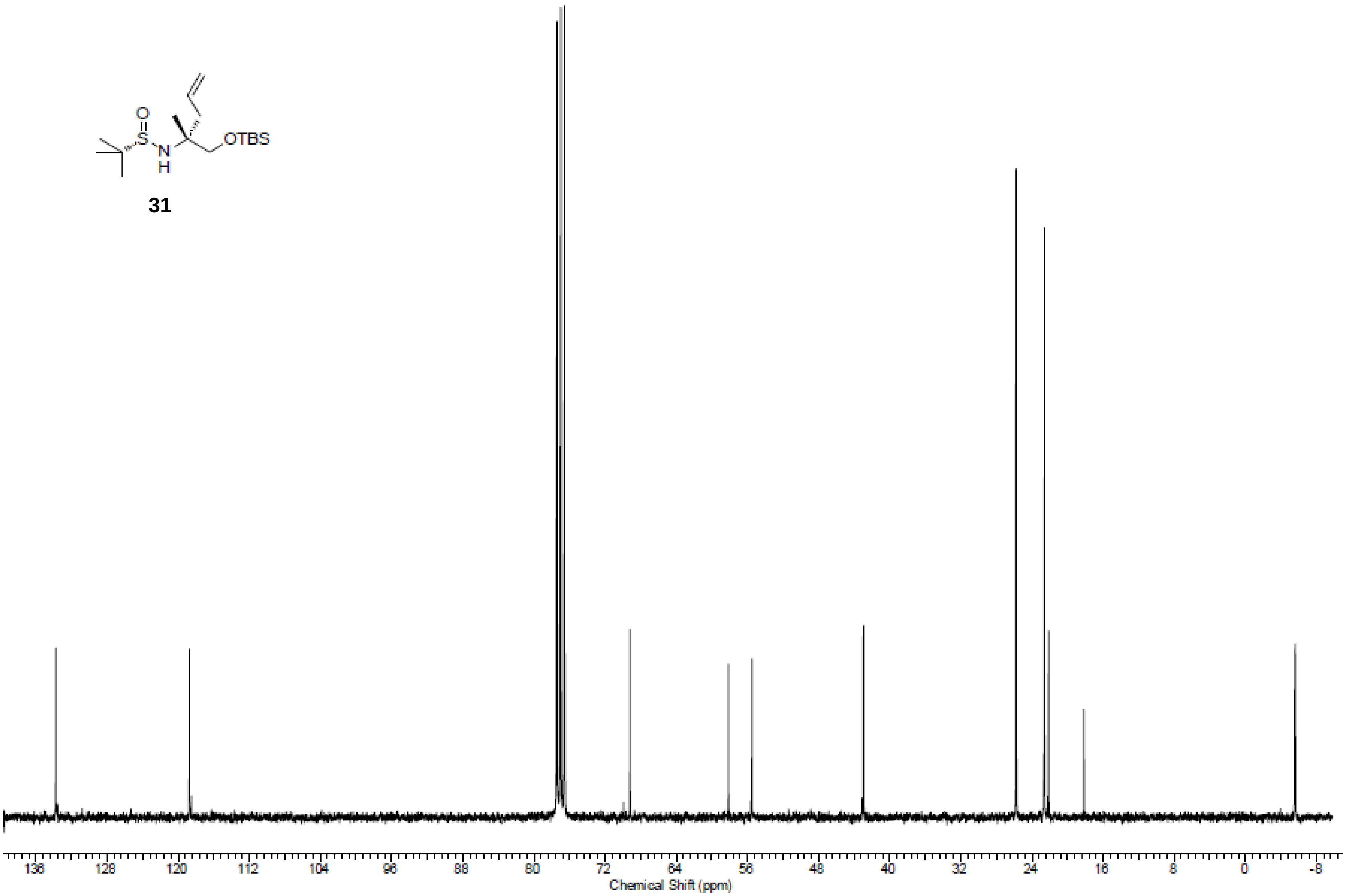
O
S
N
H
OTBS
136
128
120
112
104
96
88
80
72
64
56
48
40
32
24
16
8
0
-8
Chemical Shift (ppm)

31

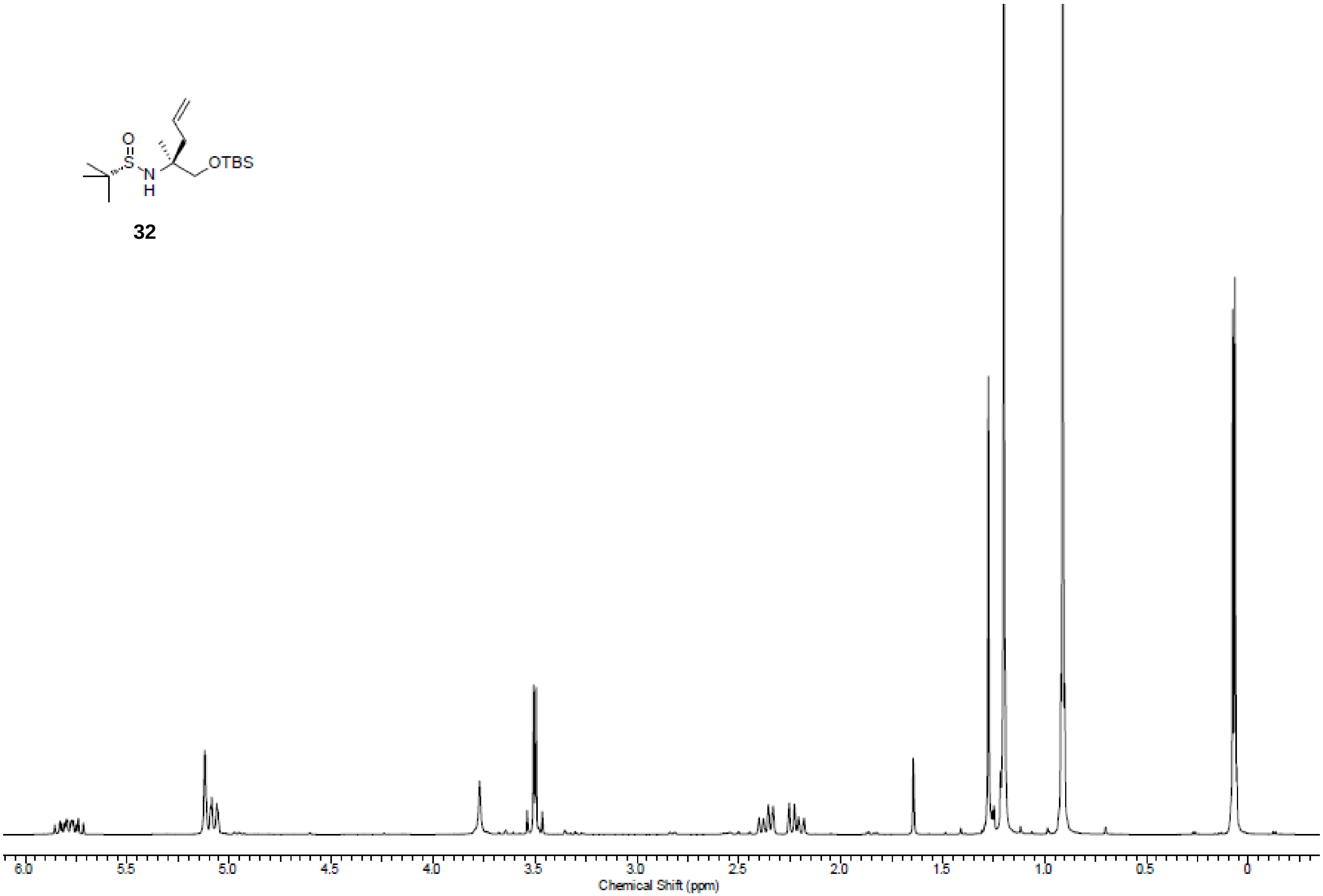
O
S
N
H
OTBS
32
6.0
5.5
5.0
4.5
4.0
3.5
3.0
2.5
2.0
1.5
1.0
0.5
0
Chemical Shift (ppm)

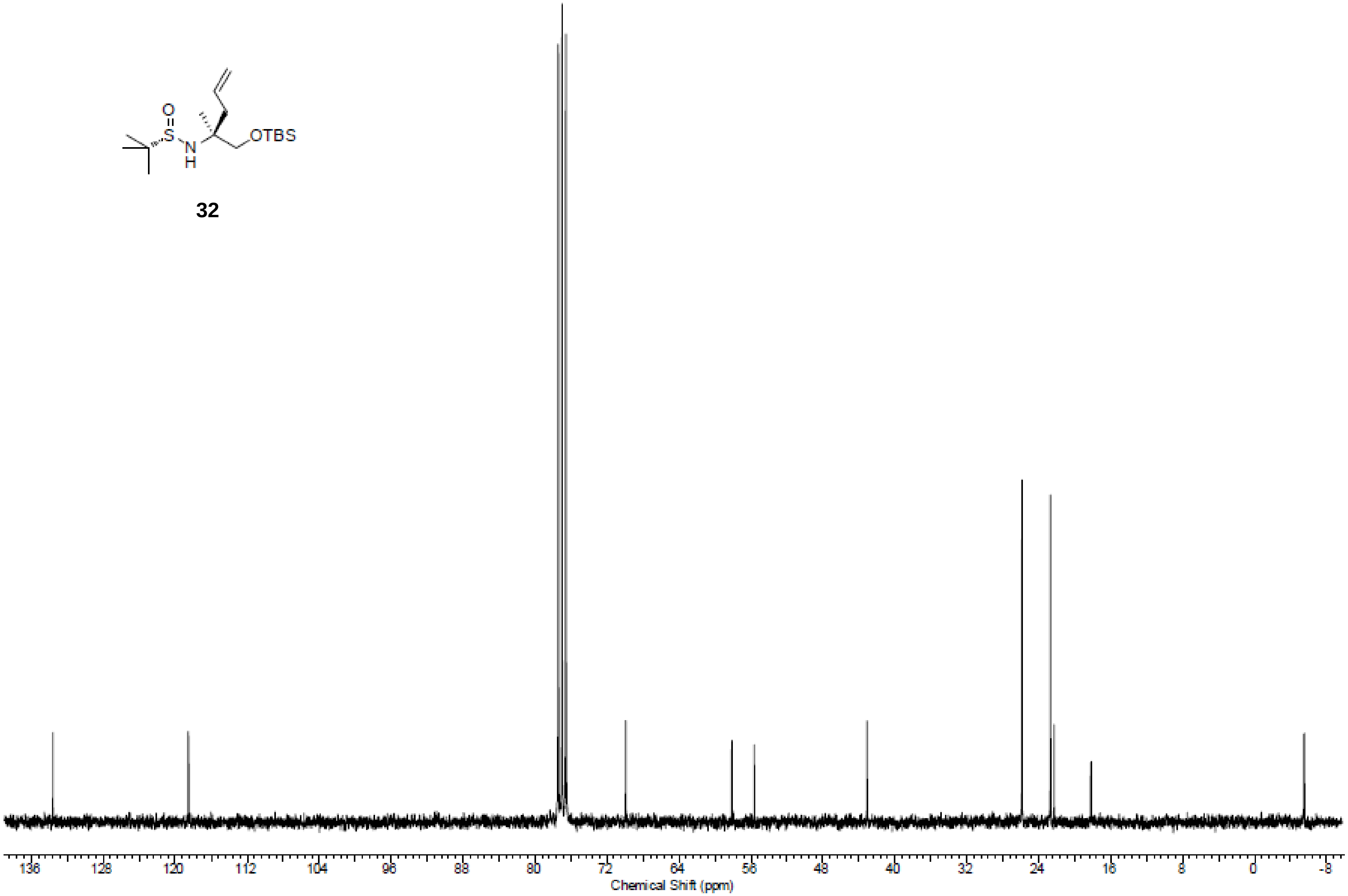
O
S
N
H
OTBS
32
136 128 120 112 104 96 88 80 72 64 56 48 40 32 24 16 8 0 -8
Chemical Shift (ppm)

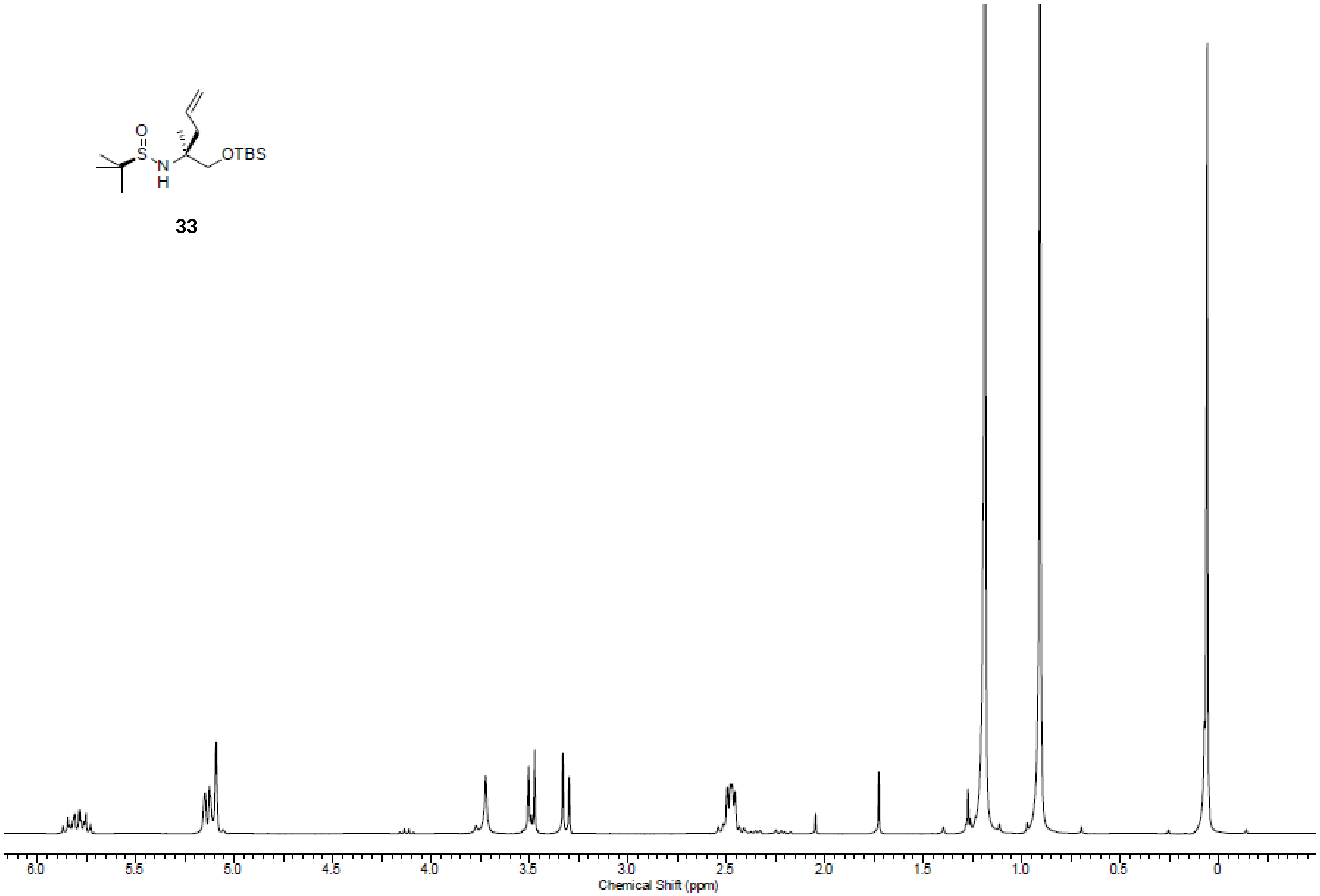
O
S
N
H
OTBS
33
6.0
5.5
5.0
4.5
4.0
3.5
3.0
2.5
2.0
1.5
1.0
0.5
0
Chemical Shift (ppm)

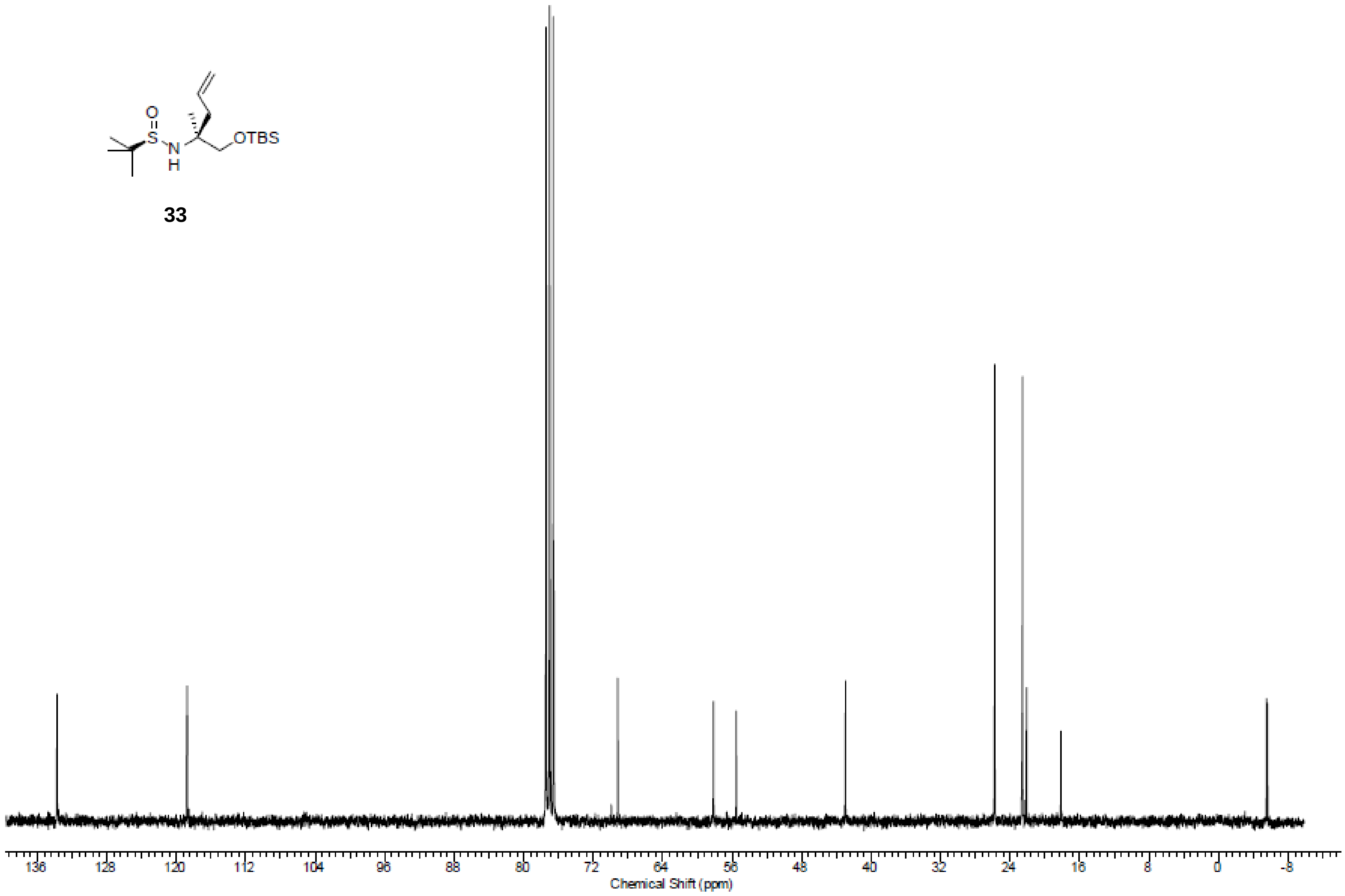
O
S
N
H
OTBS
33
136
128
120
112
104
96
88
80
72
64
56
48
40
32
24
16
8
0
-8
Chemical Shift (ppm)

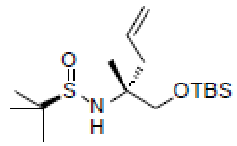

34

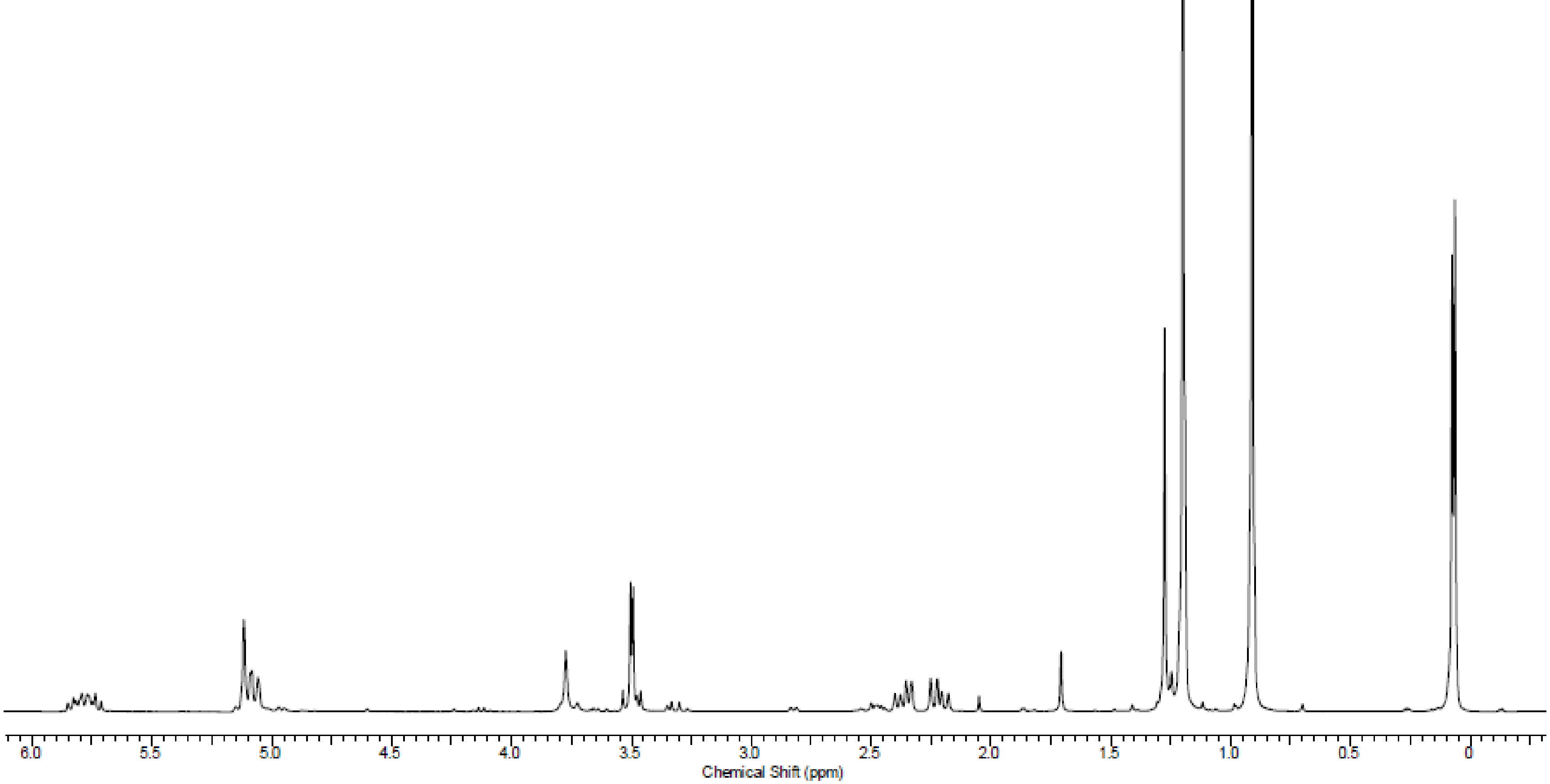

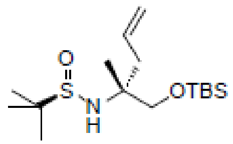
O
S
N
H
OTBS

34

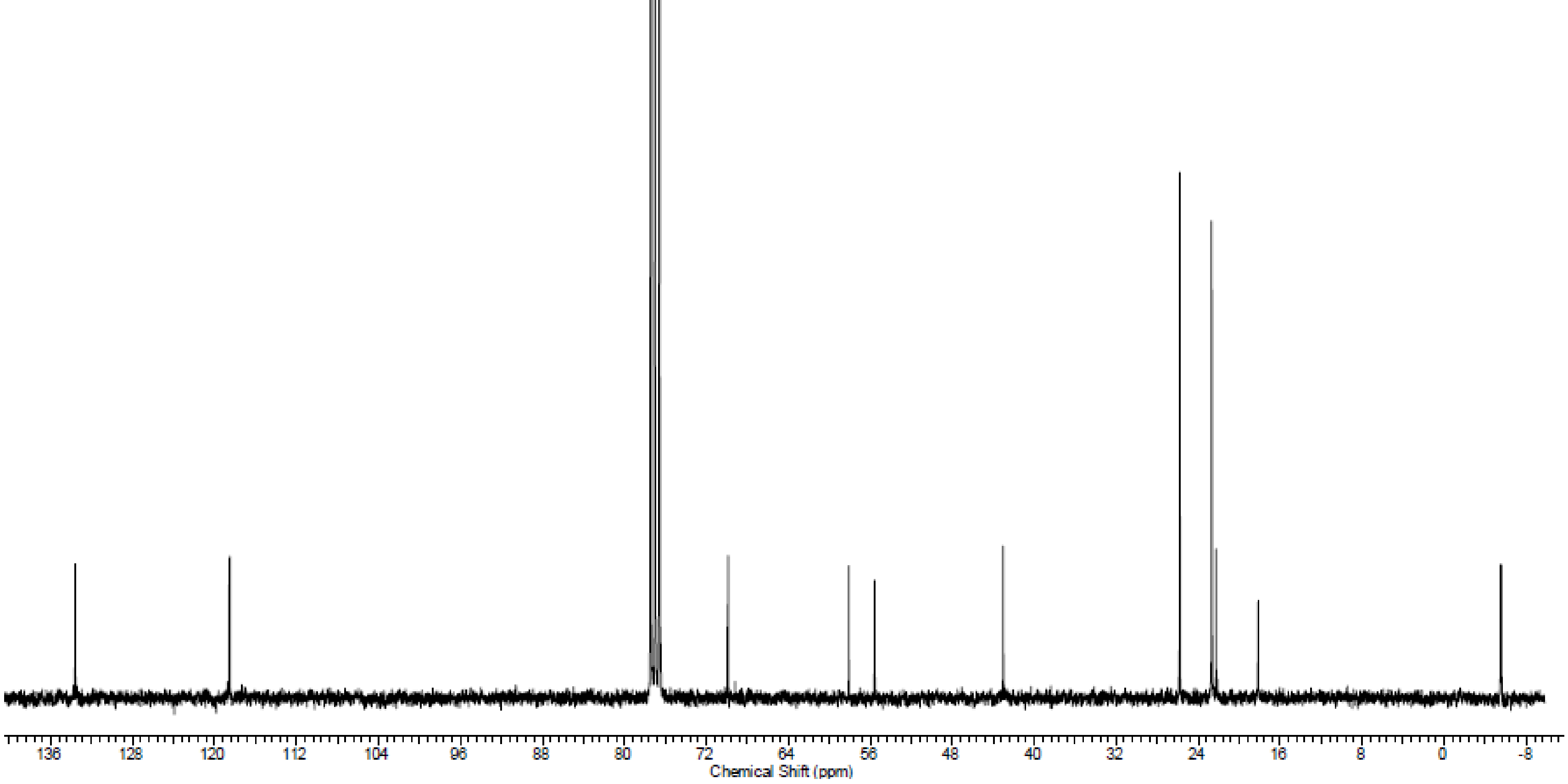
136
128
120
112
104
96
88
80
72
64
56
48
40
32
24
16
8
0
-8
Chemical Shift (ppm)

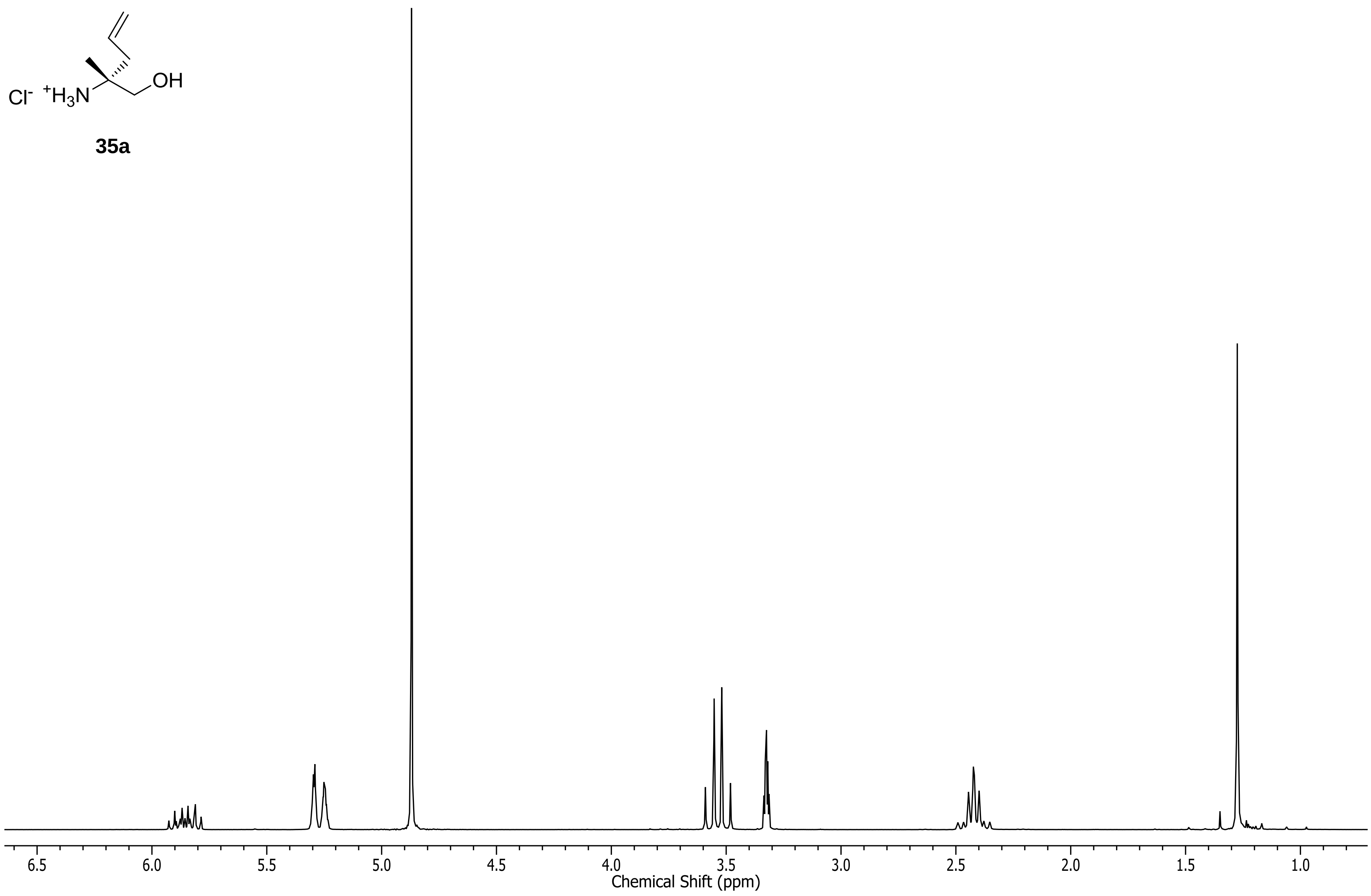
Cl- +H3N
OH
35a
6.5 6.0 5.5 5.0 4.5 4.0 3.5 3.0 2.5 2.0 1.5 1.0
Chemical Shift (ppm)

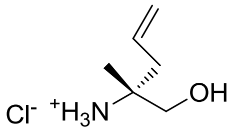
Cl- +H3N
OH

35a

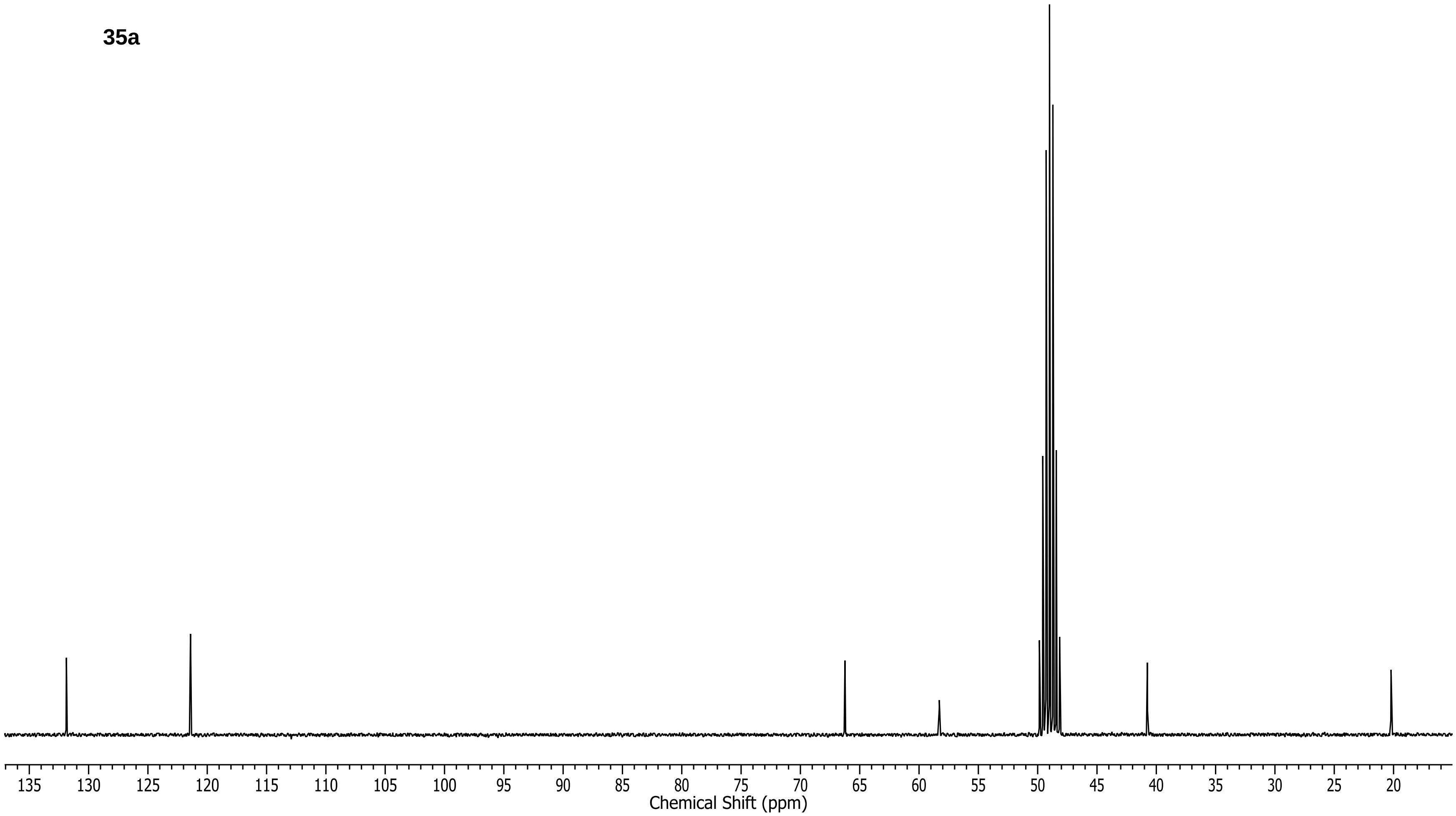
135 130 125 120 115 110 105 100 95 90 85 80 75 70 65 60 55 50 45 40 35 30 25 20
Chemical Shift (ppm)

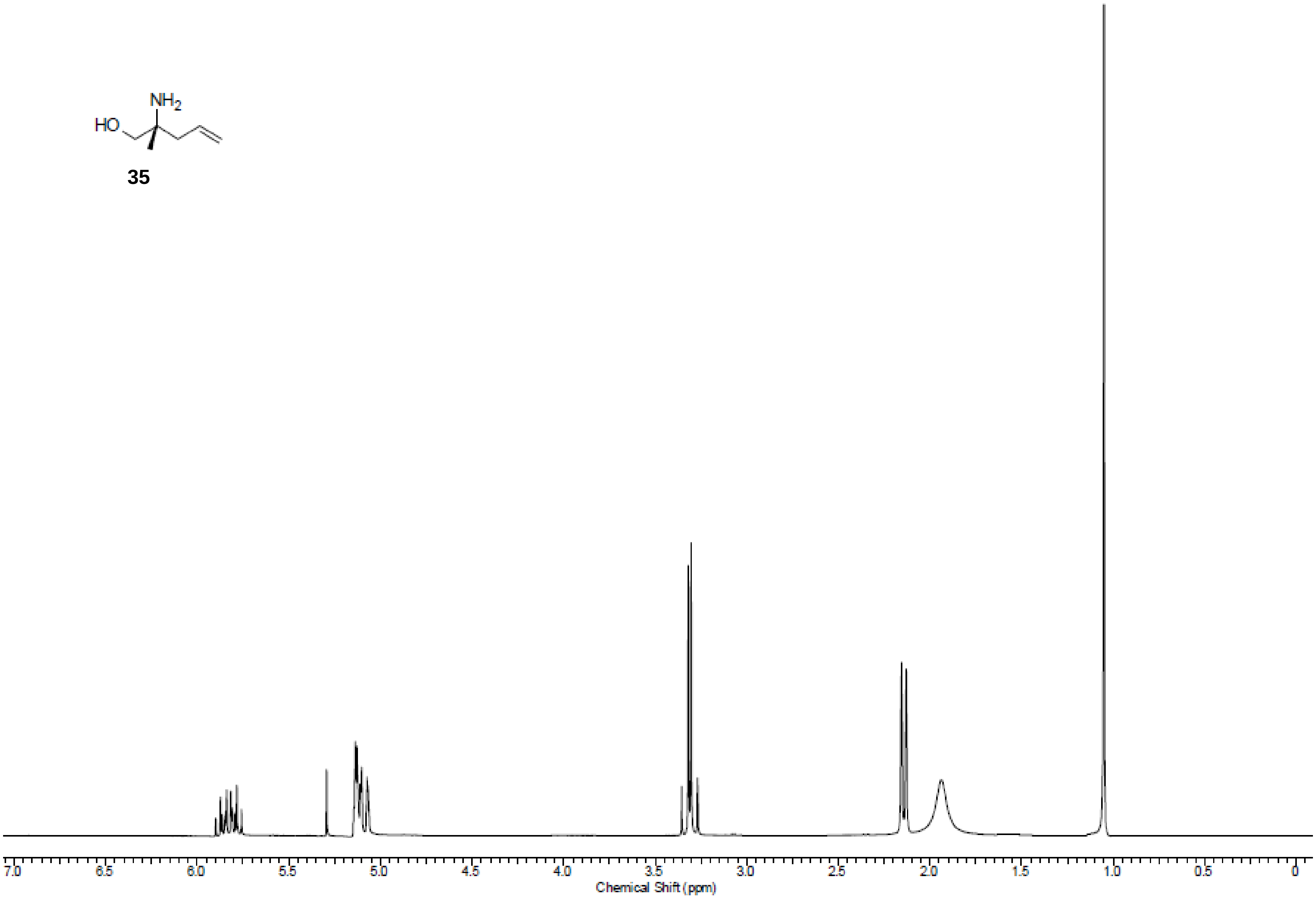
NH2
HO
35
7.0
6.5
6.0
5.5
5.0
4.5
4.0
3.5
3.0
2.5
2.0
1.5
1.0
0.5
0
Chemical Shift (ppm)

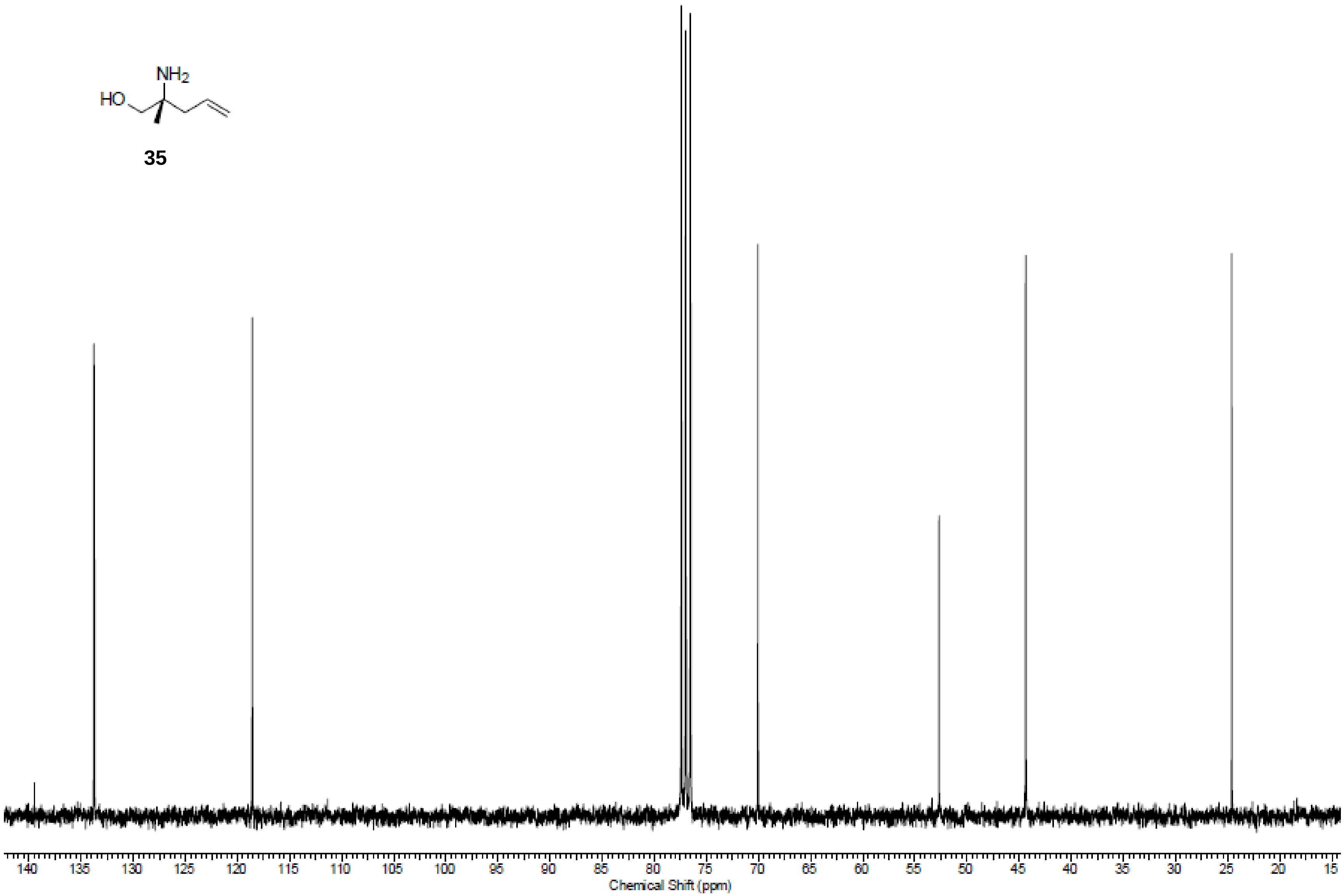
NH2
HO
35
140
135
130
125
120
115
110
105
100
95
90
85
80
75
70
65
60
55
50
45
40
35
30
25
20
15
Chemical Shift (ppm)

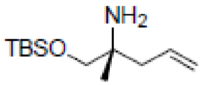
NH2
TBSO

36

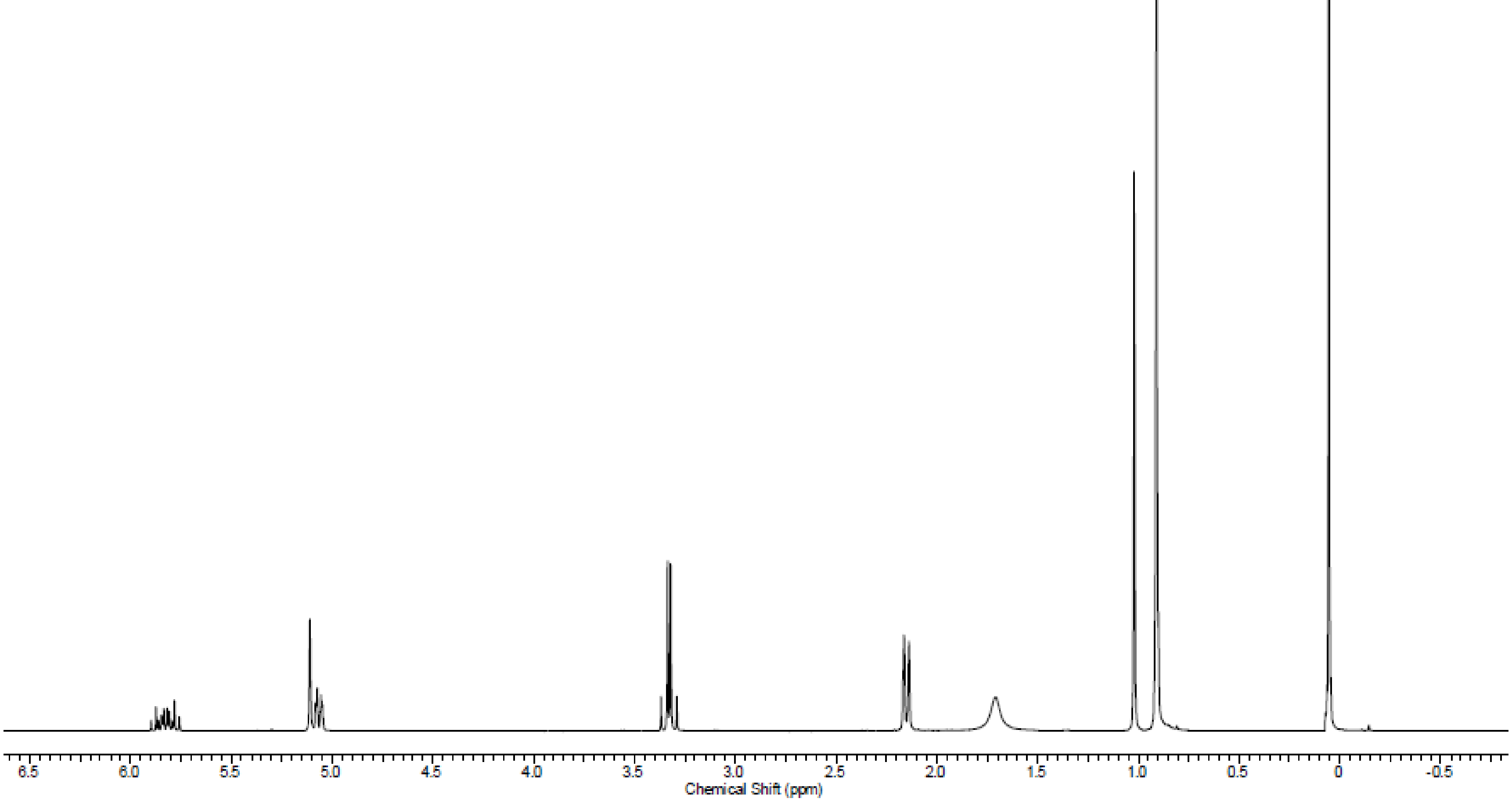
6.5
6.0
5.5
5.0
4.5
4.0
3.5
3.0
2.5
2.0
1.5
1.0
0.5
0
-0.5
Chemical Shift (ppm)

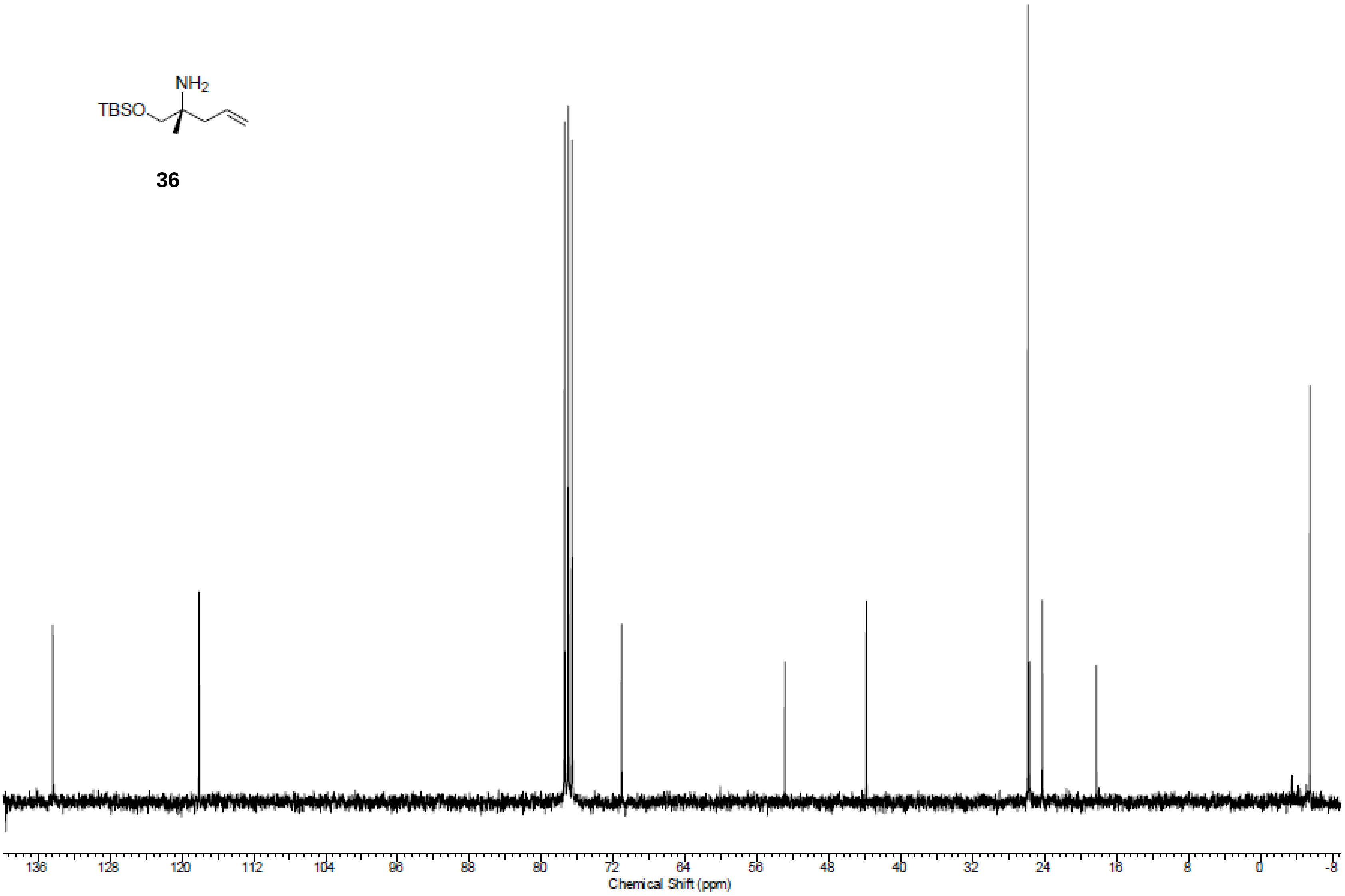
NH2
TBSO
36
136 128 120 112 104 96 88 80 72 64 56 48 40 32 24 16 8 0 -8
Chemical Shift (ppm)

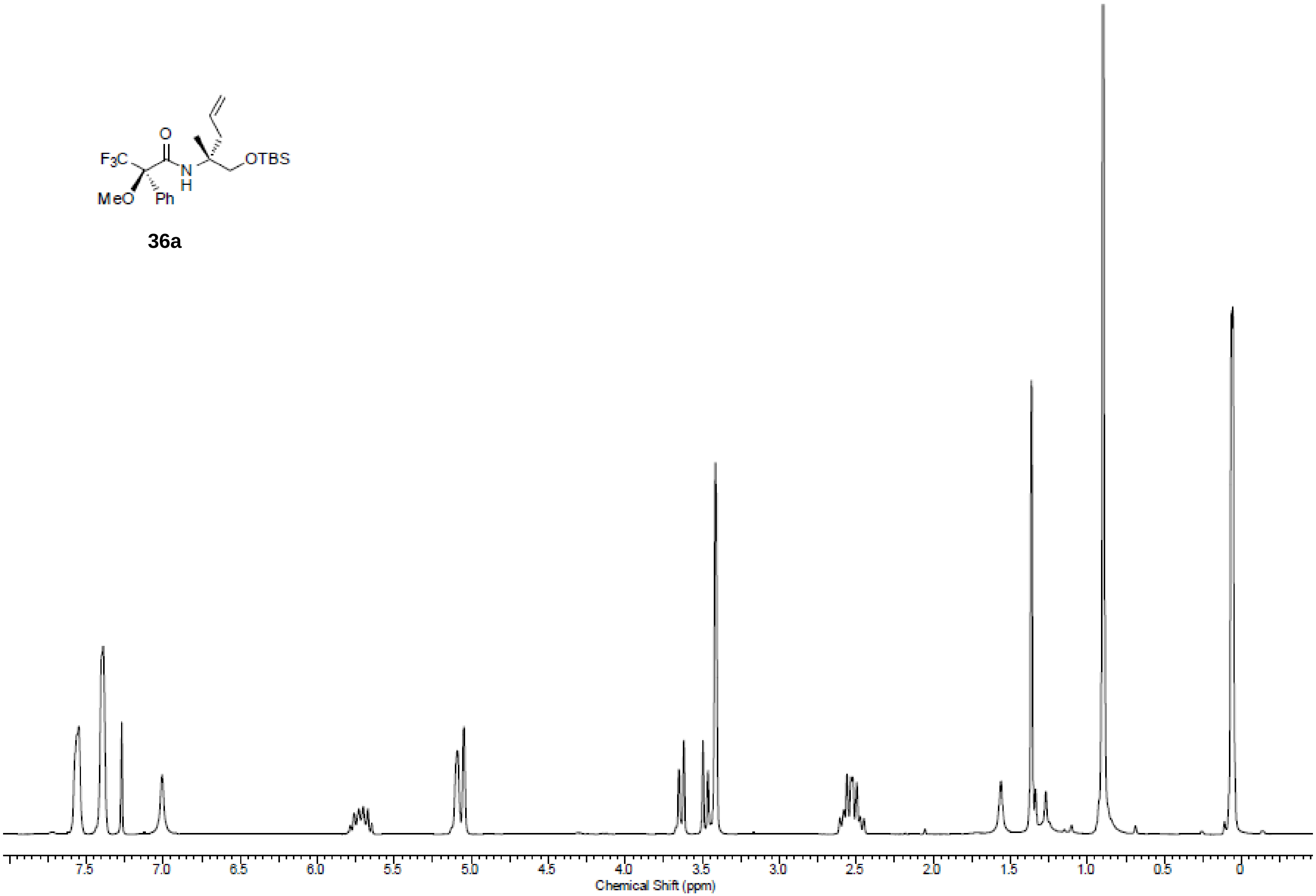

F3C
O
N
H
OTBS
MeO
Ph
7.5
7.0
6.5
6.0
5.5
5.0
4.5
4.0
3.5
3.0
2.5
2.0
1.5
1.0
0.5
0
Chemical Shift (ppm)

36a

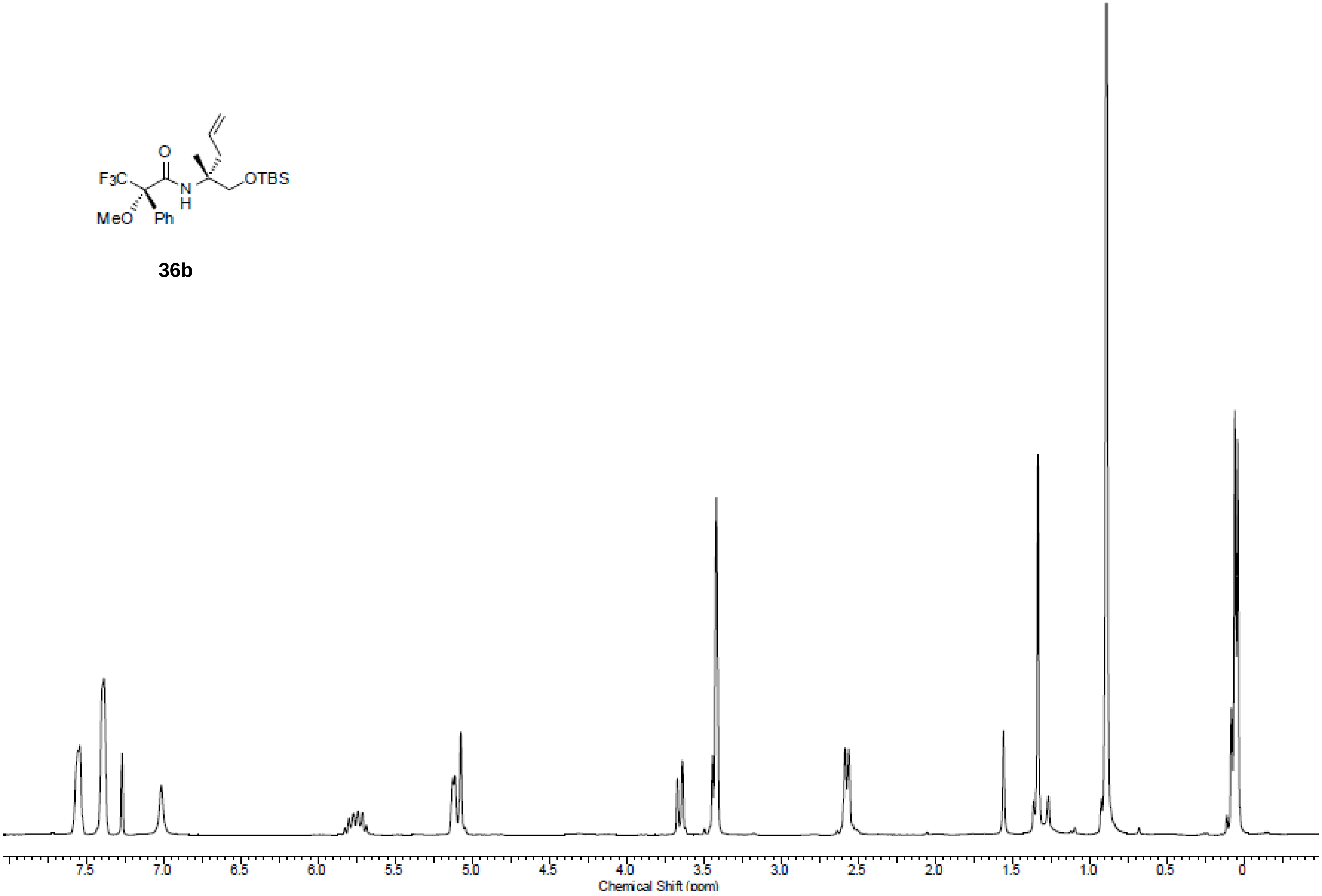
O
F3C
N
H
OTBS
MeO
Ph

36b

7.5
7.0
6.5
6.0
5.5
5.0
4.5
4.0
3.5
3.0
2.5
2.0
1.5
1.0
0.5
0
Chemical Shift (ppm)

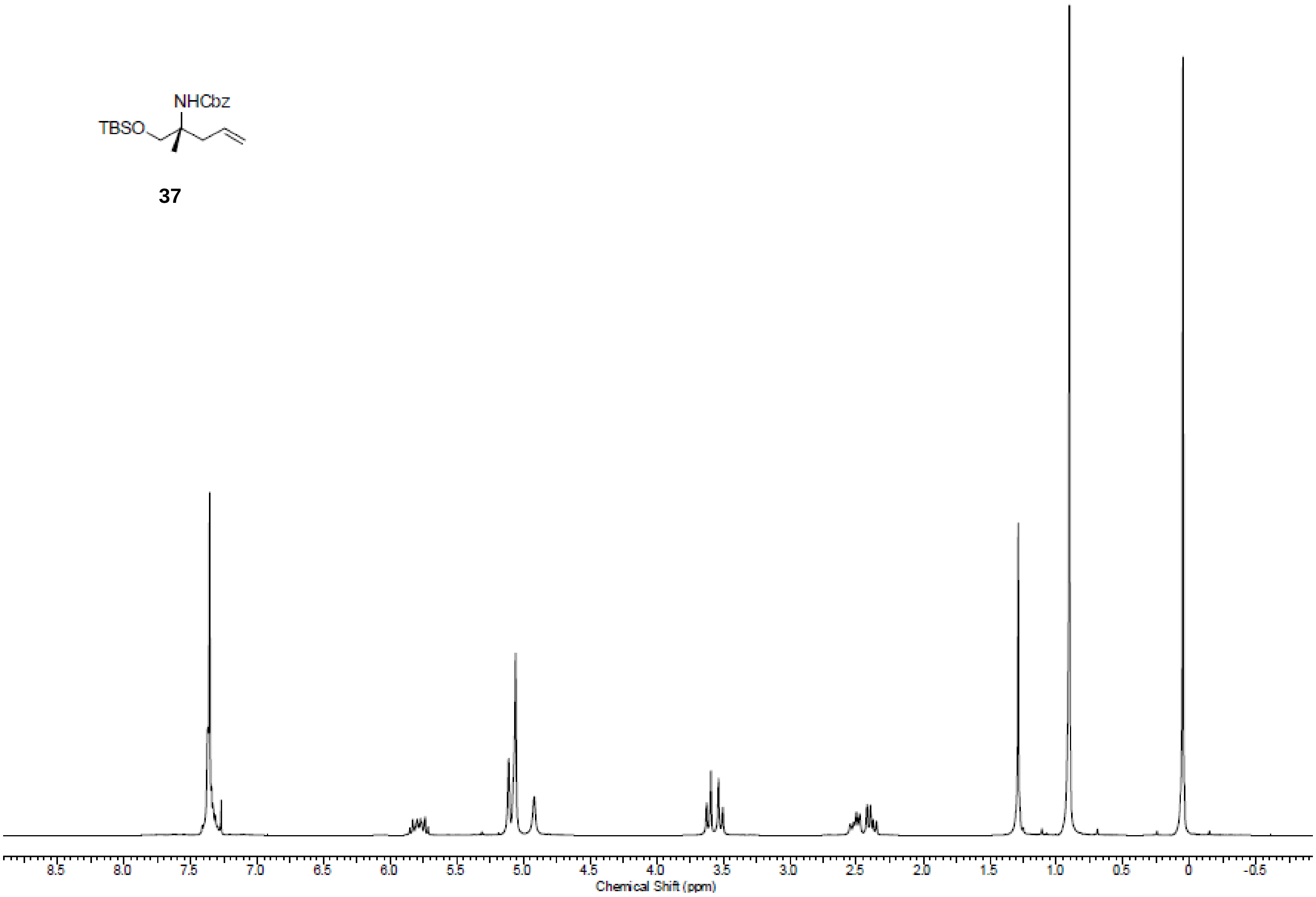

37
8.5
8.0
7.5
7.0
6.5
6.0
5.5
5.0
4.5
4.0
3.5
3.0
2.5
2.0
1.5
1.0
0.5
0
-0.5
Chemical Shift (ppm)

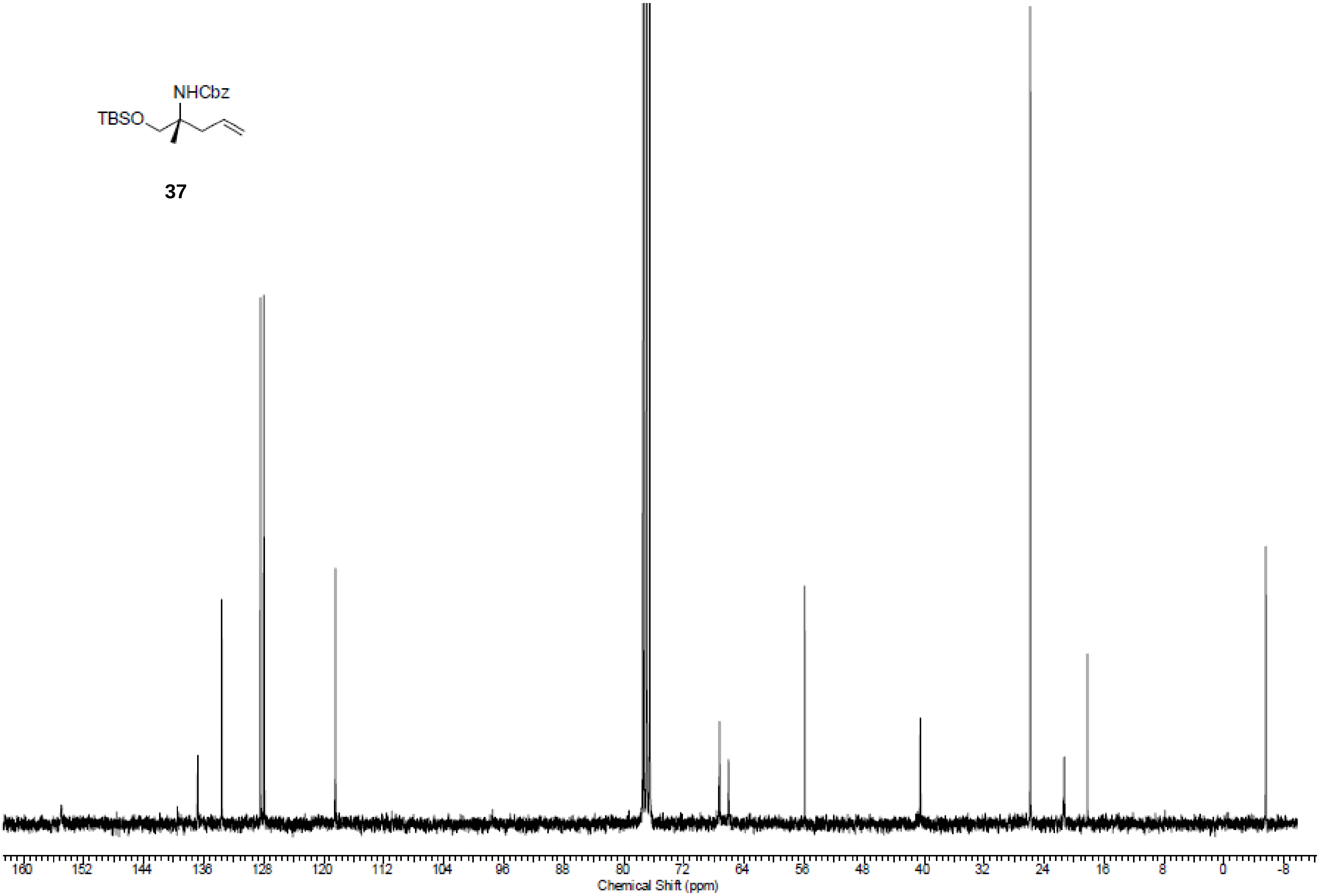
NHCbz
TBSO
37
160 152 144 136 128 120 112 104 96 88 80 72 64 56 48 40 32 24 16 8 0 -8
Chemical Shift (ppm)

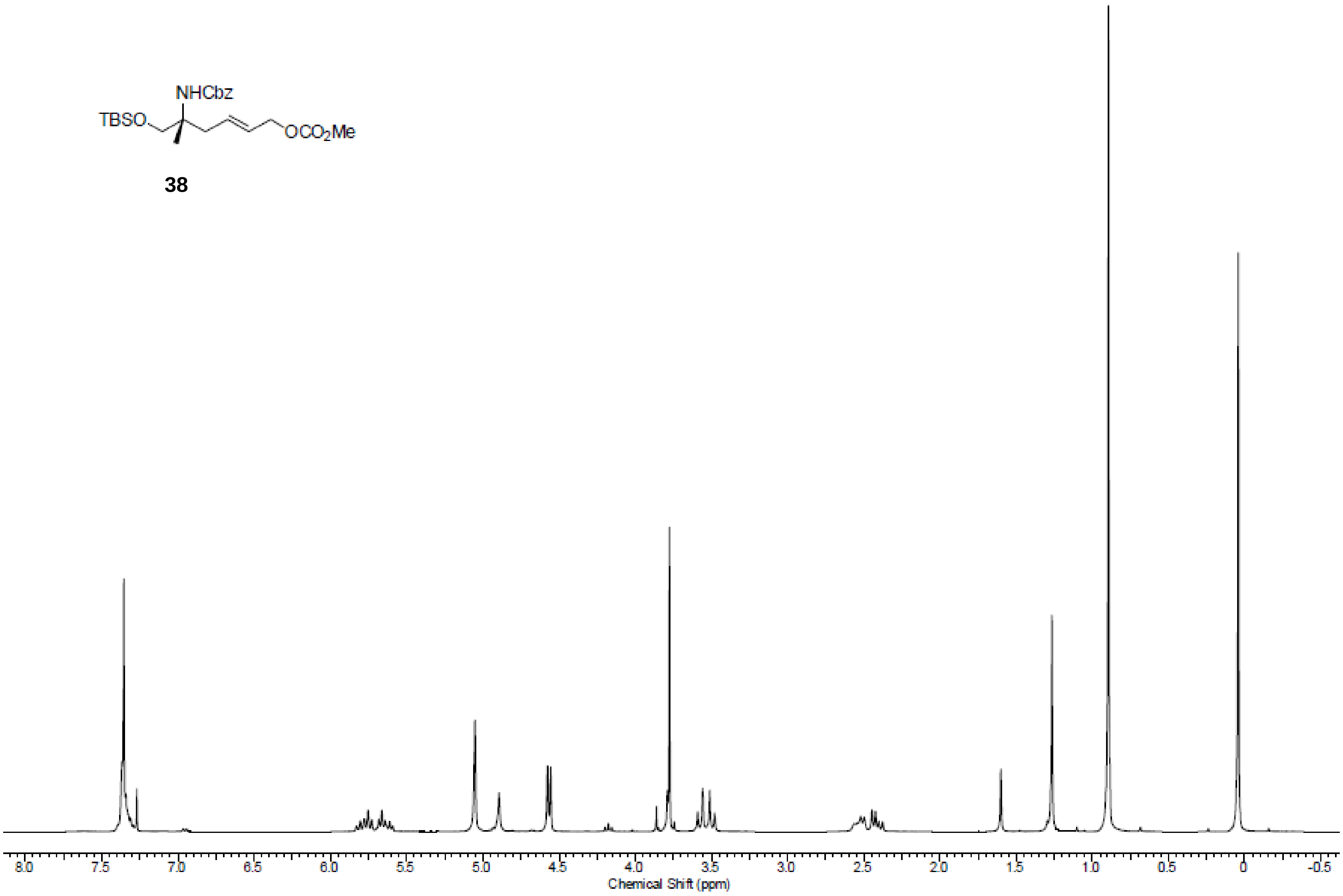
NHCbz
TBSO
OCO2Me
8.0 7.5 7.0 6.5 6.0 5.5 5.0 4.5 4.0 3.5 3.0 2.5 2.0 1.5 1.0 0.5 0 -0.5
Chemical Shift (ppm)

38

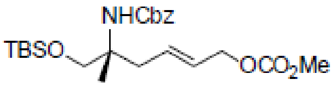
NHCbz
TBSO
OCO2Me

38

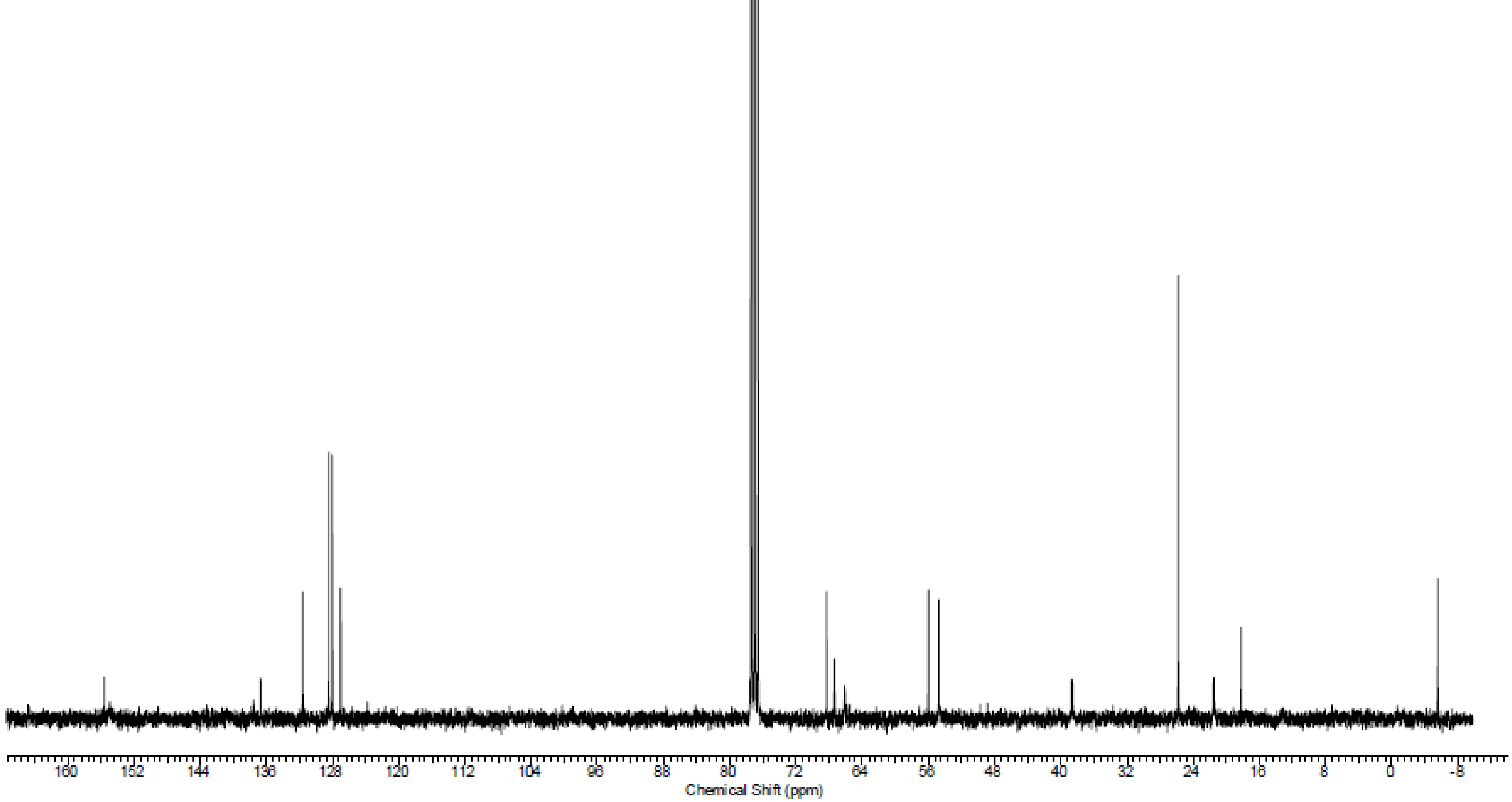
160
152
144
136
128
120
112
104
96
88
80
72
64
56
48
40
32
24
16
8
0
-8
Chemical Shift (ppm)

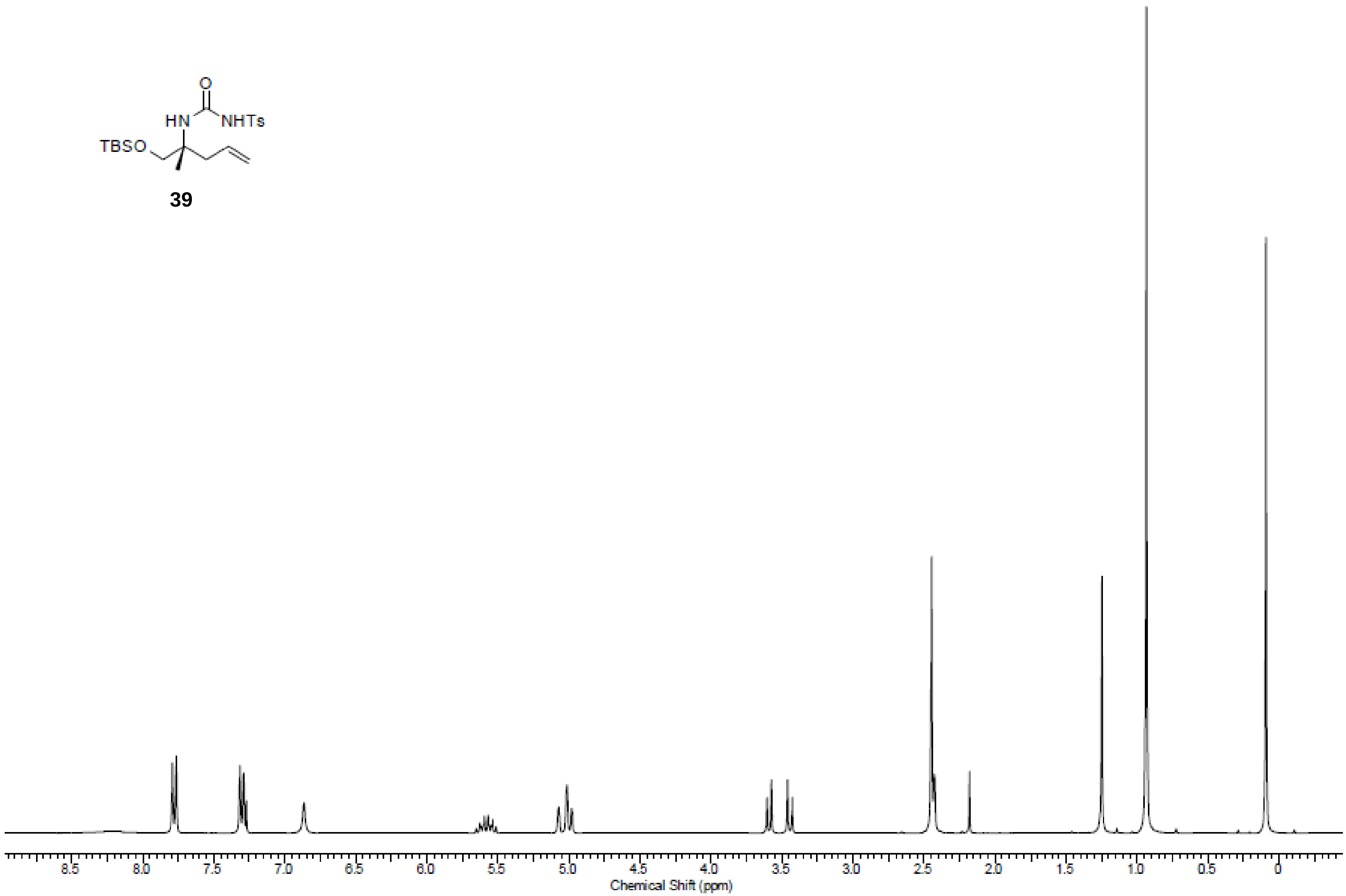
O
HN
NHTs
TBSO
8.5
8.0
7.5
7.0
6.5
6.0
5.5
5.0
4.5
4.0
3.5
3.0
2.5
2.0
1.5
1.0
0.5
0
Chemical Shift (ppm)

39

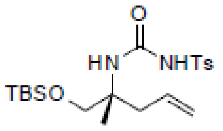

O
HN
NHTs
TBSO

39

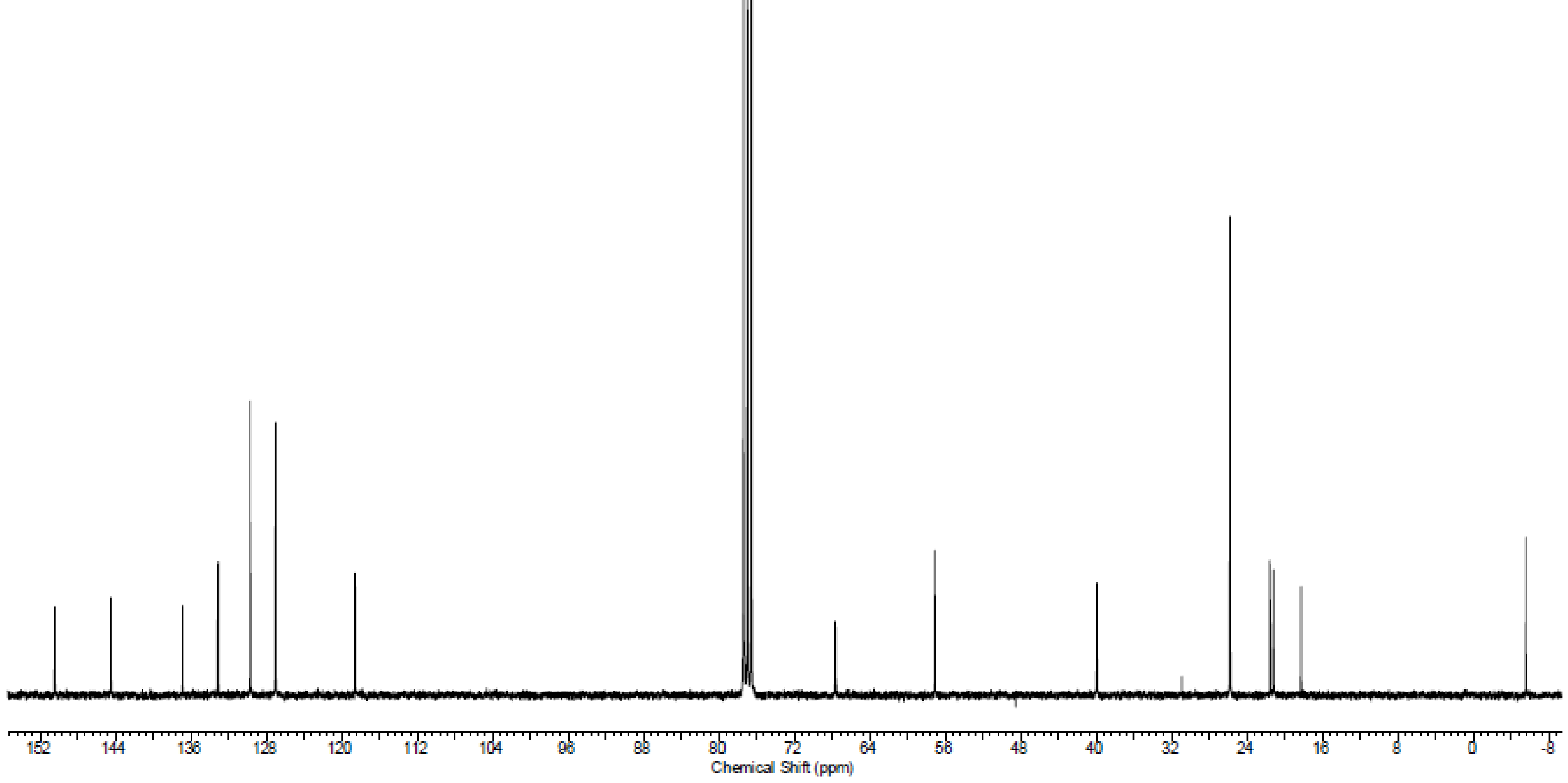

152
144
136
128
120
112
104
96
88
80
72
64
56
48
40
32
24
16
8
0
-8
Chemical Shift (ppm)

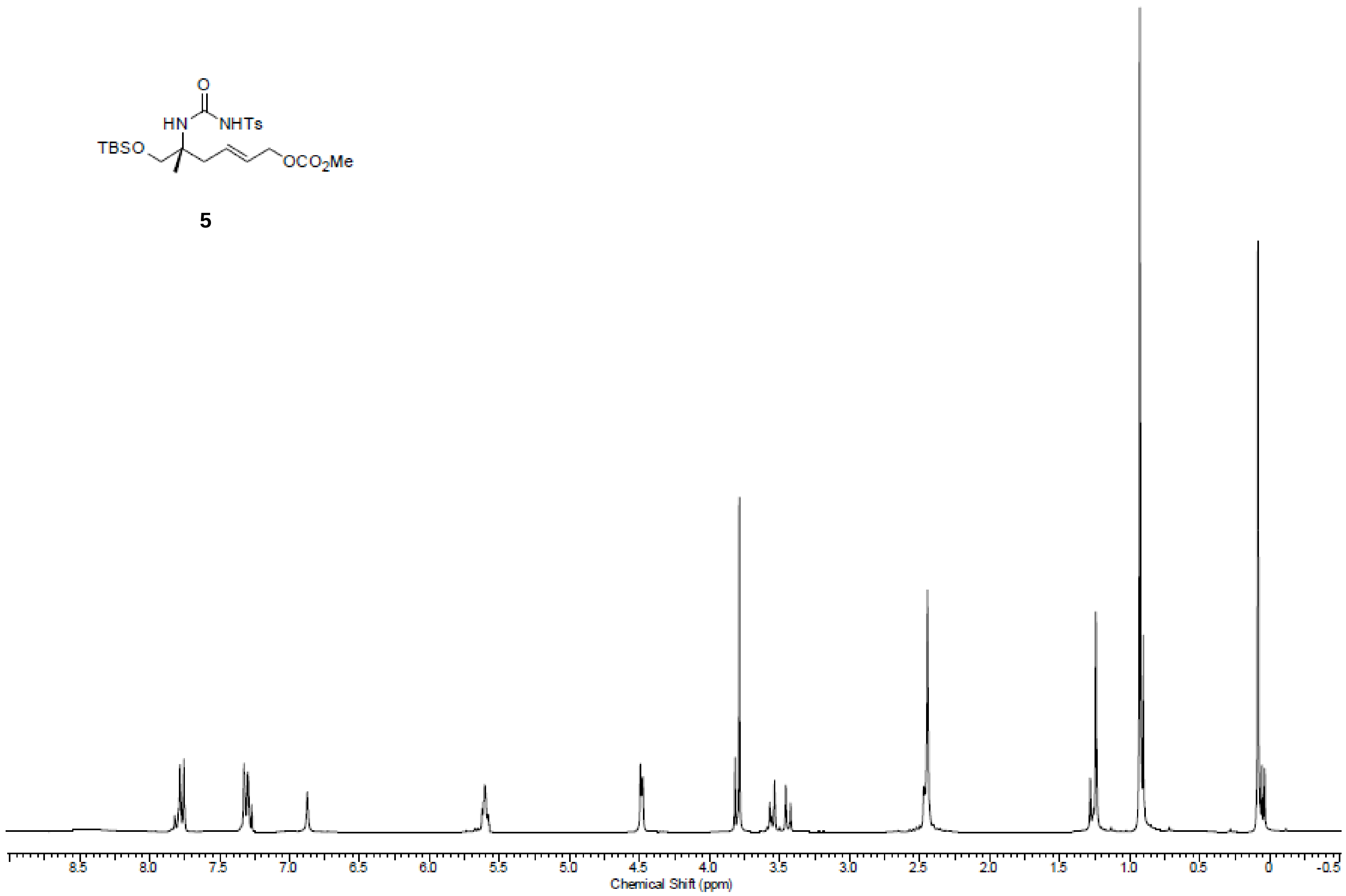

O
HN
NHTs
TBSO
OCO2Me
8.5
8.0
7.5
7.0
6.5
6.0
5.5
5.0
4.5
4.0
3.5
3.0
2.5
2.0
1.5
1.0
0.5
0
-0.5
Chemical Shift (ppm)

5

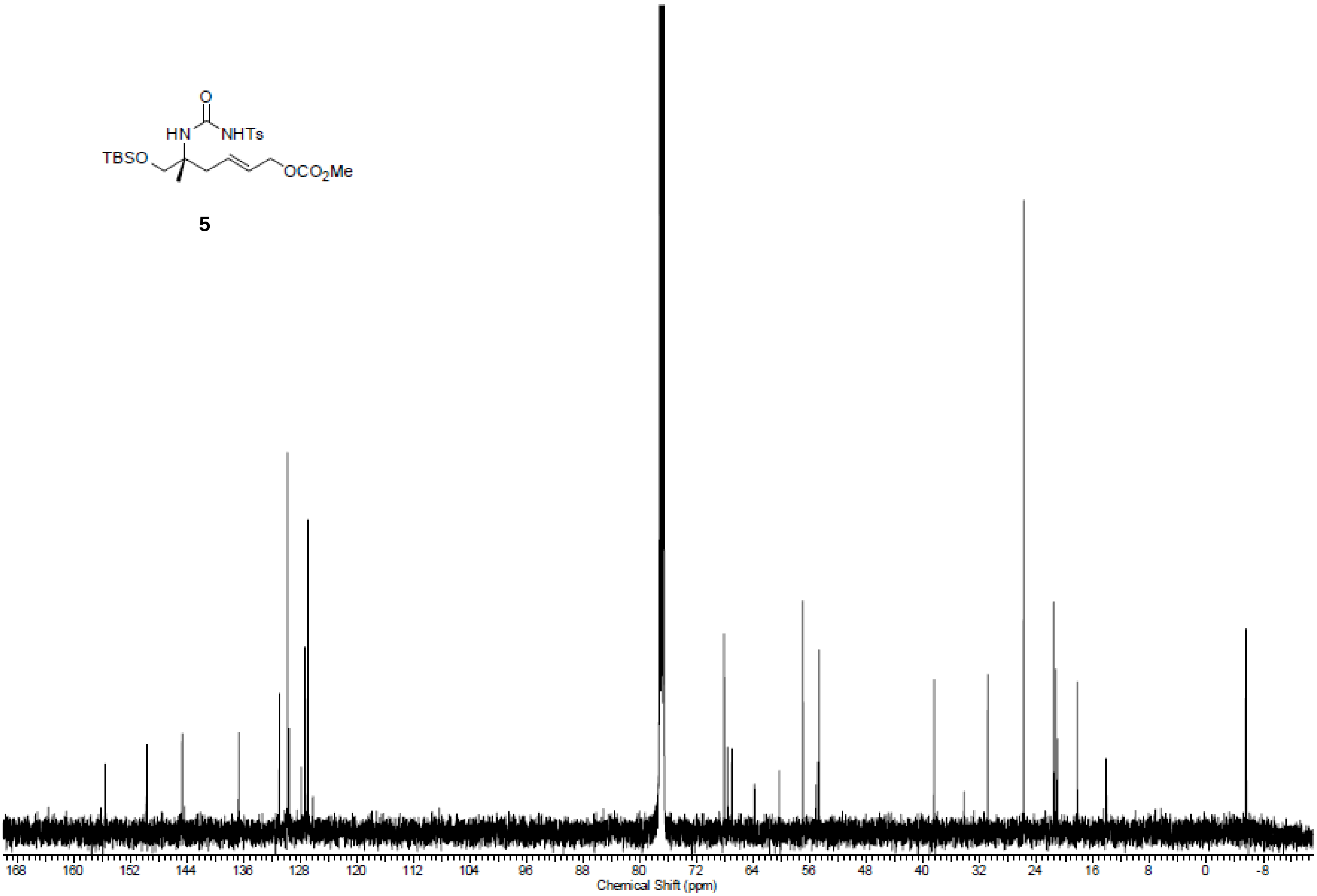
O
HN
NHTs
TBSO
OCO2Me
5
168
160
152
144
136
128
120
112
104
96
88
80
72
64
56
48
40
32
24
16
8
0
-8
Chemical Shift (ppm)

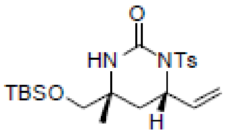
O
HN
NTs
TBSO
H

42

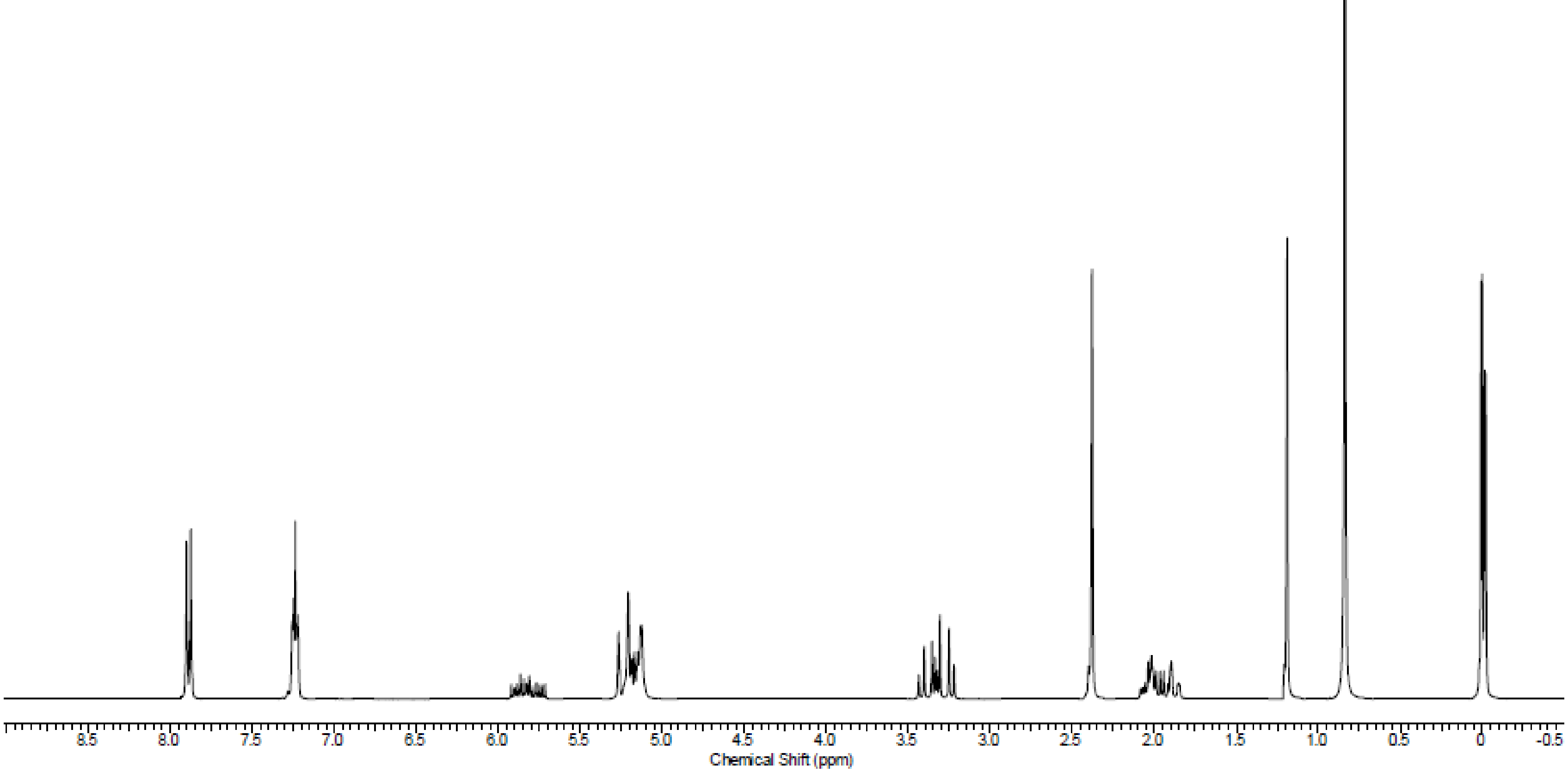
8.5
8.0
7.5
7.0
6.5
6.0
5.5
5.0
4.5
4.0
3.5
3.0
2.5
2.0
1.5
1.0
0.5
0
-0.5
Chemical Shift (ppm)

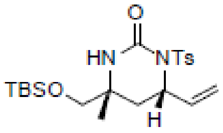
O
HN
NTs
TBSO
H

42

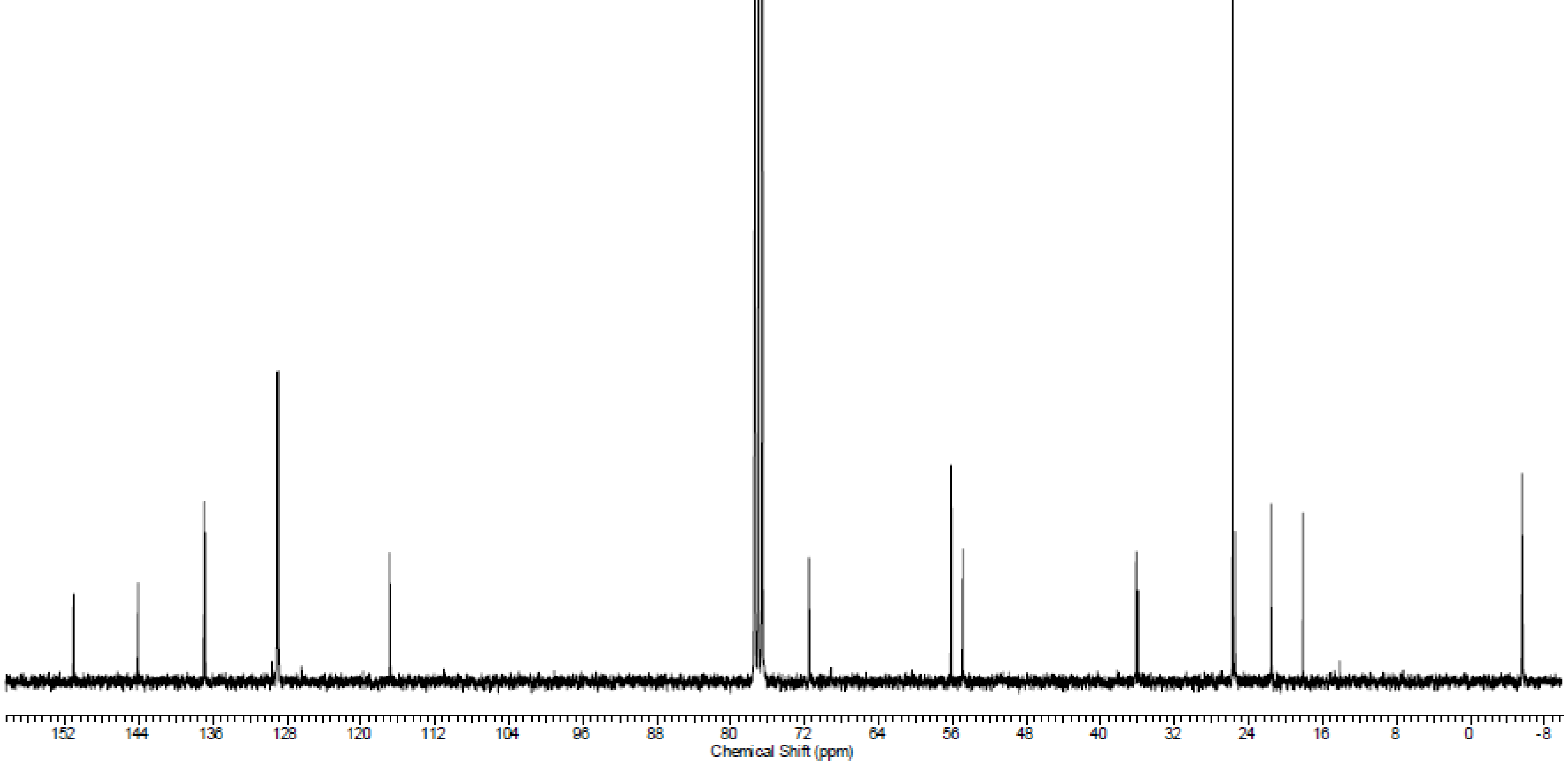
152
144
136
128
120
112
104
96
88
80
72
64
56
48
40
32
24
16
8
0
-8
Chemical Shift (ppm)

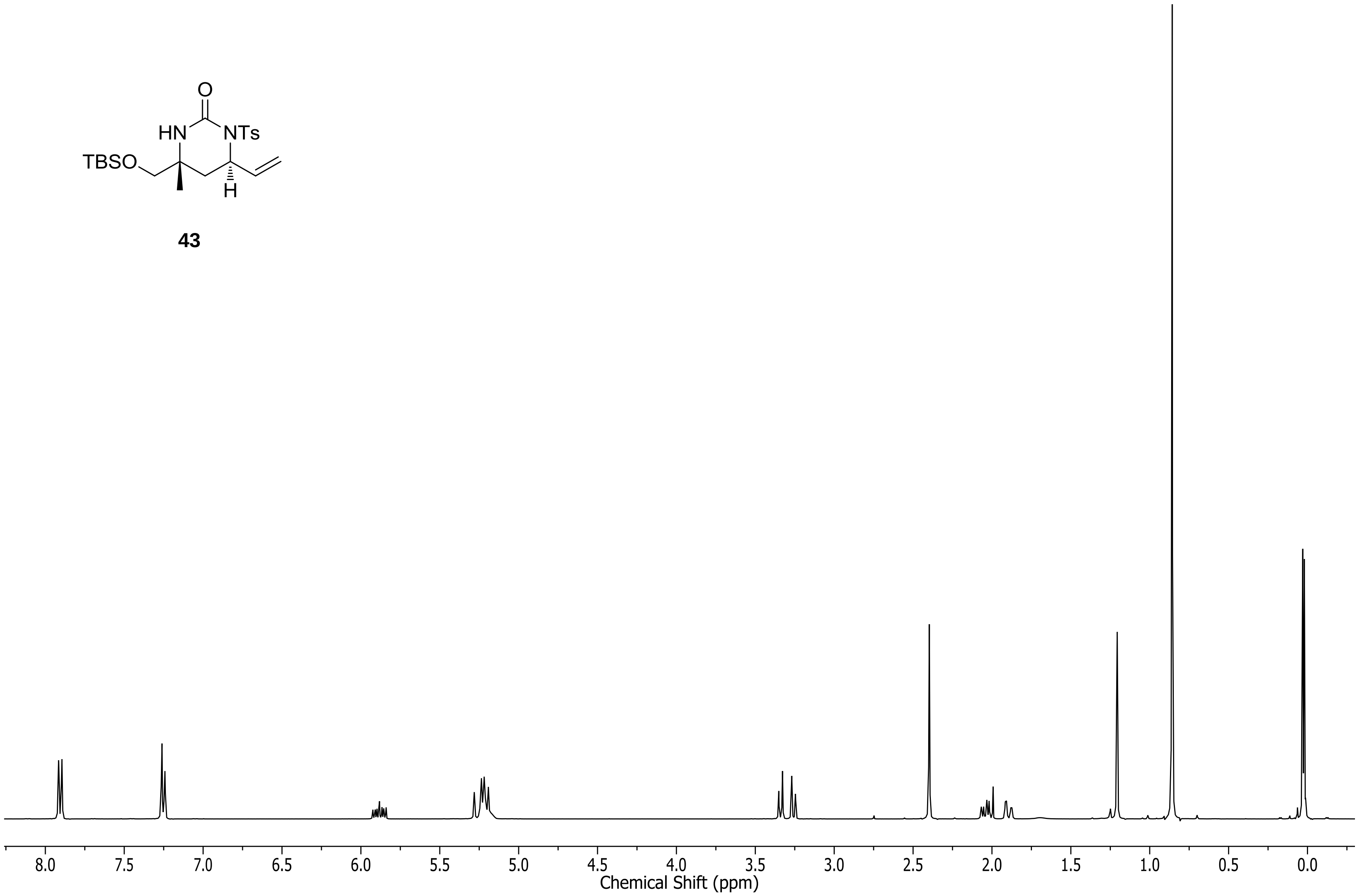
O
HN
NTs
TBSO
H
43
8.0
7.5
7.0
6.5
6.0
5.5
5.0
4.5
4.0
3.5
3.0
2.5
2.0
1.5
1.0
0.5
0.0
Chemical Shift (ppm)

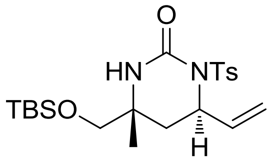
O
HN
NTs
TBSO
H

43

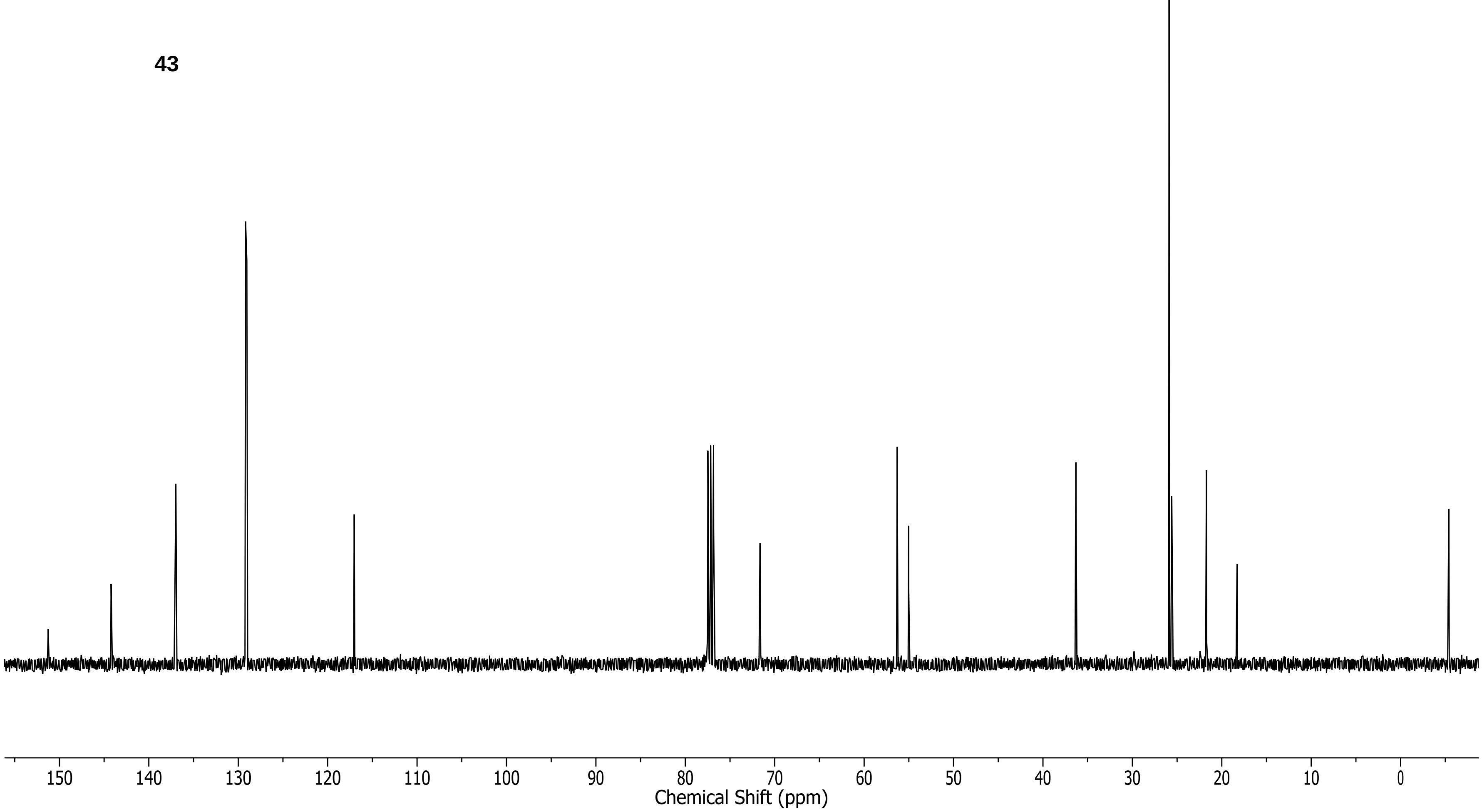
150
140
130
120
110
100
90
80
70
60
50
40
30
20
10
0
Chemical Shift (ppm)

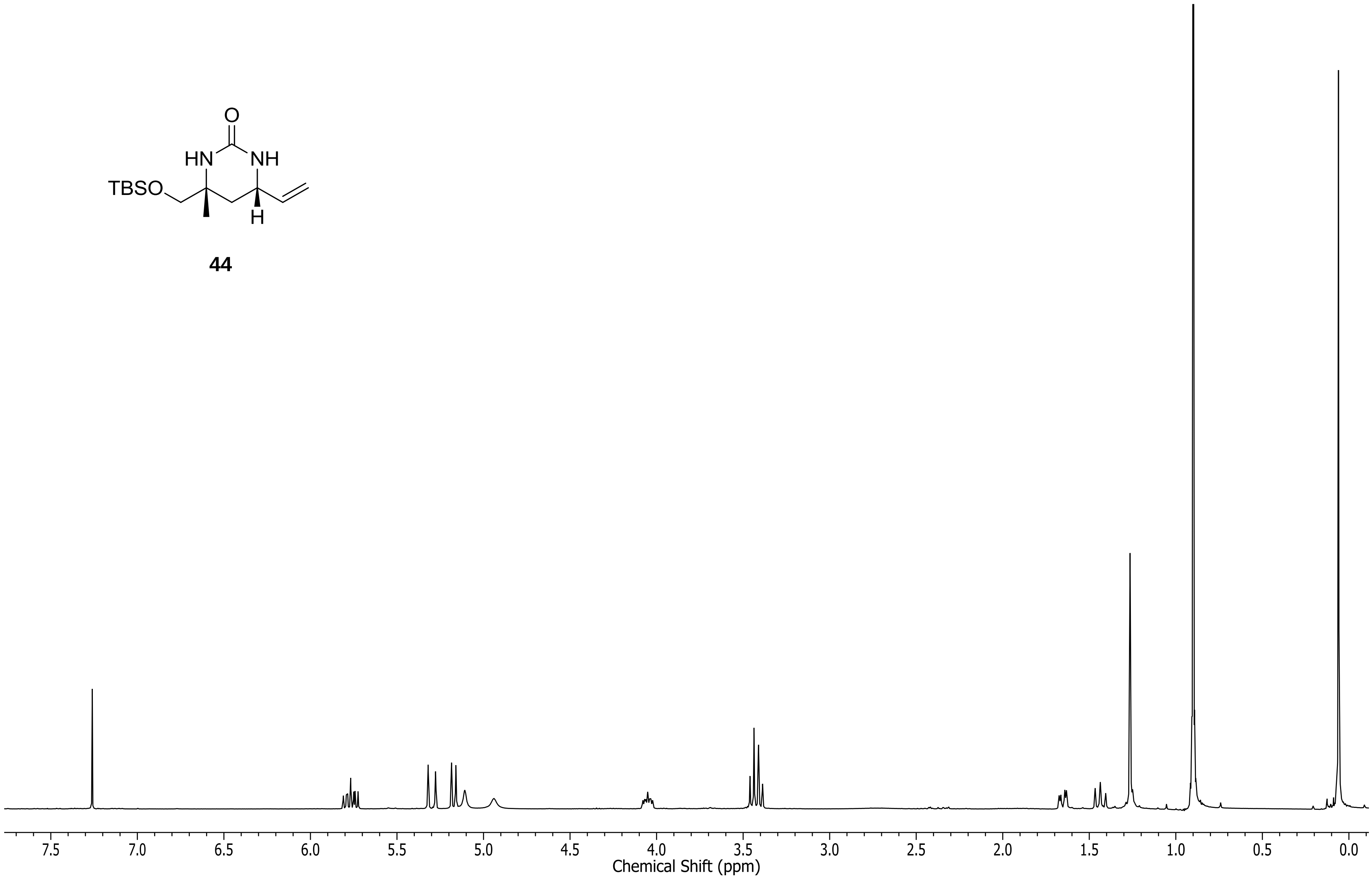

O
HN
NH
TBSO
H
44
7.5
7.0
6.5
6.0
5.5
5.0
4.5
4.0
3.5
3.0
2.5
2.0
1.5
1.0
0.5
0.0
Chemical Shift (ppm)

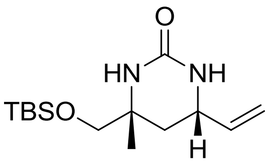

O
HN
NH
TBSO
H

44

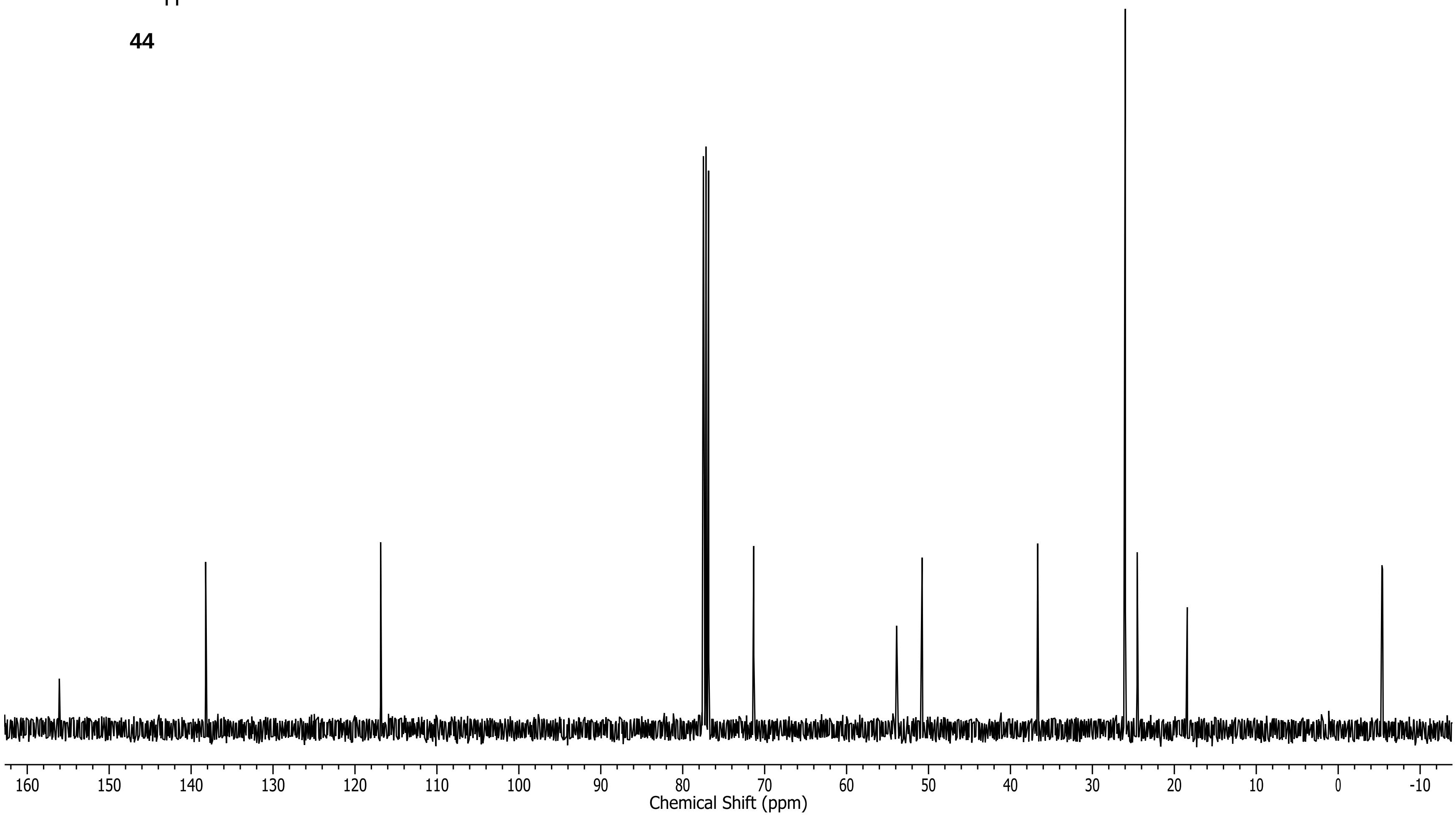

160
150
140
130
120
110
100
90
80
70
60
50
40
30
20
10
0
-10
Chemical Shift (ppm)

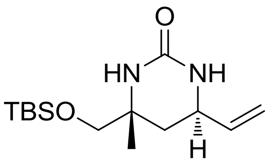
O
HN
NH
TBSO
H

45

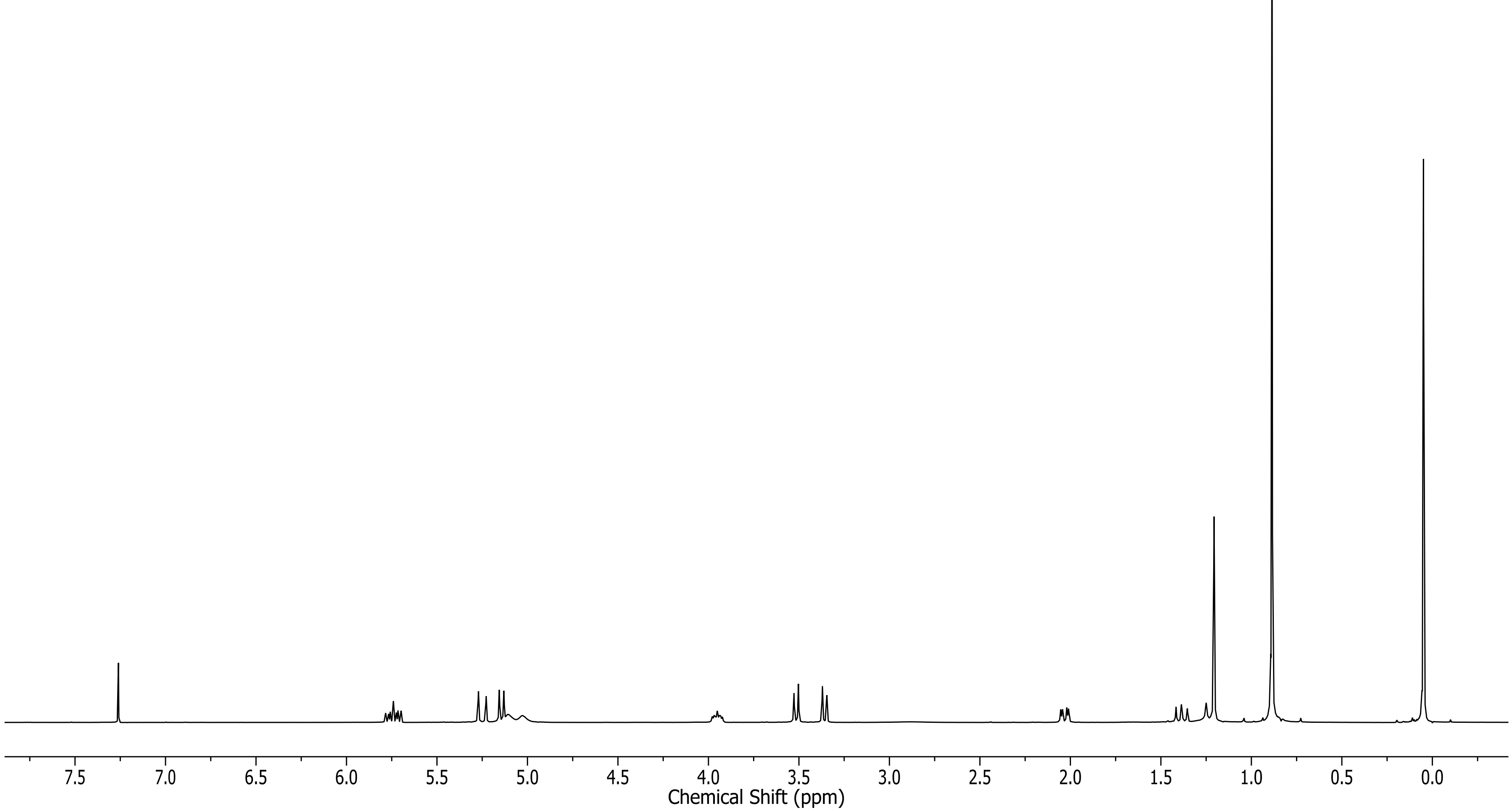
7.5
7.0
6.5
6.0
5.5
5.0
4.5
4.0
3.5
3.0
2.5
2.0
1.5
1.0
0.5
0.0
Chemical Shift (ppm)

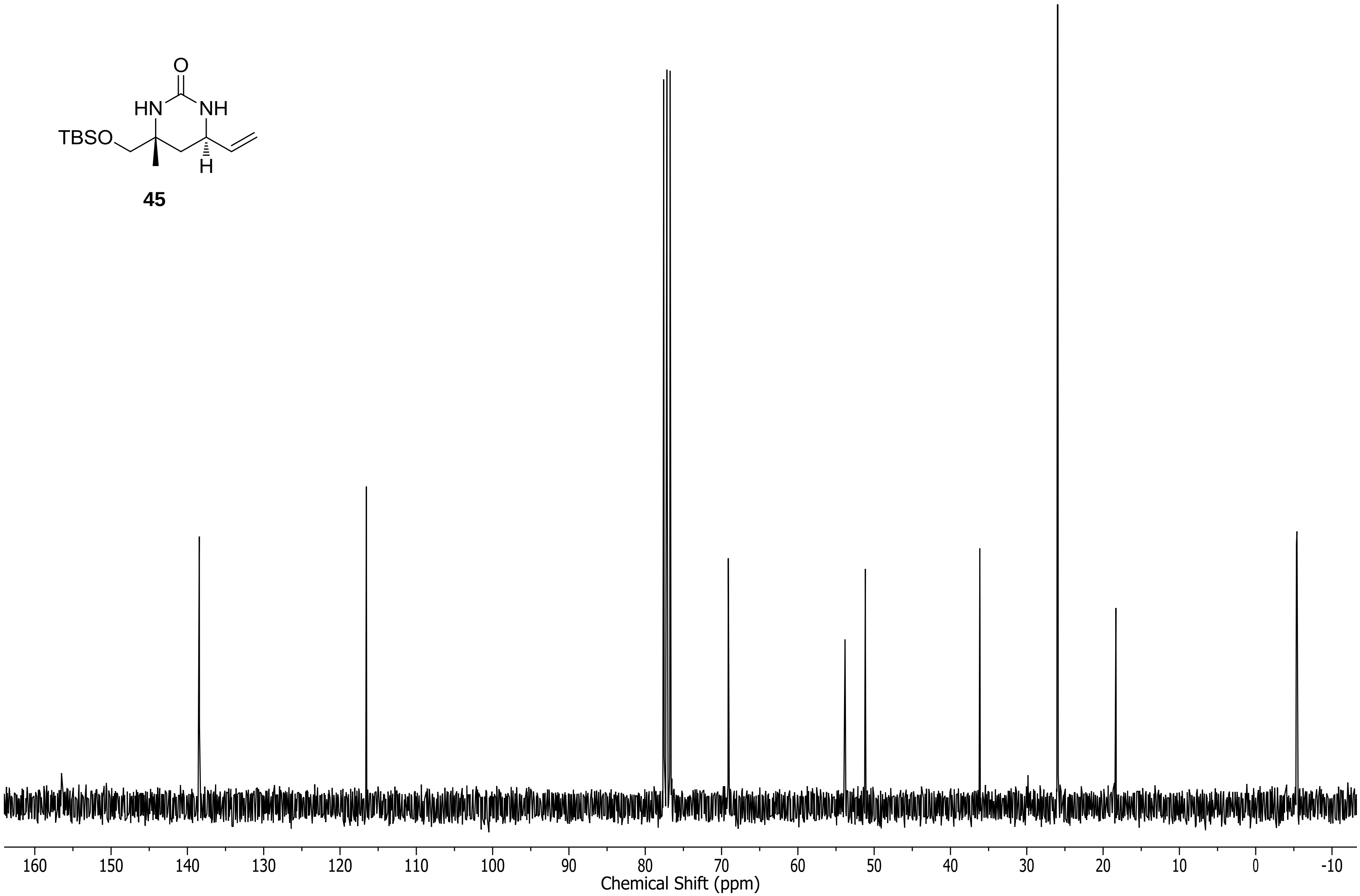
O
HN
NH
TBSO
H
45
160
150
140
130
120
110
100
90
80
70
60
50
40
30
20
10
0
-10
Chemical Shift (ppm)

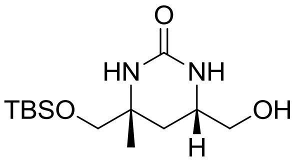

3

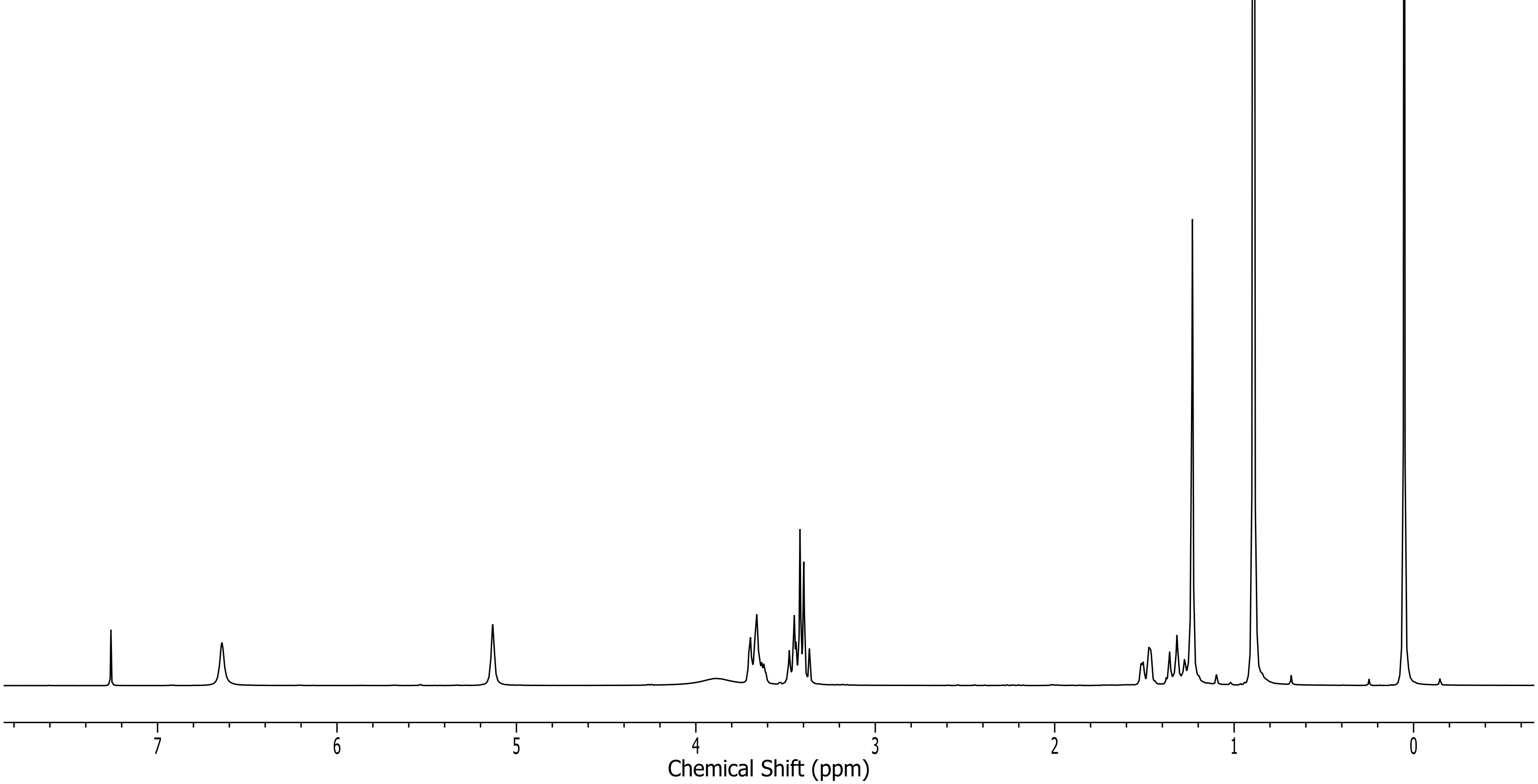

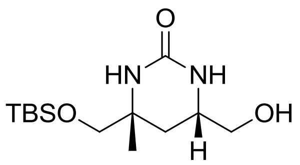
O
HN
NH
TBSO
OH
H

3

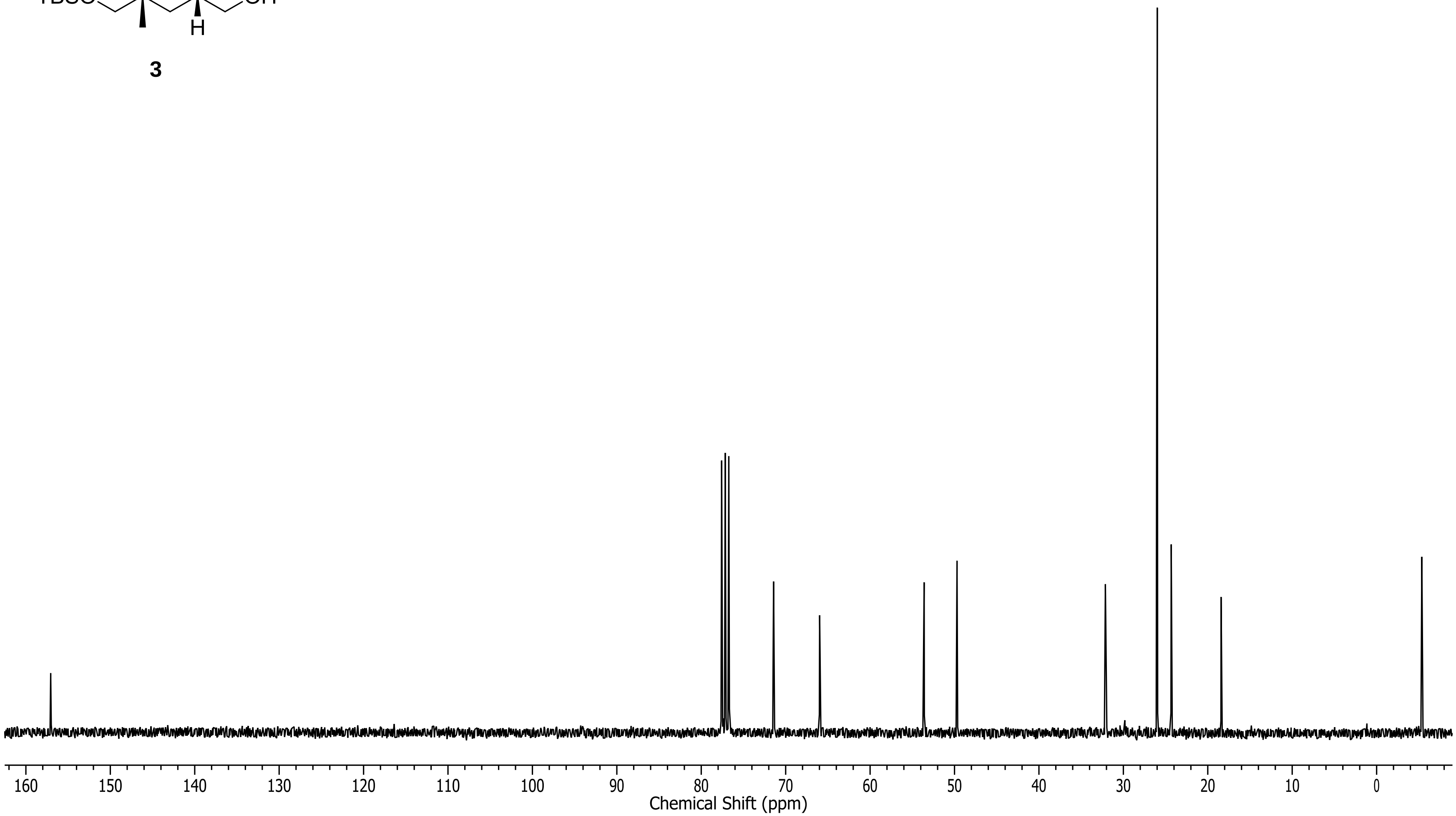
160
150
140
130
120
110
100
90
80
70
60
50
40
30
20
10
0
Chemical Shift (ppm)

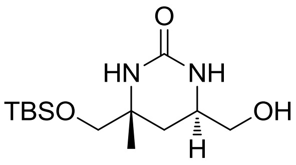
O
HN
NH
TBSO
OH
H

4

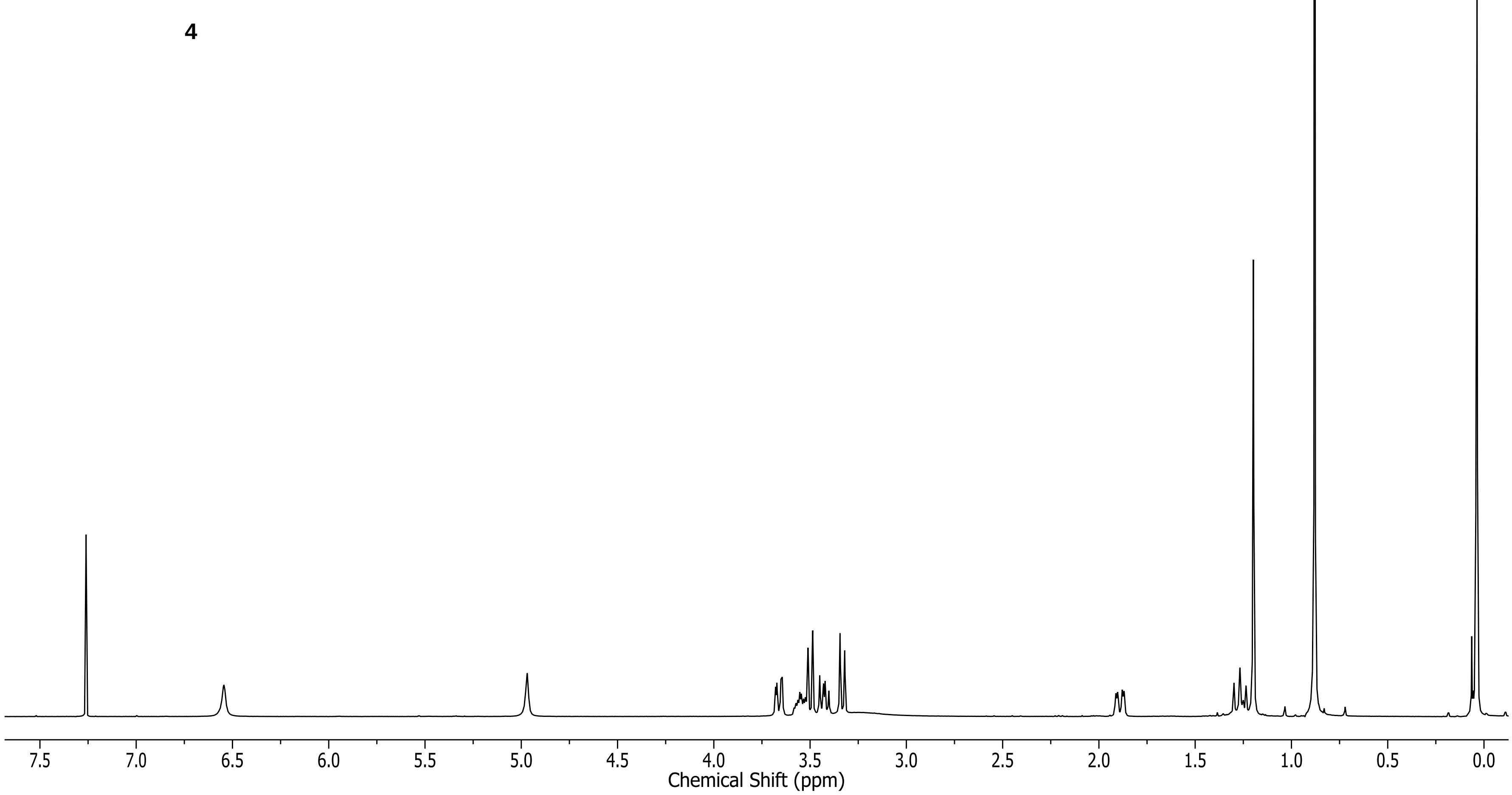
7.5
7.0
6.5
6.0
5.5
5.0
4.5
4.0
3.5
3.0
2.5
2.0
1.5
1.0
0.5
0.0
Chemical Shift (ppm)

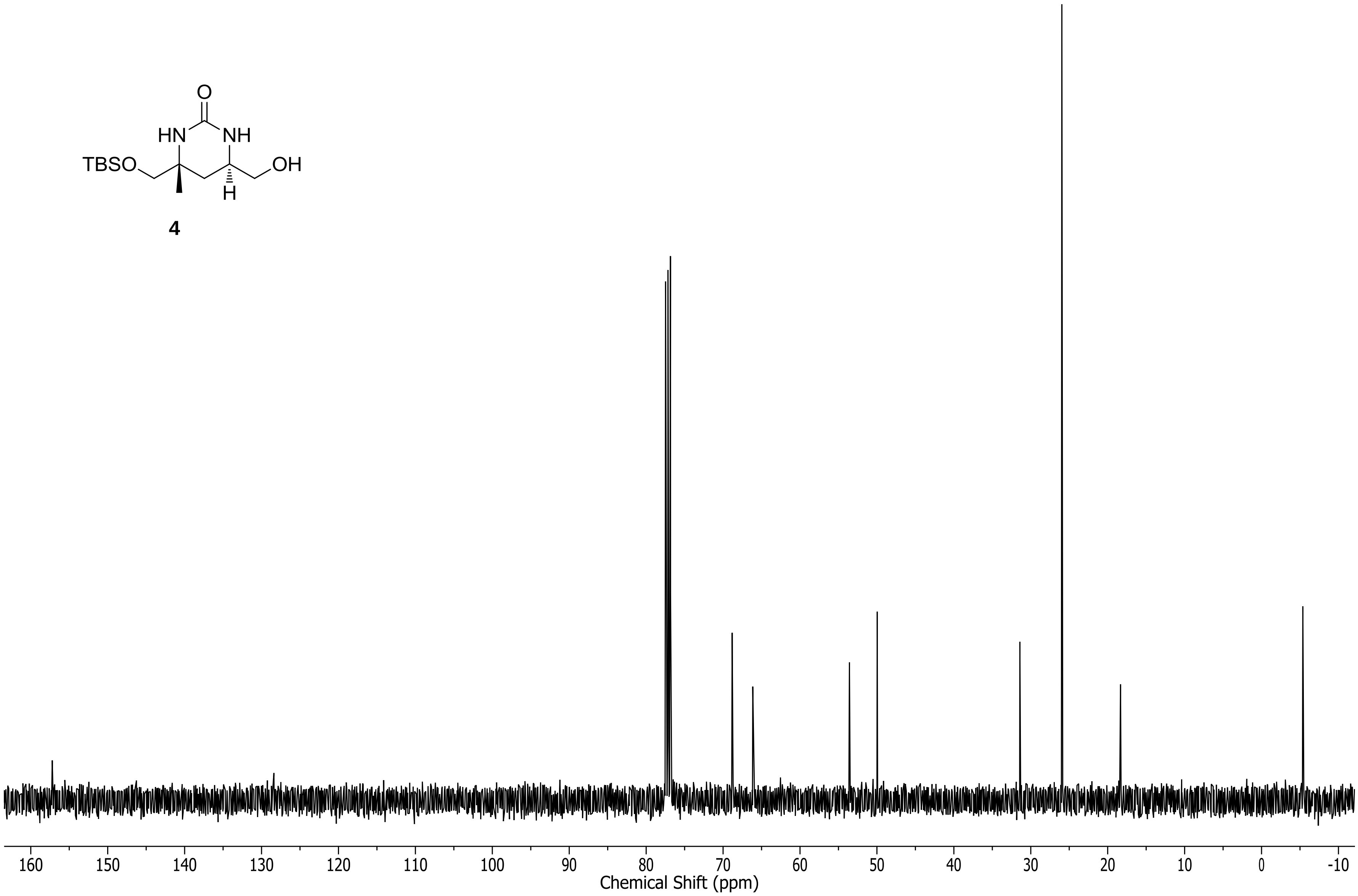
O
HN
NH
TBSO
OH
H

4

160
150
140
130
120
110
100
90
80
70
60
50
40
30
20
10
0
-10
Chemical Shift (ppm)

4. X-RAY DATA ANALYSIS

CCDC 1461907 (**28**), 1461908 (**32**), and 1461909 (**39**) contain the supplementary crystallographic data for this paper. These data can be obtained free of charge from The Cambridge Crystallographic Data Centre via www.ccdc.cam.ac.uk/data_request/cif.
